# A Dual‐Piezoelectric Metal–Organic Cage/COF Z‐Scheme Heterojunction for Solar‐Mechanical Water Splitting

**DOI:** 10.1002/advs.202523030

**Published:** 2026-01-04

**Authors:** Xin‐Ao Li, Yixuan Wang, Zi‐Zhan Liang, Xin‐Bang Peng, Li‐Min Xiao, Li Gong, Xinyi Yang, Bo Zou, Yecheng Zhou, Jun‐Min Liu

**Affiliations:** ^1^ The Key Laboratory of Low‐Carbon Chemistry & Energy Conservation of Guangdong Province School of Materials Science and Engineering Sun Yat‐sen University Guangzhou China; ^2^ Synergetic Extreme Condition High‐Pressure Science Center State Key Laboratory of Superhard Materials College of Physics Jilin University Changchun China; ^3^ School of Computer Science and Engineering Beihang University Beijing China; ^4^ Instrumental Analysis Research Center Sun Yat‐Sen University Guangzhou China

**Keywords:** covalent organic framework, metal–organic cage, photo‐piezocatalytic pure water splitting, piezoelectric materials, Z‐scheme photosystem

## Abstract

Developing efficient solar‐mechanical energy conversion systems is crucial for sustainable H_2_/H_2_O_2_ production. We report an unprecedented piezoelectric metal–organic cage (MOC‐FA3), integrated with piezoelectric β‐ketoenamine COF (S‐COF) into a Z‐scheme heterojunction via supramolecular interactions. The non‐centrosymmetric piezoelectricity of MOC‐FA3 is confirmed by multimodal characterization (KPFM, piezoelectric coefficient measurement, and in situ spectroscopy) and piezocatalytic H_2_/H_2_O_2_ production (334.3/203.6 µmol g^−1^ h^−1^). Under ultrasound (60 W, 40 kHz) and AM 1.5G, the heterojunction achieves exceptional synergistic pure water splitting at 1297.6 H_2_ and 1304.2 H_2_O_2_ µmol g^−1^ h^−1^, among the highest for COF‐based piezo‐photocatalysts, with 1.95‐fold and 2.06‐fold enhancements over S‐COF. This stems from Z‐scheme charge transfer coupled with piezoelectric‐field‐enhanced separation. Experimental results and DFT calculations identify Pd sites (H_2_ evolution) and C^2^‐C^3^ sites (2e^−^ water oxidation → H_2_O_2_), with in situ EPR confirming •OH intermediates. This work introduces MOCs as a new piezoelectric material class, demonstrating synergistic Z‐scheme/piezoelectric coupling for mechano‐photonic energy conversion.

## Graphical abstract

A dual‐piezoelectric Z‐scheme heterojunction is constructed by integrating a piezoelectric metal–organic cage (MOC‐FA3) with a β‐ketoenamine‐linked covalent organic framework (S‐COF). This design enables efficient solar‐mechanical energy conversion, achieving remarkable piezo‐photocatalytic pure water splitting for simultaneous H_2_ and H_2_O_2_ production without sacrificial agents, leveraging synergistic piezoelectric polarization and Z‐scheme charge transfer.

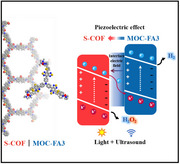

## 1 Introduction

Efficient water decomposition for hydrogen (H_2_) and hydrogen peroxide (H_2_O_2_) production is a promising renewable energy technology [1, 2, 3]. Current photocatalytic water splitting remains limited to H_2_ generation, which relies on sacrificial agents and cocatalysts in order to boost charge separation [4, 5]. Piezocatalysis, converting mechanical to chemical energy, offers an alternative for H_2_ and H_2_O_2_ synthesis. In non‐centrosymmetric materials, mechanical stress generates polarized surface charges and intrinsic electric fields, which can enhance the separation of photogenerated charge carriers and suppress recombination. Furthermore, piezoelectric polarization can induce interfacial band bending, enabling spatial charge separation and reducing the kinetic overpotential of the H_2_O_2_ evolution reaction, thereby boosting water splitting efficiency [6, 7]. Representative piezoelectric materials, including perovskites (e.g., BiFeO_3_, BaTiO_3_) and polymeric semiconductors (e.g., graphitic carbon nitride, g‐C_3_N_4_), have demonstrated efficacy in piezo‐catalytic water splitting systems [8, 9, 10, 11, 12, 13]. However, classical inorganic piezo‐catalysts suffer from low light‐energy coupling, limited structural tunability, and complex polarization, restricting their mechanical catalytic efficiency [14]. Therefore, developing piezoelectric photocatalysts possessing strong visible‐light absorption, enhanced piezoelectric coefficients, optimized band alignment, high charge carrier mobility, and suppressed recombination rates is imperative for achieving efficient visible‐light‐driven piezo‐photocatalytic conversion.

Covalent organic frameworks (COFs) exhibit significant promise in photocatalysis owing to their high surface area, modular design, crystallinity, photostability, and tunable bandgaps [15, 16, 17, 18, 19, 20]. However, rapid charge recombination and limited light absorption constrain their development [21, 22, 23]. Heterojunction construction with semiconductors effectively enhances charge separation [24, 25], particularly Z‐scheme architectures that enable broad‐spectrum absorption, efficient carrier separation, and strong redox potentials [26, 27]. Despite improved performance, most COF composites still require sacrificial agents for water splitting [28, 29]. Recent breakthroughs leverage structural versatility to engineer non‐centrosymmetric two‐dimensional (2D) COFs, where symmetry‐breaking dipole alignment synergistically boosts piezoelectricity for unprecedented photo‐piezocatalytic bifunctionality [30, 31]. Notably, we have reported the first demonstration of a highly crystalline β‐ketoenamine‐linked COF (EA‐COF) for efficient photo‐piezocatalytic generation of H_2_ and H_2_O_2_ in pure water without cocatalysts. This piezoelectric response originated primarily from in‐plane polarization of trihydroxybenzene‐tricarbaldehyde moieties [32].

Metal–organic cages (MOCs), three‐dimensional (3D) supramolecular assemblies formed by metal‐ligand coordination, have emerged as promising candidates for photocatalytic hydrogen evolution [33, 34]. Their molecularly integrated photosensitizers, electron relays, and catalytic sites enable efficient charge transfer. However, homogeneous MOCs suffer from poor stability and recyclability [35, 36]. Heterojunction construction addresses these limitations by enhancing carrier separation/migration [37, 38, 39]. Our group has developed semiconductor‐immobilized MOC systems (e.g., g‐C_3_N_4_ [40, 41, 42], TiO_2_ [43], COFs [44]) that outperformed homogeneous counterparts through synergistic electron transfer, extended visible‐light absorption, and atomically dispersed Pd sites. Building on our prior findings, the EA‐COF with piezoelectric properties, upon composite with MOC‐Q3, demonstrated an increase in piezo‐photocatalytic performance for H_2_ and H_2_O_2_ production [32]. However, the MOC‐Q3 used in this study lacked piezoelectric properties, resulting in insufficient performance enhancement of the composite material. To overcome this bottleneck, the development of non‐centrosymmetric MOCs with intrinsic piezoelectricity is crucial, yet the piezoelectric effect in MOCs remains unexplored, and its synergistic coupling with piezoelectric COFs for energy conversion is unprecedented.

**FIGURE 1:**
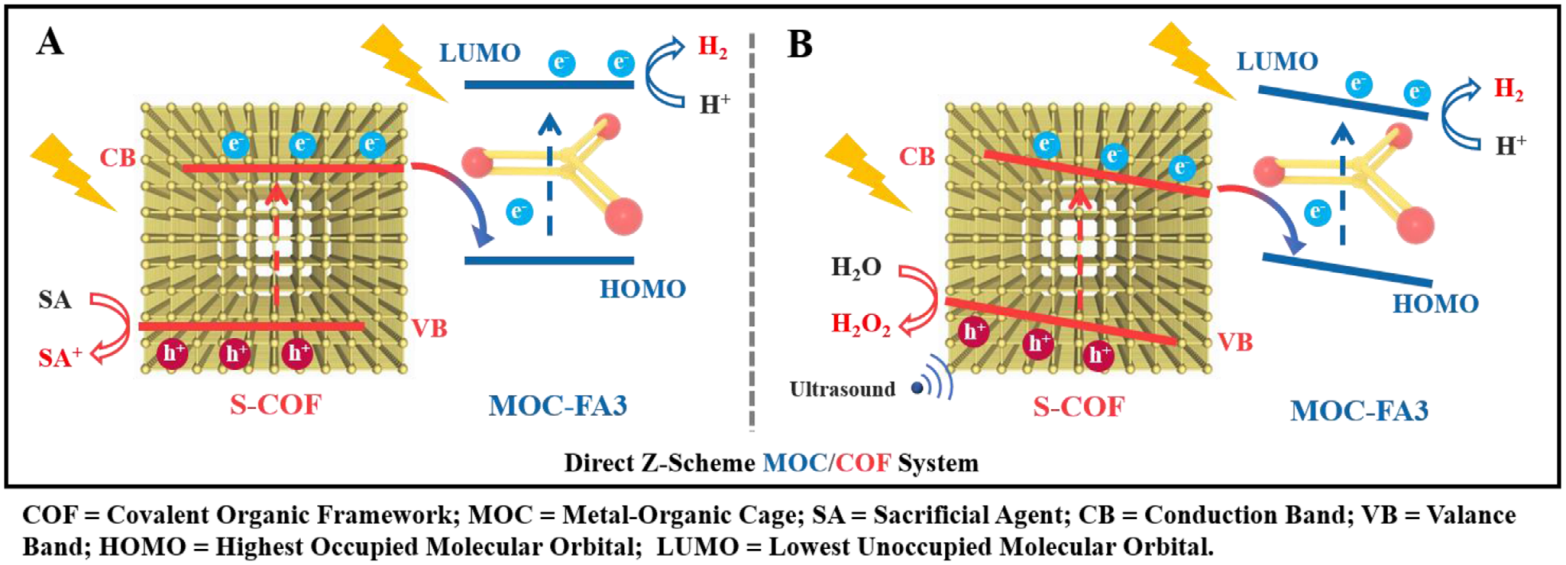
Mechanistic diagram of Z‐scheme MOC‐FA3/S‐COF heterojunction for water splitting. (A) Photocatalytic pathway under visible light. (B) Synergistic piezo‐photocatalytic pathway under concurrent ultrasound/light.

Herein, by engineering molecular asymmetry into a metal–organic cage (MOC‐FA3), we pioneered intrinsic piezoelectricity in supramolecular systems, breaking classical symmetry constraints. This enabled the construction of a dual‐piezoelectric Z‐scheme heterojunction with a β‐ketoenamine‐based COF (S‐COF), where mechanically amplified built‐in fields and spatially directed electron transfer synergistically overcame charge recombination. Ultimately, orchestrating solar and mechanical stimuli at atomic Pd/C^3^‐C^2^ active sites achieved record H_2_/H_2_O_2_ co‐production, establishing mechanophotonics as a new paradigm for sustainable energy conversion (Figure 1). Under visible light irradiation and ascorbate sacrificial conditions (Figure 1A), this architecture achieved exceptional photocatalytic H_2_ evolution (1266 mmol·g^−1^ over 20 h with a turnover number of 180,842), surpassing all control systems. Density functional theory (DFT) computations and spectroscopic characterizations confirmed Z‐scheme electron transfer from S‐COF to MOC‐FA3 as the efficiency origin. Under piezophotocatalytic water splitting conditions, MOC‐FA3 generated H_2_ (334.3 µmol g^−1^ h^−1^) and H_2_O_2_ (203.6 µmol g^−1^ h^−1^). Notably, this work constituted the first report of piezophotocatalysis in MOCs. Under concurrent ultrasonic (40 kHz, 60 W) and AM 1.5G illumination (Figure 1B), the MOC‐FA3/S‐COF heterojunction exhibited synergistic enhancement, achieving H_2_ and H_2_O_2_ evolution rates of 1297.6 and 1304.2 µmol g^−1^ h^−1^, respectively, exceeding outputs of pristine S‐COF by 1.95 and 2.06‐fold. Kelvin probe force microscopy (KPFM), piezoelectric coefficient measurements, in situ pressure‐dependent UV–vis absorption, photoluminescence, and infrared spectroscopy, and theoretical modeling verified the piezoelectric response of MOC‐FA3, S‐COF, and MOC‐FA3/S‐COF.

## 2 Results and Discussion

### 2.1 Sample Synthesis and Characterization

**FIGURE 2:**
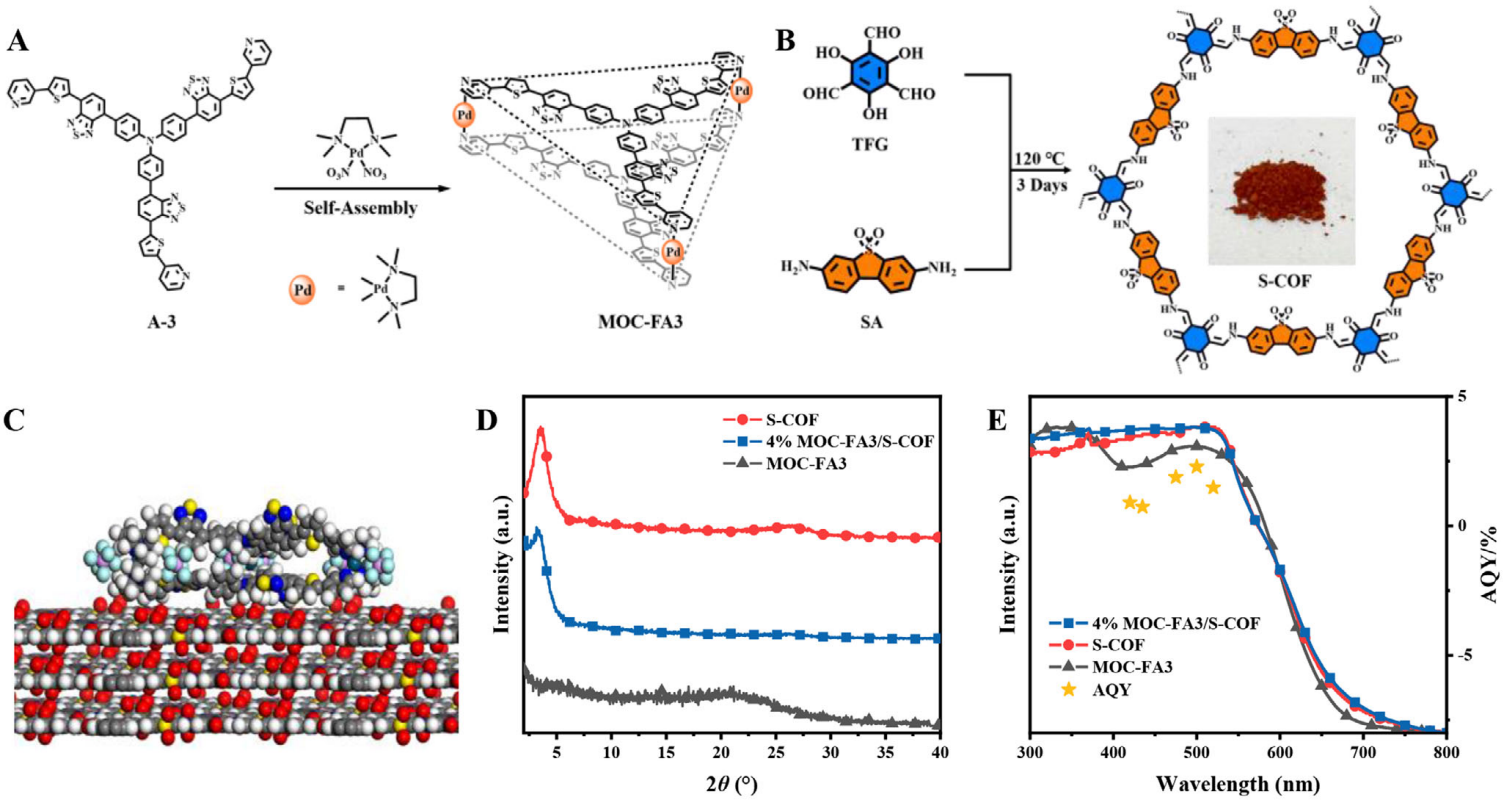
Synthesis, structural simulation, and characterization of S‐COF, MOC‐FA3, and the composite 4% MOC‐FA3/S‐COF. (A) Self‐assembly of MOC‐FA3 using ligand A‐3 and Pd^2^⁺ ions. (B) Synthesis of S‐COF through condensation of TFG and SA. (C) Simulated structure of the MOC‐FA3/S‐COF composite. (D) PXRD patterns of S‐COF, MOC‐FA3, and the 4% MOC‐FA3/S‐COF composite. (E) Solid UV–vis absorption spectra of S‐COF, MOC‐FA3, and 4% MOC‐FA3/S‐COF, and AQYs of 4% MOC‐FA3/S‐COF.

MOC‐FA3 was constructed via a self‐assembly strategy involving the A‐3 ligand and Pd metal ions (Figure 2A). The detailed synthetic procedures for ligands A‐1 to A‐3, as well as MOC‐FA3, could be found in the Experimental Section of the Supporting Information. Figure S1 illustrates the specific synthetic route of MOC‐FA3. The ^1^H nuclear magnetic resonance (NMR) spectra of ligands A‐1 to A‐3 and MOC‐FA3 (Figure S2), together with the ^13^C NMR and electrospray ionization mass spectrometry (ESI‐MS) spectra of ligands A‐1 to A‐3 (Figures S3 and S4), provided evidence to support the structural characterization of the synthesized compounds. A comparison of the ^1^H NMR spectra of ligand A‐3 and MOC‐FA3 revealed significant changes in the chemical shifts observed, which further confirmed the successful self‐assembly of MOC‐FA3 (Figure S5). Additionally, ^1^H diffusion ordered spectroscopy (DOSY) analysis was performed on MOC‐FA3 (Figure S6). This demonstrated that all signals possessed a uniform diffusion coefficient of 8.50 × 10^−10^ m^2^/s, indicating that MOC‐FA3 was a single, homogeneous component. Quantitative analysis of the diffusion coefficient based on the Stokes‐Einstein equation yielded a calculated hydrodynamic radius of 11.7 Å for MOC‐FA3. This further supported the uniformity and structural integrity of the self‐assembled MOC‐FA3. ESI‐MS analysis (Figure S7) was performed to further confirm the structure of MOC‐FA3. The spectra revealed charge states ranging from 2+ to 6+, indicating that MOC‐FA3 had lost 2 to 6 nitrate anions. This observation provided further evidence of the successful assembly of MOC‐FA3 and its structural integrity. Simulations optimized the structure of MOC‐FA3 to show a triangular prism shape with an edge length (Pd‐Pd) of ∼26.8 Å and a height of ∼5.0 Å (Figure S8). The chemical structure of MOC‐FA3 was characterized using Fourier‐transform infrared (FT‐IR) spectroscopy (Figure S9A).

S‐COF was synthesized using a solvothermal method with 2,4,6‐trihydroxy‐1,3,5‐benzenetricarbaldehyde (TFG) and 5,5‐dioxodibenzo[b,d]thiophene‐3,7‐diamine (SA) as the starting materials (Figure 2B). The detailed synthetic procedure is provided in the Experimental Section of the Supporting Information. The chemical structure of the synthesized sample was characterized using FT‐IR spectroscopy. The FT‐IR spectrum revealed the C═O stretching vibration peak of the ‐CHO groups in TFG at 1643 cm^−1^, and the stretching vibration peaks of the two NH_2_ groups in SA at 3476 and 3373 cm^−1^. Following S‐COF synthesis, these characteristic peaks disappeared, and new peaks corresponding to C═O and C═C stretching vibrations appeared at 1619 and 1577 cm^−1^, respectively (Figure S9B). Further analysis of the chemical environment of S‐COF was conducted using ^13^C NMR spectroscopy (Figure S9C,D). The ^13^C NMR spectrum revealed resonance signals at 188 ppm (corresponding to carbonyl carbon C^a^) and 108 ppm (assigned to secondary amine carbon C^b^). Additionally, a signal at 148 ppm, characteristic of the carbon atom associated with the ─NH─CH═ group C^c^, was identified. Powder X‐ray diffraction (PXRD) analysis was used to verify the crystalline structure of S‐COF (Figure S10A). The PXRD pattern revealed a strong diffraction peak at 3.47°, alongside a weaker peak at 6.87°, which corresponded to the (100) and (200) planes of S‐COF, respectively. Furthermore, a comparison of the experimental XRD pattern with simulated patterns of two different stacking modes confirmed that the crystalline structure of S‐COF exhibited AA stacking (Figure S10B), which was in good agreement (R_wp_ and R_p_ < 10%) with the Pawley refinement result in space group *P‐6* (Table S1).

A solution impregnation method was used to prepare 1%–5% MOC‐FA3/S‐COF composites, and inductively coupled plasma mass spectrometry (ICP‐MS) was used to determine their actual loading amounts (Table S2). Figure 2C shows the simulated structure of MOC‐FA3/S‐COF. MOC‐FA3 was loaded onto the S‐COF surface via hydrogen bonding (X∙∙∙C─H, X = S, N, O) and *π–π* interactions. XRD patterns (Figure 2D) revealed no significant diffraction peaks for MOC‐FA3, indicating its amorphous nature. The pristine S‐COF sample exhibited a sharp diffraction peak at 3.46°, whereas the composite material showed a slightly weaker peak at the same position. This suggested that the addition of MOC‐FA3 did not disrupt the primary structure of S‐COF, although it slightly reduced the crystallinity of the framework.

Scanning electron microscopy (SEM) images (Figure S11A,B) showed that both the 4% MOC‐FA3/S‐COF and the S‐COF samples retained their layered morphology and that there was no noticeable structural damage caused by loading the MOC‐FA3. Similarly, transmission electron microscopy (TEM) results (Figure S11C,D) confirmed that both samples exhibited a layered structure, with no discernible differences between them. TEM elemental analysis of S‐COF revealed a uniform distribution of atoms (Figure S12, Table S3), and TEM elemental mapping results for 4% MOC‐FA3/S‐COF (Figure S13, Table S4) confirmed a uniform distribution of C, N, O, S, and Pd atoms. The Pd mass fraction was consistent with the results of ICP‐MS. Furthermore, aberration‐corrected high‐angle annular dark‐field scanning transmission electron microscopy (AC‐HAADF‐STEM) revealed isolated Pd atoms as single bright spots, which were evenly distributed across the S‐COF surface (Figure S14).

The porosity of the samples was evaluated using N_2_ adsorption–desorption isotherms at 77 K (Figure S15). The Brunauer‐Emmett‐Teller (BET) surface areas of S‐COF and MOC‐FA3/S‐COF were found to be 834 and 580 m^2^/g, respectively. The pore sizes derived from fitting the isotherms to a non‐local DFT model were 2.00 and 2.05 nm, respectively. This indicated that MOC‐FA3 uniformly covered the surface of S‐COF, resulting in a reduction in surface area without affecting the crystalline structure. The solid‐state UV–vis absorption spectra of MOC‐FA3, S‐COF, and 4% MOC‐FA3/S‐COF were measured (Figure 2E). Both MOC‐FA3 and S‐COF exhibited broad absorption bands in the 350–550 nm range, with absorption edges around 650 nm. This implied that they both displayed good visible light absorption abilities. The UV–vis spectra of the composites showed no significant changes compared to those of the individual components. The FT‐IR spectra of 4% MOC‐FA3/S‐COF and S‐COF also exhibited minimal differences due to the relatively low loading of MOC‐FA3 (Figure S16). Analysis of the Pd 3*d* signals using X‐ray photoelectron spectroscopy (XPS) indicated that Pd existed in a +2 oxidation state on the surface of S‐COF (Figure S17). Compared to pure MOC‐FA3, the Pd 3*d* XPS peaks in the composite shifted to lower binding energies. This shift was attributed to the electron‐rich environment, which reduced the binding energy of the palladium electrons. This further confirmed that MOC‐FA3 successfully interacted with S‐COF through supramolecular interactions.

### 2.2 Photocatalytic H_2_ Production Performance and Mechanism

**FIGURE 3:**
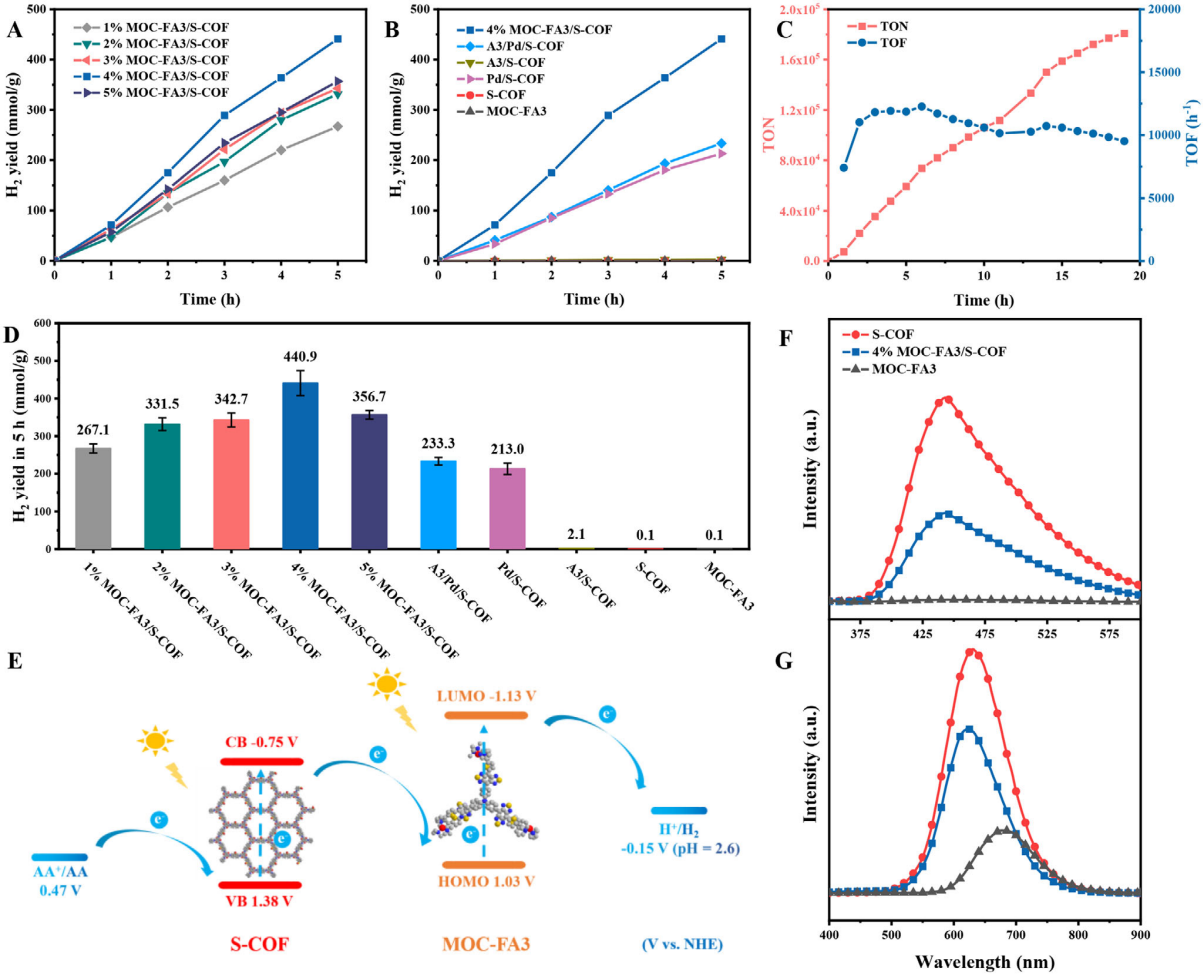
Catalytic performance and mechanistic insights of the MOC‐FA3/S‐COF system for photocatalytic water splitting. (A) Time‐dependent H_2_ evolution yields for MOC‐FA3/S‐COF composites with varying MOC‐FA3 loading percentages under visible‐light irradiation (λ > 420 nm, Xe lamp). (B) Comparative time‐dependent H_2_ evolution yields for reference samples under identical irradiation conditions (λ > 420 nm, Xe lamp, 5 h). (C) TON and TOF calculated for 4% MOC‐FA3/S‐COF over an extended 20‐h reaction period. (D) Total H_2_ evolution amounts for all tested samples after 5 h of irradiation. (E) Proposed working mechanism of the MOC‐FA3/S‐COF composite system for photocatalytic water splitting. (F) Liquid‐phase PL spectra of TA‐OH adducts formed during photocatalytic •OH trapping, excited at 315 nm. (G) Steady‐state solid‐state PL spectra of MOC‐FA3, S‐COF, and the 4% MOC‐FA3/S‐COF composite.

The photocatalytic hydrogen evolution performance of MOC‐FA3/S‐COF loaded with 1‐5 wt.% MOC‐FA3 was tested under visible light using ascorbic acid (AA) as a sacrificial electron donor (Figure 3A). The H_2_ evolution performance of the MOC‐FA3/S‐COF was found to increase with rising MOC‐FA3 content, reaching a maximum value of 88.2 mmol g^−1^ h^−1^ at a loading of 4 wt.%. However, the H_2_ evolution rate decreased when the loading increased to 5 wt.%. Ultimately, the H_2_ evolution rates of the samples followed the order: 1% < 2% < 3% < 5% < 4% MOC‐FA3/S‐COF. The lower activity of the 5% sample could be due to MOC‐FA3 molecules aggregating on the COF surface. This weakened the *π–π* supramolecular interactions between MOC‐FA3 and S‐COF, making it harder for MOC‐FA3 to load effectively onto the S‐COF surface. The apparent quantum yields (AQYs) of the 4% MOC‐FA3/S‐COF composite were measured under monochromatic light irradiation. The resulting AQY values (Table S5) correlated well with the light absorption profile observed in its UV–vis absorption spectrum (Figure 1E) across the measured wavelengths.

Figure 3B shows the photocatalytic H_2_ production performance of 4% MOC‐FA3/S‐COF, A‐3/Pd/S‐COF, Pd/S‐COF, A‐3/S‐COF, bare S‐COF, and MOC‐FA3, with comparative data shown in Figure 3D. MOC‐FA3 and S‐COF exhibited poor H_2_ production activity due to its low efficiency in separating photogenerated charge carriers and rapid electron‐hole recombination. In contrast, Pd/S‐COF, which was loaded with Pd nanoparticles, demonstrated better H_2_ production performance, indicating that metallic Pd served as an active site in the system. A‐3/Pd/S‐COF, which incorporated a small amount of the organic dye A‐3, marginally improved the light absorption capacity of the composite and thus the H_2_ production performance compared to Pd/S‐COF. However, the photocatalytic efficiency of these materials was much lower than that of 4% MOC‐FA3/S‐COF. This highlighted that the exceptional activity of the composite was due to the combination of MOC‐FA3 and S‐COF.

The long‐term stability of the material was investigated, revealing that the photocatalytic system maintained good stability for 20 h of continuous reaction under visible light (Figure 3C), achieving an H_2_ production rate of 1266 mmol g^−1^. Based on the Pd content, the cumulative turnover number (TON) was calculated to be 180 842, with a turnover frequency (TOF) that remained stable at approximately 10 000 h^−1^. XPS analysis confirmed that the oxidation state of Pd in MOC‐FA3/S‐COF did not change during the reaction compared to its initial state (Figure S18A). PXRD analysis of the used MOC‐FA3/S‐COF was conducted and compared with the pristine sample (Figure S18B). The diffraction peak at 3.46° was found to be weaker, indicating that photoreduction had affected the crystal phase of the S‐COF. Additionally, the solid‐state UV–vis absorption spectra (Figure S18C) and FT‐IR spectra (Figure S18D) of both samples were measured. No significant changes were observed in the spectra, suggesting that MOC‐FA3/S‐COF retained its original molecular structure after photocatalysis. These results demonstrated that MOC‐FA3/S‐COF exhibited excellent photocatalytic activity and stability.

To investigate the photocatalytic mechanism of MOC‐FA3/S‐COF, cyclic voltammetry (CV) and optical tests were performed on MOC‐FA3. Based on the CV results, the highest occupied molecular orbital (HOMO) value of MOC‐FA3 was determined to be 1.03 V vs. normal hydrogen electrode (NHE). Additionally, the bandgap of 2.16 eV was calculated from the intersection of the UV–vis absorption and fluorescence spectra, giving a lowest unoccupied molecular orbital (LUMO) value of −1.13 V vs. NHE (Figure S19, Table S6). Mott‐Schottky analysis (Figure S20A) revealed that the flat‐band potential of S‐COF was −0.48 V vs. NHE, corresponding to its Fermi level (*E*_f_). The valence band X‐ray photoelectron spectroscopy (VB‐XPS) analysis of S‐COF (Figure S20B) showed that the energy difference between the *E*_f_ and the valence band (VB) was 1.86 eV. This enabled the valence band (VB) potential of S‐COF to be estimated at 1.38 V vs. NHE. Using the VB potential and the 2.13 eV bandgap of S‐COF (Figure S20C), its conduction band (CB) potential was estimated to be −0.75 V vs. NHE (Figure S20D). Based on these results, an energy level diagram of MOC‐FA3/S‐COF was constructed, as shown in Figure 3E. The CB of S‐COF had a more negative potential than the HOMO level of MOC‐FA3. The above results indicated that Z‐scheme electron transfer from the CB of S‐COF to the HOMO of MOC‐FA3 was thermodynamically feasible. Furthermore, the H⁺/H_2_ reduction potential exceeded that of the LUMO of MOC‐FA3, enabling MOC‐FA3 to accept transferred electrons under visible light and drive H_2_ production at its Pd catalytic sites. Additionally, the redox potential of ascorbic acid (AA) (0.47 V vs. NHE) was more negative than the VB edge of S‐COF, allowing AA to effectively quench the holes on S‐COF. Therefore, dual light excitation of S‐COF and MOC‐FA3, along with the Z‐scheme charge transfer process, could ensure the efficient spatial separation of electron‐hole pairs under visible light. This promoted sustained, highly efficient photocatalytic H_2_ production in the presence of a sacrificial agent.

To verify the charge transfer path, the energy levels of S‐COF and MOC‐FA3 were obtained through theoretical calculations. The calculated VB and CB of S‐COF were 1.41 and −0.93 V vs. NHE, respectively. For molecular MOC‐FA3, the calculated HOMO and LUMO were 1.31 and −0.98 V vs. NHE, respectively. These general energy levels were consistent with the experimental results (Figure S21).

To confirm the proposed Z‐scheme electron transfer mechanism, •OH trapping experiments were conducted using terephthalic acid (TA) on S‐COF, MOC‐FA3, and 4% MOC‐FA3/S‐COF. As the VB potential of S‐COF was 1.38 V vs. NHE and thus more negative than the oxidation potential of •OH/OH^−^ (+2.70 V vs. NHE), •OH could not be generated from the h⁺ in the VB of S‐COF. Instead, the e^−^ in the CB of S‐COF could interact with oxygen to form •OH via a two‐electron oxidation pathway. This process is described by Equation S1 (Figure S22). Terephthalic acid (TA) reacted with •OH to produce 2‐hydroxyterephthalic acid (TA‐OH), a highly fluorescent compound exhibiting a characteristic fluorescence peak at 425 nm when excited at 315 nm. Therefore, the relative fluorescence intensity of the TA‐OH complex could be used to compare the amount of •OH generated in the system.

As shown in Figure 3F, the fluorescence intensity of the TA solution containing S‐COF was higher than that of the TA solution incorporating 4% MOC‐FA3/S‐COF. In contrast, the TA solution comprising MOC‐FA3 exhibited only weak fluorescence. This indicated that •OH generation occurred exclusively through reactions involving electrons from the CB of S‐COF rather than MOC‐FA3. Furthermore, under visible light irradiation, photoelectrons transferred from the CB of S‐COF to the HOMO of MOC‐FA3 in the 4% MOC‐FA3/S‐COF solution. This reduced the number of photogenerated electrons available in the CB of S‐COF, leading to decreased •OH generation and lower observed fluorescence intensity.

Steady‐state photoluminescence (PL) spectra were measured for the 4% MOC‐FA3/S‐COF, MOC‐FA3, and S‐COF samples in order to further elucidate the Z‐scheme electron transfer mechanism (Figure 3G). Under 365 nm excitation, the fluorescence peak of bare S‐COF was strongest at approximately 650 nm, which was attributed to the intense recombination of photogenerated electron‐hole pairs. By contrast, MOC‐FA3 showed the weakest fluorescence, with a peak near 677 nm corresponding to the ligand‐to‐metal charge transfer transition. Combining the two components resulted in photoelectrons from S‐COF transferring to MOC‐FA3, significantly reducing the fluorescence intensity of the 4% MOC‐FA3/S‐COF sample.

Surface photovoltage (SPV) measurements were conducted to investigate the photogenerated charge separation and transfer processes occurring at the surface and interfaces of MOC‐FA3/S‐COF (Figure S23). Solid‐state UV–vis absorption spectra revealed comparable absorption capacities in the visible light range for 4% MOC‐FA3/S‐COF and S‐COF, while 4% MOC‐FA3/S‐COF showed a significantly enhanced photovoltage signal in the 300–550 nm region. This indicated that MOC‐FA3/S‐COF achieved more effective spatial separation and transfer of photoelectrons. Furthermore, transient absorption spectroscopy (TAS) measurements were also performed on S‐COF and MOC‐FA3/S‐COF. Both samples exhibited broad negative bleaching signals in the wavelength range of 450–650 nm, which were closely associated with the generation of excited‐state electrons (Figure S24A,B). By fitting the kinetic curves of S‐COF and MOC‐FA3/S‐COF, two sets of lifetime parameters were obtained: the short‐lived component (τ_1_) corresponded to the electron diffusion process between lattices, while the long‐lived component (τ_2_) originated from the recombination behavior of photogenerated electrons and holes. Both τ_1_ (0.36 ps) and τ_2_ (17.15 ps) of MOC‐FA3/S‐COF were significantly shorter than the corresponding values of pristine S‐COF (τ_1_ = 1.29 ps, τ_2_ = 30.11 ps) (Figure S24C,D). This implied that the formation of MOC‐FA3/S‐COF heterojunctions reduced the number of photogenerated electrons trapped in CB of S‐COF, thereby shortening the electron lifetime. This phenomenon confirmed the electron migration process from the CB of S‐COF to HOMO of MOC‐FA3 [45, 46].

To gain further insight into these phenomena, the photocurrent‐time (*i*‐*t*) curves (Figure S25) were investigated, the photocurrent densities of the samples were found to follow the order: MOC‐FA3< S‐COF < 4% MOC‐FA3/S‐COF. This correlated well with their photocatalytic hydrogen evolution performance. To evaluate the charge carrier dynamics, electrochemical impedance spectroscopy (EIS) measurements were performed in the dark (Figure S26, Table S7) and under light irradiation (Figure S27, Table S8). As indicated by the equivalent circuit fitting, R_1_ represents the combined electrolyte and internal ohmic resistance, while R_2_ corresponds to the interfacial charge transfer resistance under light or, in the dark, primarily reflects charge recombination resistance at the sample/electrolyte interface. Comparative analysis of the EIS spectra of the three samples revealed that under dark conditions, the semicircle radius of the EIS Nyquist plot and the R_2_ value increased in the following order: MOC‐FA3 (10910 Ω) < S‐COF (21777 Ω) < 4% MOC‐FA3/S‐COF (25500 Ω). Meanwhile, under light irradiation, the semicircle radius of the EIS Nyquist plot and the R_2_ value decreased in the following order: MOC‐FA3 (7113 Ω) > S‐COF (6274 Ω) > 4% MOC‐FA3/S‐COF (3915 Ω). This indicated that 4% MOC‐FA3/S‐COF possessed lower solid‐phase interface resistance and superior charge separation and transfer capabilities. These results confirmed the existence of the Z‐scheme electron transfer mechanism, which could effectively enhance the photocatalytic performance of the material.

### 2.3 Photopiezoelectric Properties

**FIGURE 4:**
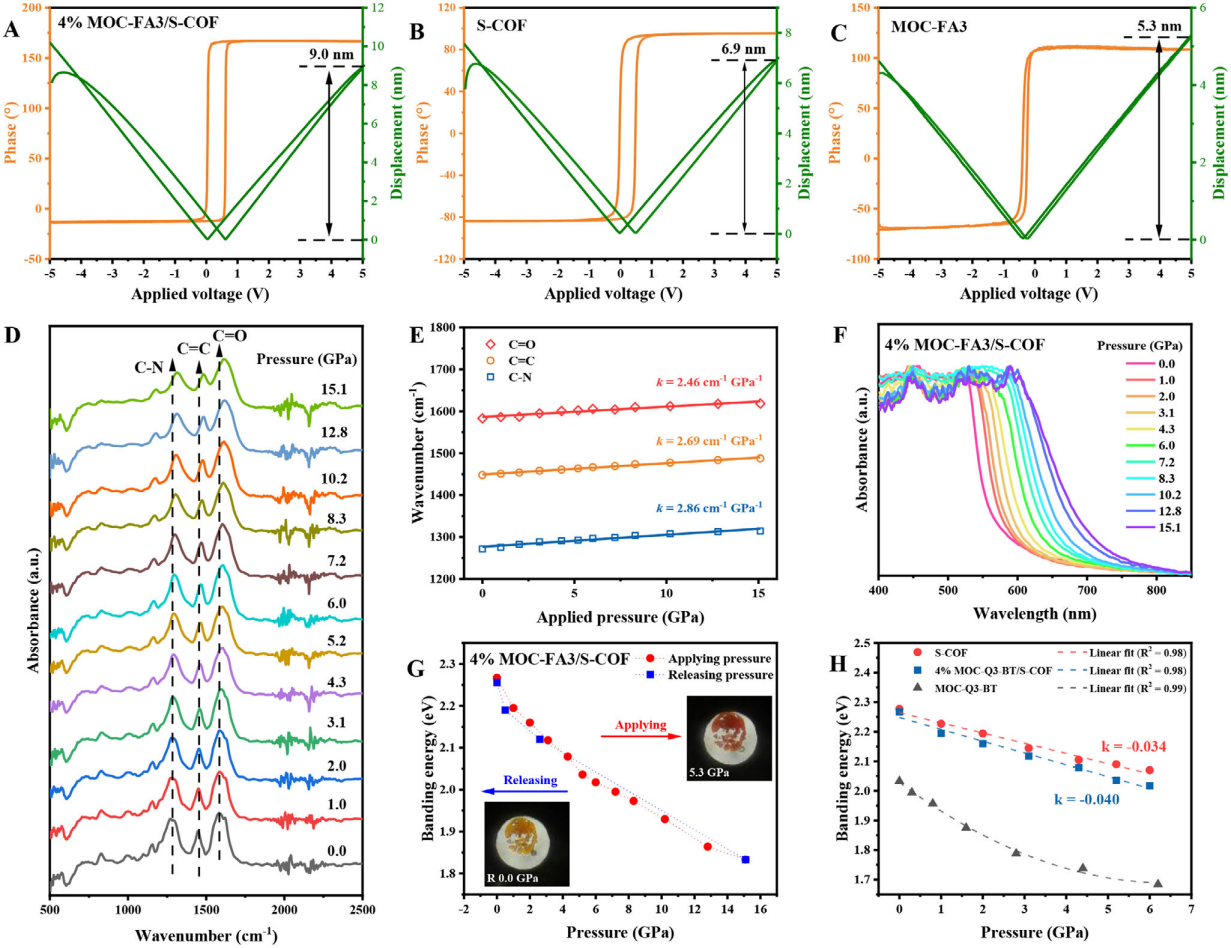
Piezoelectric response and pressure‐induced optoelectronic evolution. PFM phase hysteresis loops and amplitude butterfly loops of (A) 4% MOC‐FA3/S‐COF, (B) S‐COF, and (C) MOC‐FA3. (D) In situ pressure‐dependent FT‐IR spectra of 4% MOC‐FA3/S‐COF and (E) characteristic peak fitting showing blueshifts. (F) In situ pressure‐evolved UV–vis absorption spectra and (G) pressure‐dependent bandgap evolution with insets showing reversible chromism for 4% MOC‐FA3/S‐COF. (H) Pressure‐dependent bandgap evolution of 4% MOC‐FA3/S‐COF, S‐COF, and MOC‐FA3.

The piezoelectric properties of MOC‐FA3, S‐COF, and MOC‐FA3/S‐COF were investigated using piezoresponse force microscopy (PFM, Figure 4A–C). The piezoelectric hysteresis loop changed by 180° under a ±5 V DC electric field and showed clear hysteresis and local switching behavior for all three samples. The amplitude loop showed that the amplitude of MOC‐FA3/S‐COF was 9.0 nm (Figure 4A), while the amplitudes of S‐COF and MOC‐FA3 were 6.9 nm (Figure 4B) and 5.3 nm (Figure 4C), respectively. Under a scanning bias voltage ranging from −5 to 5 V, the maximum effective piezoelectric coefficients were determined to be approximately 1.06, 1.38, and 1.80 nm·V^−1^ for MOC‐FA3, S‐COF, and MOC‐FA3/S‐COF, respectively. The results clearly showed that the materials' piezoelectric properties followed this order: MOC‐FA3/S‐COF > S‐COF > MOC‐FA3. Additionally, S‐COF, MOC‐FA3, and 4% MOC‐FA3/S‐COF exhibited distinct resonance peaks at 195, 44, and 63 kHz, respectively (Figure S28A–C), indicating the occurrence of voltage‐induced piezoelectric vibrations within these materials. Significant linear correlations were also observed between vibration amplitude and the applied excitation voltage (Figure S29), confirming the linear piezoelectric characteristics of these materials. The amplitude and phase hysteresis loops of these samples, which were dependent on the bias voltage, demonstrated that their polarization could be switched by an electric field. Moreover, lateral PFM amplitudes and phase images of these samples (Figures S30 and S31) were also collected.

After S‐COF and MOC‐FA3 were irradiated with 1400 and 1200 nm femtosecond laser pulses, respectively, second harmonic generation (SHG) emission signals were detected at 700 and 600 nm. This result confirms that the COF and MOC materials possess non‐centrosymmetric properties. Meanwhile, the composite material 4% MOC‐FA3/S‐COF was observed to exhibit a stronger SHG response, with twice the intensity of pure S‐COF (Figure S32).

The direct piezoelectric effect of all materials was determined using the quasi‐static method. A high piezoelectric coefficient (*d*_33_) was obtained for 4% MOC‐FA3/S‐COF (3.4 pC/N, Figure S33A), S‐COF (2.4 pC/N, Figure S33B), and MOC‐FA3 (2.2 pC/N, Figure S33C), consistent with the results obtained from PFM measurements. Notably, MOC‐FA3 demonstrated intrinsic piezoelectricity, a property previously unreported for metal–organic cages. This behavior originated from its non‐centrosymmetric crystal structure and polar organic linkers, as jointly verified by PFM, *d*_33_ measurements, and DFT calculations. When coupled with photo‐piezoelectric S‐COF, the composite achieved enhanced polarized charge generation under mechanical stress. Efficient separation of these charges significantly accelerated surface redox kinetics. These findings establish MOC‐FA3/S‐COF composites as promising candidates for piezo‐catalytic applications.

To probe structural evolution under high pressure, in situ pressure‐dependent infrared spectroscopy was performed. Both the 4% MOC‐FA3/S‐COF composite (Figure 4D) and pristine S‐COF (Figure S34A) exhibited similar spectral evolution trends across the 0–15.1 GPa pressure range. This similarity arose because the composite was predominantly S‐COF, and the minor MOC‐FA3 incorporation largely retained the S‐COF framework structure. At 15.1 GPa, characteristic vibrational modes of the β‐ketoenamine subunits exhibited substantial blueshifts: the C─N stretching vibration shifted from 1271 to 1314 cm^−1^ ($\Delta \bar{v}$ = 43 cm^−1^), the C═C vibration from 1447 to 1487 cm^−1^ ($\Delta \bar{v}$ = 40 cm^−1^), and the C═O vibration from 1583 to 1618 cm^−1^ ($\Delta \bar{v}$ = 35 cm^−1^) (Figure 4E). Quantitative analysis revealed blueshift rates of 2.86, 2.69, and 4.10 cm^−1^ GPa^−1^ for the C─N, C═C, and C═O vibrations in S‐COF, respectively (Figure S34B). The significant high‐wavenumber shifts of these IR absorption peaks indicated bond shortening and increased vibrational frequencies. These observations confirmed that the distinctive keto‐enamine and TFG units constituted the primary structural origin of S‐COF's piezoelectric properties. Additionally, under increasing pressure, the stretching vibration peaks associated with the C─N bond in triphenylamine and the skeletal vibrations of the thiophene, benzothiadiazole, and pyridine units in MOC‐FA3 exhibited gradual blueshifts: At approximately 15 Gpa from 1189 to 1222 cm^−1^ ($\Delta \bar{v}$ = 33 cm^−1^), 1321 to 1390 cm^−1^ ($\Delta \bar{v}$ = 69 cm^−1^), 1477 to 1509 cm^−1^ ($\Delta \bar{v}$ = 32 cm^−1^), and 1596 to 1644 cm^−1^ ($\Delta \bar{v}$ = 48 cm^−1^), respectively (Figure S35A). Quantitative analysis revealed corresponding blueshift rates of 2.10, 4.03, 2.25, and 3.25 cm^−1^ GPa^−1^ for these vibrational modes (Figure S35B). Our findings demonstrated that the piezoelectric response in MOC‐FA3 originated primarily from the thiophene, benzothiadiazole, and pyridine moieties.

First‐principles DFT calculations (VASP 6.3.0) [47, 48] further validated the piezoelectricity of MOC‐FA3 and S‐COF through ion‐clamped piezoelectric tensor computations, revealing dominant elements of 0.91 C/m^2^ (XXX/XYY/YXY) for S‐COF and 0.25 C/m^2^ (XYY/YZZ/ZZZ/ZYZ) for MOC‐FA3 (Tables S11, S12, Figures S36 and S37), consistent with PFM and *d*_33_ measurements. Born effective charge analysis identified high‐charge ions (>3.0 |e|) localized at keto‐enamine units (Figure S38), corroborated by in situ high‐pressure FT‐IR spectral shifts, thereby confirming the synergistic origin of piezoelectric responses from polarized frameworks and establishing a complete theoretical‐experimental validation loop.

In piezoelectric materials, the piezoelectric potential generates an internal electric field that modulates the distribution of charge carriers and induces band bending, thereby enhancing the redox activity of the material. To probe pressure‐induced bandgap modulation, in situ pressure‐dependent UV/Vis absorption spectroscopy was performed. As shown in Figure 4F, the absorption edge of 4% MOC‐FA3/S‐COF exhibited a gradual redshift under applied pressure (0–15.1 GPa), corresponding to a bandgap reduction from 2.26 to 1.83 eV (Δ*E*_g_ = 0.43 eV). The bandgap nearly reverted to its initial value upon pressure release (Figure 4G), consistent with reversible color transitions observed visually: from yellow to red under pressure (Figure S39) and red to yellow after release (Figure S40). Under identical conditions, pristine S‐COF showed a bandgap decrease of 0.38 eV (2.28 → 1.90 eV, Figure S41) with analogous reversible color changes (Figures S42 and S43). Quantitative analysis revealed bandgap pressure sensitivities (d*E*_g_/d*P*) of ‐0.040 eV GPa^−1^ for 4% MOC‐FA3/S‐COF and ‐0.034 eV GPa^−1^ for S‐COF (Figure 4H). This comparison demonstrated that MOC‐FA3 incorporation enhanced the COF's pressure sensitivity and amplified its bandgap reduction. Notably, MOC‐FA3 itself exhibited a striking bandgap decreased from 2.03 to 1.27 eV (Δ*E*_g_ = 0.76 eV, Figure S44), accompanied by reversible color transitions from orange to black under pressure (Figure S45) and back to orange upon release (Figure S46). Linear fitting confirmed the exceptional pressure responsiveness of MOC‐FA3, displaying the steepest d*E*_g_/d*P* slope among all samples (Figure 4H). This endowed MOC‐FA3 with enhanced light‐harvesting capacity and charge transfer efficiency, advancing its suitability for piezo‐photocatalysis. Critically, however, the synergistic integration of MOC‐FA3 within the S‐COF matrix yielded optimal bandgap engineering and superior piezo‐photocatalytic performance.

### 2.4 Piezo‐Photocatalytic H_2_ and H_2_O_2_ Evolution

A series of systematic tests were designed to evaluate the piezo‐photocatalytic performance of MOC‐FA3, S‐COF, and 4% MOC‐FA3/S‐COF for H_2_ and H_2_O_2_ evolution from pure water splitting. The performance of several regulated experiments included photocatalytic, piezo‐catalytic, and piezo‐photocatalytic methods. These were performed under the following conditions: optical irradiation (λ ≥ 420 nm), acoustic oscillation (60 W, 40 kHz), and simultaneous optical and acoustic co‐irradiation. In an inert argon environment, water and catalysts are the only substances used, with no sacrificial reagents or noble metal co‐catalysts.

Under visible light irradiation conditions, S‐COF and 4% MOC‐FA3/S‐COF could only produce a small amount of H_2_O_2_ (7.6 and 17.1 µmol g^−1^ h^−1^). This could be because the S‐COF reduced the oxygen in the water to H_2_O_2_, as other studies have shown [49, 50]. MOC‐FA3 did not exhibit H_2_ and H_2_O_2_ evolution due to the rapid recombination of photogenerated charge carriers. This result indicated that light irradiation alone was insufficient to drive efficient water splitting reactions.

**FIGURE 5:**
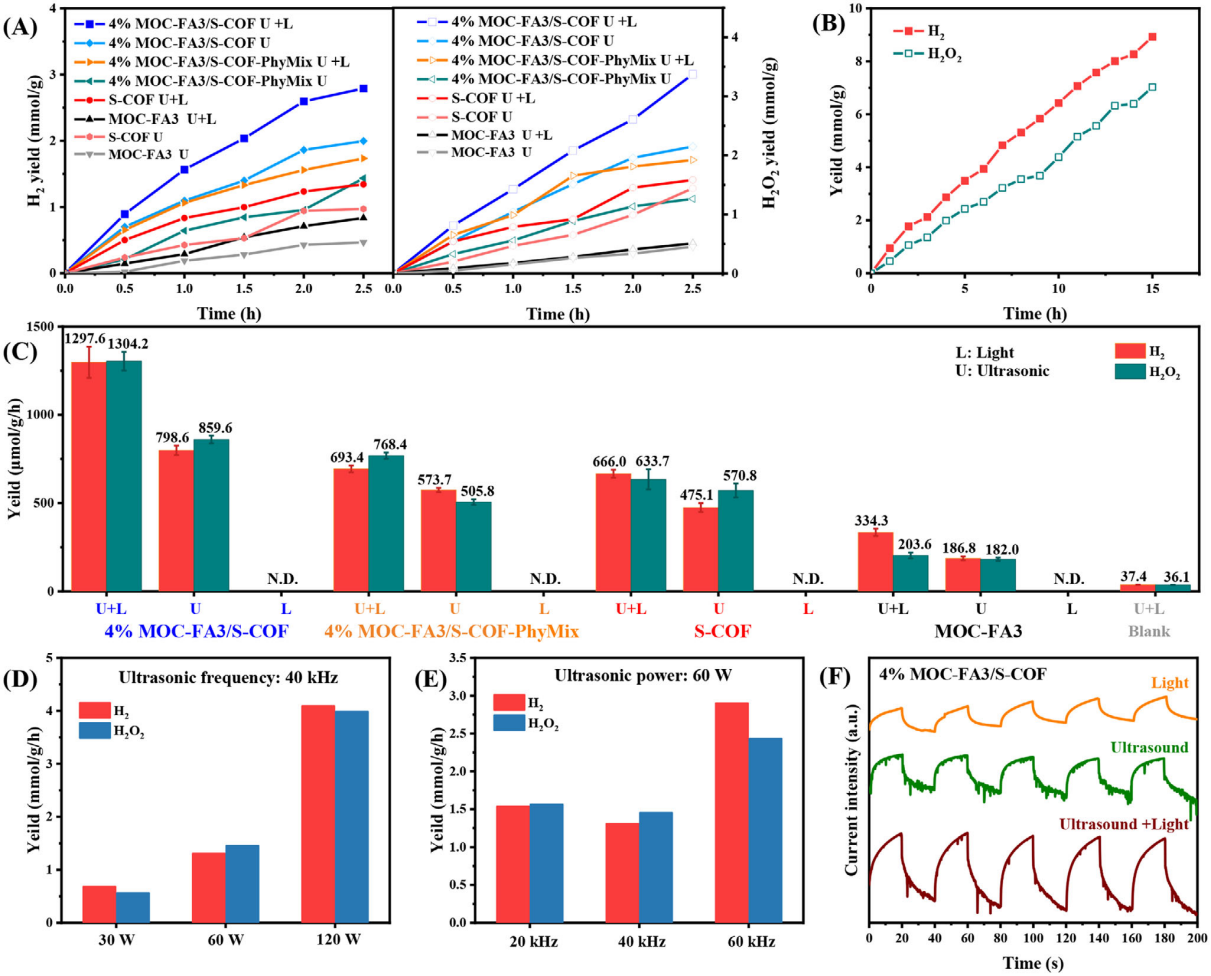
Piezo‐photocatalytic water splitting performance. (A) H_2_/H_2_O_2_ production rates under ultrasound (40 kHz, 60 W) vs. dual light‐ultrasound stimulation for MOC‐FA3, S‐COF, 4% MOC‐FA3/S‐COF‐PhyMix, and 4% MOC‐FA3/S‐COF. (B) Time‐dependent H_2_/H_2_O_2_ evolution under co‐stimulation (AM 1.5G + 40 kHz ultrasound, 60 W) for 4% MOC‐FA3/S‐COF (10 mg, 15 h). (C) Comparative H_2_/H_2_O_2_ yields across materials under optimal conditions. (D) Ultrasonic power effects (30–120 W, 40 kHz) with light irradiation (0.5 h). –) (E) Frequency‐dependent piezocatalytic rates (20–60 kHz, 60 W) with light irradiation (0.5 h). Ultrasonic power effects (30–120 W, 40 kHz) with light irradiation (0.5 h). (F) Photocurrent responses to individual/combined stimuli (dark, light, ultrasound, light+ultrasound).

When ultrasonic irradiation was applied, H_2_/H_2_O_2_ production occurred on the three samples. As shown in Figure 5A, the H_2_/H_2_O_2_ evolution rate of MOC‐FA3, S‐COF, and 4% MOC‐FA3/S‐COF was 186.8/182.0, 475.1/570.8, and 798.6/859.6 µmol g^−1^ h^−1^, respectively. Under pure ultrasound irradiation, all samples exhibited H_2_/H_2_O_2_ yields approaching a 1:1 ratio. The evidence indicated that the oxidation product of H_2_O was H_2_O_2_, thereby demonstrating that the piezoelectric potential‐induced positive charge (q^+^) oxidized OH^−^ to H_2_O_2_ rather than O_2_. Interestingly, MOC‐FA3, which had intrinsic piezoelectric properties, exhibited a moderate H_2_/H_2_O_2_ evolution performance, suggesting that it could be a promising candidate piezoelectric material for energy conversion. Compared to MOC‐FA3 and S‐COF, the higher H_2_/H_2_O_2_ production of the composites could be due to their greater piezoelectricity and more efficient vibration energy harvesting, verifying that the piezoelectric MOC‐FA3 cooperative integration with COFs could enhance the piezocatalytic performance. Compared with the 4% MOC‐FA3/S‐COF composite, the sample prepared by physical mixing (4% MOC‐FA3/S‐COF‐PhyMix) showed significantly lower piezo‐photocatalytic activity. This outcome indicated that the superior performance of the heterojunction relied on the supramolecular interactions formed between MOC‐FA3 and S‐COF, which only occurred when the two components were allowed to adsorb and recombine in a dissolved state.

Subsequently, introducing visible light into the ultrasound reaction system further enhanced the evolution of H_2_ and H_2_O_2_ for all samples (Figure 5C). Under combined ultrasonic and light conditions, 4% MOC‐FA3/S‐COF exhibited the greatest piezo‐photocatalytic performance, achieving H_2_/H_2_O_2_ generation rates of 1297.6 and 1304.2 µmol g^−1^ h^−1^, respectively. To our knowledge, this H_2_/H_2_O_2_ production rates in pure water rank among the highest piezocatalytic performance at comparable power densities under both ultrasonic and photo‐piezocatalytic conditions, even outperforming most sacrificial‐agent‐dependent inorganic piezocatalysts (Tables S12, S13, Figure S47). S‐COF showed moderate performance with H_2_/H_2_O_2_ generation rates of 666.0 and 633.7 µmol g^−1^ h^−1^, respectively. MOC‐FA3 also demonstrated piezo‐photocatalytic activity with H_2_/H_2_O_2_ generation rates of 334.3 and 203.6 µmol g^−1^ h^−1^. The improved piezo‐photocatalytic performance indicated that illumination played a significant role in promoting piezo‐catalytic activity. Due to the Z‐scheme heterojunction effect, the incorporation of MOC‐FA3 into S‐COF to obtain MOC‐FA3/S‐COF significantly enhanced the piezo‐photocatalytic overall water splitting activity. Importantly, this is the first report on the piezo‐photocatalytic performance of MOC, which presents a positive outlook on the modulation of the photo‐charge transfer process via the combined effects of ferroelectric polarization and the Z‐scheme heterojunction of MOC and COF.

A long‐term experiment was also performed on 4% MOC‐FA3/S‐COF. Within 15 h, the composite sample demonstrated H_2_/H_2_O_2_ evolution rates of 8.9 and 7.0 mmol g^−1^, respectively. The H_2_/H_2_O_2_ yield increased approximately linearly over time and remained stable for 15 h, indicating excellent piezo‐photocatalytic stability of the composite (Figure 5B). Additionally, the solid‐state UV–vis absorption spectra (Figure S48A), FT‐IR spectra (Figure S48B), XPS spectra (Figure S48C), SEM images (Figure S49A), and TEM images (Figure S49B) of both samples were measured. No significant changes were observed in these spectra, suggesting that MOC‐FA3/S‐COF retained its original molecular structure after photocatalysis.

To probe ultrasonic power effects on piezo‐photocatalysis, 4% MOC‐FA3/S‐COF was evaluated using a customized ultrasonic generator (30–120 W). H_2_ and H_2_O_2_ production rates increased progressively with power (Figure 5D), reaching 4090 and 3985 µmol g^−1^ h^−1^ at 120 W, representing 2.7‐fold enhancements over 60 W performance. This power‐dependent activity arose from intensified piezoelectric charge generation at higher ultrasonic amplitudes. Catalytic performance dependence on ultrasonic frequency was subsequently investigated. For 4% MOC‐FA3/S‐COF, H_2_/H_2_O_2_ production peaked at 60 kHz vs. 40 kHz (Figure 5E). In contrast, pristine S‐COF and MOC‐FA3 exhibited maximum activity at 40 kHz (Figure S50A) and 20 kHz (Figure S50B), respectively.

To further investigate the superior performance of MOC‐FA3/S‐COF in photo‐piezoelectric catalysis, the photocurrent responses of S‐COF, MOC‐FA3, and MOC‐FA3/S‐COF were systematically tested under illumination alone, ultrasound alone, and simultaneous illumination and ultrasound (Figure 5F; Figures S51 and S52). The preparation process of the working electrode for piezo‐photoelectrochemical testing can be found in Figure S53. The results showed that the photocurrent intensity of all three samples was significantly higher when exposed to both light and ultrasound than when exposed to light or ultrasound alone. This suggested a strong synergistic effect between light and ultrasound, which enhanced the materials' photoelectric performance. Of the three samples, the 4% MOC‐FA3/S‐COF composite had a significantly higher photocurrent intensity and superior electrochemical properties than the single‐component materials (S‐COF and MOC‐FA3). To evaluate the charge carrier dynamics during piezoelectric‐photocatalysis, EIS measurements were performed under pure ultrasound, pure light, and combined ultrasound‐light conditions (Figure S54, Table S14). Notably, all materials exhibited a significant reduction in charge transfer resistance under the combined action of light and ultrasound compared to pure light or pure ultrasound alone, suggesting an improved electronic interaction between piezoelectric charges and electroactive materials under these combined conditions. Among the samples, 4% MOC‐FA3/S‐COF showed the smallest charge transfer resistance (R_2_) under the combined ultrasound‐light condition, following the order: MOC‐FA3 (3175 Ω) > S‐COF (2871 Ω) > 4% MOC‐FA3/S‐COF (1463 Ω). These results suggested that the Z‐scheme construct strategy significantly enhanced the photocatalytic activity and charge separation efficiency of the material, both of which were crucial factors in its improved performance.

### 2.5 Piezo‐Photocatalytic Mechanism

**FIGURE 6:**
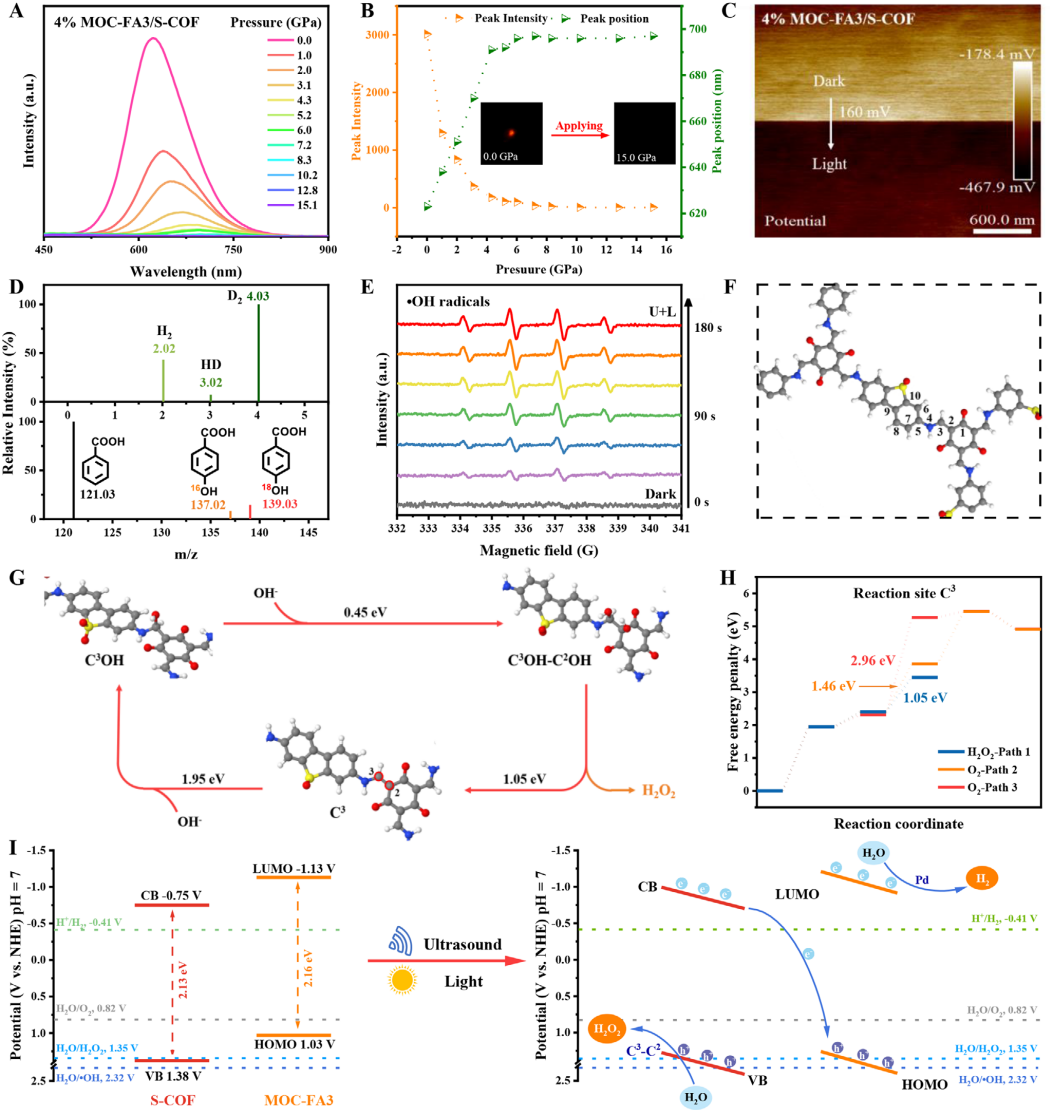
Mechanistic insights into piezo‐photocatalytic water splitting over 4% MOC‐FA3/S‐COF. (A) In situ pressure‐dependent photoluminescence (PL) spectra. (B) Pressure‐dependent PL peak position (green line), PL peak intensity (orange line), and corresponding PL images. (C) KPFM surface potential images upon light exposure after dark adaptation. (D) Mass spectra from GC‐MS analysis showing D_2_ and •^18^OH production from D_2_O and H_2_^18^O, respectively. (E) In situ EPR spectra. (F) Structure of S‐COF highlighting adsorption sites. (G) Proposed mechanism for H_2_O_2_ production on S‐COF. (H) Calculated free energy diagrams for the oxygen evolution reaction (OER) and H_2_O_2_ evolution pathways, along with subsequent reaction possibilities. (I) Schematic illustration of the proposed mechanism for overall water splitting under piezo‐photocatalysis.

In situ pressure‐dependent photoluminescence (PL) spectroscopy revealed distinct charge carrier recombination dynamics across the three samples. Under applied pressure (0–15.1 GPa), 4% MOC‐FA3/S‐COF exhibited progressive fluorescence attenuation with concurrent peak redshift (Figure 6A,B), visually manifested as a transition from red emission to near‐quenching (Figures S55 and S56). A similar behavior was observed in pristine S‐COF and MOC‐FA3 with analogous color shifts (Figures S57–S62). Mechanistically, pressure‐enhanced *π–π* interactions drove emission quenching and spectral redshifting, while simultaneously accelerating charge carrier separation/transfer to elevate electron‐hole pair density. This increased carrier accessibility enhanced redox reaction kinetics. Crucially, 4% MOC‐FA3/S‐COF demonstrated accelerated fluorescence quenching kinetics vs. pristine S‐COF, reaching minimal intensity at lower pressures (Figure S63), directly evidencing that MOC‐FA3 incorporation amplified piezoresponsiveness. Consequently, the composite achieved superior charge utilization efficiency under piezo‐photocatalytic conditions, highlighting its promise for advanced energy conversion systems.

Surface morphologies and contact potential difference (CPD) variations of the three samples were characterized by Kelvin probe force microscopy (KPFM) under dark and illuminated conditions. All samples possessed smooth surfaces with homogeneous polarization distributions under dark conditions (Figure 6C; Figures S64–S66). Upon light irradiation, significant CPD shifts occurred due to photogenerated charge separation driven by built‐in electric fields, with 4% MOC‐FA3/S‐COF exhibiting a pronounced shift of +160 mV, which was 2.1‐fold and 4.8‐fold higher than those of pristine S‐COF (+77 mV) and MOC‐FA3 (+33 mV), respectively (Figures S64–S66). This ΔCPD magnitude directly correlated with the degree of polarization field modulation by photogenerated charges: enhanced polarization field reinforcement suppressed charge recombination and amplified photo‐piezocatalytic activity. The superior CPD response of the composite unequivocally demonstrated optimized photoresponsiveness and charge separation efficiency, rationalizing its exceptional photocatalytic performance.

Charge‐trapping experiments with specific scavengers (AgNO_3_, CH_3_OH, tert‐butanol; Figure S67) definitively identified H_2_/H_2_O_2_ as reduction/oxidation products: (1) AgNO_3_ suppressed > 80% H_2_ evolution, confirming electrons reduced H⁺ to H_2_ via 2e^−^ transfer; (2) CH_3_OH enhanced H_2_ yield ∼3.5‐fold but decreased H_2_O_2_ yield ∼97%, proving holes drove H_2_O_2_ formation; (3) Tert‐butanol eliminated H_2_O_2_ production, verifying •OH as key intermediates. Thus, the piezo‐potential enabled electron‐initiated H_2_ generation and hole‐mediated H_2_O_2_ synthesis via •OH, achieving full charge utilization [51].

Isotope labeling experiments were conducted to trace the hydrogen and oxygen sources. Using D_2_O (95%) in the reduction half‐reaction generated D_2_ (m/z = 4, Figure 6D), confirming H_2_ derived from water protons, while H_2_^18^O (98%) oxidation produced trapped •^18^OH intermediates identified as p‐hydroxybenzoic‐^18^OH (m/z = 139.03) and its natural counterpart p‐hydroxybenzoic‐^16^OH (m/z = 137.02). Control experiments without H_2_^18^O showed only natural isotopic signatures of p‐hydroxybenzoic acid (m/z = 137.02, 138.97), verifying that H_2_O_2_ oxygen originated exclusively from water (Figure 6D; Figure S68).

In situ electron paramagnetic resonance (EPR) spectroscopy with 5,5‐dimethyl‐1‐pyrroline N‐oxide (DMPO) as spin trap was employed to probe •OH generation pathways from H_2_O_2_. Reaction intermediates were dynamically monitored via a closed‐loop system: aqueous samples were continuously circulated from the quartz reactor (undergoing simultaneous ultrasound/light irradiation) to the EPR detection cell using a peristaltic pump (experimental setup: Figure S69). Distinct EPR signals emerged within 30 s of reaction initiation, intensifying progressively and peaking at 120 s (Figure 6E), indicating progressive accumulation of radical species.

To elucidate the H_2_O_2_ formation mechanism during piezo‐photocatalysis, terephthalic acid (TA) probed •OH generation kinetics in MOC‐FA3, S‐COF, and 4% MOC‐FA3/S‐COF under concurrent ultrasound/light irradiation. Time‐dependent fluorescence spectra (Figure S70) exhibited progressive TA‐OH adduct formation (15–90 min), confirming continuous •OH accumulation. After 90 min, composite fluorescence intensity followed: 4% MOC‐FA3/S‐COF > S‐COF > MOC‐FA3 (Figure S71), with the composite showing a stronger signal than pristine S‐COF. This •OH intensity‐ H_2_O_2_ yield correlation definitively established hydroxyl radicals as pivotal intermediates. The piezoelectric systems (MOC‐FA3, S‐COF, and MOC‐FA3/S‐COF composites) efficiently mediated the indirect two‐electron water oxidation reaction (WOC, H_2_O + h^+^ → •OH + H^+^, 2.32 V vs. NHE; 2•OH → H_2_O_2_, 0.74 V vs. NHE).

Complementary DFT calculations elucidated the piezo‐photocatalytic water‐splitting mechanisms in S‐COF. Hydrogen evolution (HER) localized at cathodic sites near CB regions (Figure S72), with C^7^ (SA unit) exhibiting optimal activity through minimal H^+^ adsorption energy (Δ*G* = 1.09 eV, Table S15, Figure S73). Conversely, oxidation reactions occurred near VB domains (Figure S74), where the preferential H_2_O_2_ formation pathway initiated via OH^−^ adsorption at C^3^ (Δ*G* = 1.95 eV, Table S16, Figure S75), followed by adjacent adsorption at C^2^ (Δ*G* = 0.45 eV), culminating in H_2_O_2_ release (Δ*G* = −1.05 eV; cumulative barrier: 1.05 eV, Figure 6G). Competitive O_2_ pathways required substantially higher energies (Figure 6H): Path 2 involved C^3^OH deprotonation (Δ*G* = 1.46 eV) and O‐atom coupling (Δ*G* = 1.59 eV), while Path 3 proceeded through C^3^OOH formation (Δ*G* = 2.96 eV) and O─O bond formation (Δ*G* = 0.19 eV), both accumulating barriers > 3.05 eV (Figure S76). The significant energy advantage of the H_2_O_2_ pathway (1.05 eV vs. > 3.05 eV) rationalized exclusive peroxide formation under piezo‐photocatalytic conditions.

DFT calculations identified the oxidation reaction sites in MOC‐FA3, revealing 13 potential OH^−^ adsorption sites (C^1^‐C^13^, Figure S77). Among these, C^3^, C^11^, and C^13^ exhibited the lowest adsorption energies (E_a_d_s_, Table S17, Figure S78). The optimal H_2_O_2_ formation pathway initiated at the thiophene‐derived C^11^ site (E_a_d_s_ = 1.48 eV), proceeding via adjacent C^12^ adsorption (Δ*G* = 1.03 eV) to H_2_O_2_ formation/release (Δ*G* = 0.94 eV; cumulative barrier: 1.05 eV, Figure S79A). Alternative O_2_ pathways required substantially higher energy inputs. Path 2 demanded sequential deprotonation (Δ*G* = 1.19 eV), O‐adatom formation (Δ*G* = 2.20 eV), and O_2_ release (Δ*G* = ‐0.06 eV; Σ = 3.33 eV, Figure S79B). The thermodynamic preference for the C^11^‐C^12^ pathway (1.05 eV vs. > 3.33 eV) confirmed exclusive H_2_O_2_ production (Figure S80), complementing the established HER activity at Pd sites.

Experimental and computational evidence supported a Z‐scheme piezo‐photocatalytic mechanism in MOC‐FA3/S‐COF (Figure 6I): Upon concurrent light/ultrasound excitation, strain‐induced piezoelectric fields from structural deformation modulated band structures and enhanced charge separation. Critically, photoexcited electrons in S‐COF (VB→CB) transferred to MOC‐FA3's HOMO, while holes remained in S‐COF's VB to oxidize OH^−^ at C^3^ sites via •OH intermediates for H_2_O_2_ production. Simultaneously, MOC‐FA3's photoexcited electrons (HOMO→LUMO) drived Pd‐catalyzed H_2_ evolution. This strain‐enhanced Z‐scheme synergistically completed the water‐splitting cycle.

## 3 Conclusion

In summary, this work pioneers a dual‐functional energy conversion platform through rational design of a Z‐scheme heterojunction comprising a photo‐piezoelectric Pd_3_L_2_ metal–organic cage (MOC‐FA3) and a β‐ketoenamine‐linked COF (S‐COF). Supramolecular integration via hydrogen bonding and *π–π* stacking yielded exceptional photocatalytic H_2_ evolution under ascorbate conditions (1266 mmol·g^−1^, TON_Pd_ = 180,842), attributed to extended visible‐light absorption, enhanced charge separation, and atomically dispersed Pd sites. Significantly, we demonstrate the intrinsic piezoelectricity of MOCs, as evidenced by KPFM, piezoelectric coefficient measurements, in situ high‐pressure spectroscopy, and piezophotocatalytic performance yielding H_2_ and H_2_O_2_ production rates of 334.3 and 203.6 µmol g^−1^ h^−1^, respectively. The heterojunction achieved cooperative piezophotocatalytic overall water splitting under ultrasonic irradiation (40 kHz, 60 W) and AM 1.5G illumination, generating H_2_ and H_2_O_2_ at 1297.6 and 1304.2 µmol g^−1^ h^−1^ respectively, 1.95 and 2.06‐fold enhancements over pristine S‐COF. In situ spectroscopy (EPR, FT‐IR, PL, UV–vis) and DFT calculations revealed the water‐splitting mechanism, identifying Pd (H_2_ evolution) and C^2^‐C^3^ (H_2_O_2_ formation) as potential active centers. This study establishes two paradigm shifts: MOCs as a new piezoelectric materials class, overturning symmetry‐based design conventions, and synergistic Z‐scheme/piezoelectric coupling for mechano‐photonic energy conversion.

## 4 Experimental Methods

### 4.1 Synthesis of MOC‐FA3

A‐3 (22.50 mg, 0.02 mmol) and tetramethylethylenediamine palladium nitrate (Pd(tmeda)(NO_3_)_2_, 10.50 mg, 0.03 mmol) were dissolved in DMSO (1.5 mL), and then stirred at 60°C for 3 h. Ethyl acetate was used to precipitate the reactant, and the precipitate was filtered and then dried under vacuum at 60°C to give 24.50 mg of dark brown solid MOC‐FA3. Yield: 75%. ^1^H NMR (400 MHz, DMSO‐*d*_6_) δ (ppm): 9.80 (s, 6H), 9.04 (d, *J* = 4.0 Hz, 6H), 8.47 (d, *J* = 5.5 Hz, 6H), 8.12 (d, *J* = 6.6 Hz, 6H), 8.05‐7.84 (m, 24H), 7.74 (d, *J* = 2.0 Hz, 6H), 7.64‐7.48 (m, 6H), 7.25 (d, *J* = 12.4 Hz, 12H), 3.08 (s, 12H), 2.76‐2.69 (m, 36H). ESI‐MS (m/z): [C_144_H_120_N_30_O_12_Pd_3_S_12_]^2+^ Calculated: 1583.1727, found: 1583.1747.

### 4.2 Synthesis of S‐COF

A Pyrex tube (diameter: 6.0 mm) was filled with 2,4,6‐trihydroxy‐1,3,5‐benzenetricarbaldehyde (10.50 mg, 0.05 mmol), 5,5‐dioxodibenzothiophene‐3,7‐diamine (18.45 mg, 0.075 mmol), 0.15 mL glacial acetic acid, 0.15 mL water, mesitylene (1.50 mL), and 1,4‐dioxane (1.50 mL). The mixture was homogenized by sonication for 5 min, and then the tube was flash frozen at 77.3 K (liquid N_2_ bath) and degassed by 2 freeze‐pump‐thaw cycles. The precipitate was collected by centrifugation and washed with N, N‐dimethylformamide and tetrahydrofuran. After drying at 60°C under vacuum overnight, the product was obtained as a red powder with a yield of 85%.

### 4.3 Synthesis of MOC‐FA3/S‐COF

S‐COF (5 mg) was dispersed in DMSO (5 mL) and mixed with MOC‐FA3 in an appropriate amount of DMSO solutions with calculated contents (3/4/5 wt.%). The mixture was sonicated for 5 min and stirred at 25°C for 2 h. The precipitate was collected by centrifugation and washed with tetrahydrofuran. The solid MOC‐FA3/S‐COF was obtained by vacuum drying and designated as 3/4/5% MOC‐FA3 /S‐COF.

### 4.4 Synthesis of Comparative Samples

A‐3/S‐COF, Pd/S‐COF, and A‐3/Pd/S‐COF were prepared by following the aforementioned synthesis procedure of MOC‐FA3/S‐COF (Pd(tmeda)(NO_3_)_2_ as the Pd source). The contents of Pd and A‐3 in the above‐mentioned samples were tailored to match those of 4% MOC‐FA3/S‐COF for comparative studies. 4% MOC‐FA3/S‐COF‐PhyMix was obtained by physically mixing MOC‐FA3 (4%) with S‐COF in a mortar.

### 4.5 Photocatalytic Hydrogen Production under Xe Lamp

Photocatalytic experiments were conducted on an all‐glass automated online trace gas analysis system (Labsolar‐6A, Beijing Perfectlight Technology Co., Ltd.), which was coupled to a FULI GC9790Plus gas chromatograph. A total of 2 mg of the photocatalyst was dispersed in 60 mL of 0.1 m aqueous ascorbic acid solution, and the mixture was placed in a quartz reaction cell integrated with the aforementioned online system. After eliminating dissolved air from the photocatalytic system, the setup was degassed and subjected to irradiation under vacuum conditions using a 300 W Xe lamp (Perfect Light PLS‐SXE300, light intensity = 1000 mW cm^−2^) fitted with an optical filter (λ ≥ 420 nm). The volume of hydrogen produced was quantified via online gas chromatography at 1 h intervals.

### 4.6 Piezo‐Photocatalytic H_2_ and H_2_O_2_ Evolution

The suspension was irradiated with a 300 W Xe lamp (Perfect Light PLS‐SXE300) equipped with a visible light cut‐off filter (λ ≥ 420 nm) and sonicated using a JP101T single‐frequency ultrasonic cleaner (40 kHz, 60 W, Shenzhen Jiemen Ultrasonic Instrument Co., Ltd.). System temperature was maintained at ambient level via a circulating cooling water setup. Specifically, 2 mg of the synthesized photocatalyst was dispersed in 20 mL of ultrapure water, and the resultant mixture was transferred into a 40 mL cylindrical photoreactor (27.5 mm outer diameter, 95 mm height). Prior to light exposure, the reactor system was subjected to a three‐cycle degassing process: each cycle involved 5 min of vacuum evacuation followed by 5 min of argon gas purging, which effectively eliminated dissolved oxygen and guaranteed strict anaerobic reaction conditions. The concentration of generated H_2_ was determined by gas chromatography (FULI GC9790Plus) configured with a thermal conductivity detector (TCD). For the quantification of H_2_O_2_, a TMB‐H_2_O_2_‐HRP enzymatic assay was employed (refer to the H_2_O_2_ Measurement section in the Supporting Information), where horseradish peroxidase (HRP) acted as the catalyst to facilitate the chromogenic reaction between H_2_O_2_ and 3,3',5,5'‐tetramethylbenzidine (TMB).

### 4.7 In Situ High‐Pressure PL, IR, and UV–vis Absorption Spectra

High‐pressure experiments were carried out using a symmetric diamond anvil cell (DAC) equipped with ultralow fluorescence diamond anvils featuring a 400 µm culet diameter. The samples were loaded into a 150 µm‐diameter cavity, which was fabricated via laser drilling on a pre‐indented T301 stainless steel gasket with a thickness of 45 µm. Silicon oil (PL/UV–vis grade) or potassium bromide (KBr, for IR tests) was selected as the pressure‐transmitting medium, while in situ pressure calibration was implemented based on the fluorescence shift of the R_1_ peak. All the measurements were performed at a constant temperature of 298 K. The spectroscopic characterizations included three aspects: (1) Photoluminescence (PL) spectroscopy: The samples were excited by a 355 nm semiconductor laser, whose power was stabilized to avoid intensity‐related artifacts, and the resultant PL spectra were collected using an Ocean Optics QE65000 fiber‐optic spectrometer. (2) UV–vis absorption spectroscopy: A deuterium‐halogen hybrid light source was connected to the aforementioned spectrometer for excitation, with the detection focused on the excitonic absorption bands. (3) Infrared (IR) spectroscopy: The measurements were conducted on a Nicolet iN10 microscopic spectrometer equipped with a liquid nitrogen‐cooled MCT detector, and KBr was adopted as the pressure‐transmitting medium in this case. Ruby served as the most extensively utilized pressure calibration material in contemporary high‐pressure experimental studies. Upon laser irradiation, ruby generated two adjacent and relatively strong fluorescence peaks, namely R_1_ and R_2_ (Figure S79). Among them, the R_1_ peak at lower wavenumbers showed higher emission intensity than the R_2_ peak at higher wavenumbers. Notably, both fluorescence peaks underwent a linear redshift with the change of applied pressure. The correlation between the R_1_ peak position shift and pressure could be described by the following Equation (1):

(1) $$P=\frac{1904}{7.665}\left[{\left(\frac{\Delta \lambda }{\lambda }+1\right)}^{7.665}-1\right]$$

Herein, λ denoted the R_1_ peak position under ambient pressure conditions, while Δλ represented the shift magnitude of the R_1_ peak position under different pressure loads. Thus, the pressure inside the sample chamber could be directly derived by measuring the R_1_ fluorescence peak position of the ruby. Furthermore, to verify the stability of the system at each preset pressure point, multiple rounds of spectral collection and comparative analysis were conducted. No additional spectral shifts or intensity variations were observed throughout the measurement cycle, which confirmed that the sample maintained good stability under the applied pressure during the entire data acquisition process.

## Funding

The National Natural Science Foundation Project of China (grant No. 21975291 and 22275214), Natural Science Foundation Project of Guangdong Province (grant No. 2022A1515011949 and 2024A1515012076), and “Climbing Program” Special Funds for the Cultivation of Guangdong College Students' Scientific and Technological Innovation (grant No. pdjh2024b014). [Correction added on 15 May 2026 after first online publication: grant number of Natural Science Foundation of China and Guangdong Province are updated in this version.]

## Conflicts of Interest

The authors declare no conflicts of interest.

## Supporting information




**Supporting File**: advs73646‐sup‐0001‐SuppMat.docx.

## Data Availability

The data that support the findings of this study are available from the corresponding author upon reasonable request.
