# Supplementary material for: A Dual‐Piezoelectric Metal–Organic Cage/COF Z‐Scheme Heterojunction for Solar‐Mechanical Water Splitting
Source: Adv Sci (Weinh). 2026 Jan 4;13(10):e23030. doi: 10.1002/advs.202523030 (PMC12915073; doi:10.1002/advs.202523030)
Supplement: Supplementary file 1 — Supporting File: advs73646‐sup‐0001‐SuppMat.docx. [file ADVS-13-e23030-s001.docx]

Supporting Information

A Dual-Piezoelectric Metal-Organic Cage/COF Z-Scheme Heterojunction for Solar-Mechanical Water Splitting

Xin-Ao Li, Yixuan Wang, Zi-Zhan Liang, Xin-Bang Peng, Li-Min Xiao, Li Gong, Xinyi Yang^*^, Bo Zou, Yecheng Zhou, Jun-Min Liu^*^

Xin-Ao Li, Zi-Zhan Liang, Xin-Bang Peng, Yecheng Zhou, Jun-Min Liu

The Key Laboratory of Low-Carbon Chemistry & Energy Conservation of Guangdong Province, School of Materials Science and Engineering, Sun Yat-sen University, Guangzhou 510006, China.

E-mail: liujunm@mail.sysu.edu.cn

Yixuan Wang, Xinyi Yang, Bo Zou

Synergetic Extreme Condition High-Pressure Science Center, State Key Laboratory of Superhard Materials, College of Physics, Jilin University, Changchun 130012, China.

E-mail: yangxinyi@jlu.edu.cn

Li-Min Xiao

School of Computer Science and Engineering, Beihang University, Beijing 100191, China.

Li Gong

Instrumental Analysis Research Center, Sun Yat-sen University, Guangzhou 510275, China.

Supplementary Experimental Details 6

**Figure S1.** Synthesis routes of the organic ligand **A-3**. 6

**Figure S2.** ^1^H NMR spectra in chloroform-*d* (400 MHz, 300 K) of (A) A-1, (B) A-2, (C) A-3, and ^1^H NMR spectra in DMSO-*d*_6_ (400 MHz, 300 K) of (D) MOC-FA3. 14

**Figure S3.** ^13^C NMR spectra in chloroform-*d* (101 MHz) of (A) A-1, (B) A-2 and (C) A-3. 15

**Figure S4.** ESI-MS analysis of (A) A-1, (B) A-2 and (C) A-3. 16

**Figure S5.** Comparative ^1^H NMR spectra in DMSO-*d*_6_ (400 MHz, 300 K) of (A) the ligand A-3 and (B) MOC-FA3. 17

**Figure S6.** ^1^H DOSY spectrum in DMSO-*d*_6_ of MOC-FA3. 18

**Figure S7.** ESI-MS analysis of MOC-FA3 showing charge states (2+ to 6+). 20

**Figure S8.** Simulated structure of MOC-FA3 showing (A) top and (B) side views. 21

**Figure S9.** ATR-IR spectra of (A) A-3/MOC-FA3 and (B) TFG/SA/S-COF; (C) ^13^C NMR spectrum and (D) chemical structure of S-COF. 22

**Figure S10.** (A) Experimental, pawley refined, and simulated PXRD patterns of S-COF; (B) Simulated PXRD patterns of S-COF with AA and AB stacking modes. 23

**Table S1.** Fractional atomic coordinates for the unit cell of S-COF. 24

**Table S2.** MOC-FA3 content in 4% MOC-FA3/S-COF quantified by ICP-MS. 25

**Figure S11.** SEM images of (A) S-COF and (B) 4% MOC-FA3/S-COF, and TEM images of (C) S-COF and (D) 4% MOC-FA3/S-COF. 26

**Figure S12.** TEM elemental mapping results of S-COF. 27

**Table S3.** Elemental contents of S-COF as determined by TEM mapping. 28

**Figure S13.** TEM elemental mapping results of 4% MOC-FA3/S-COF. 29

**Table S4.** Elemental contents of 4% MOC-FA3/S-COF as determined by TEM mapping. 30

**Figure S14.** AC-HAADF-STEM image of 4% MOC-FA3/S-COF. 31

**Figure S15.** (A) N_2_ adsorption-desorption isotherms and (B) pore size distribution curves of S-COF; (C) N_2_ adsorption-desorption isotherms and (D) pore size distribution curves of 4% MOC-FA3/S-COF. 32

**Figure S16.** ATR-IR spectra of S-COF, 4% MOC-FA3/S-COF and MOC-FA3. 33

**Figure S17.** Pd_3d_ XPS spectra of 4% MOC-FA3/S-COF and MOC-FA3 samples. 34

**Table S5.** The corresponding AQYs of 4% MOC-FA3/S-COF. 35

**Figure S18.** (A) Pd_3d_ XPS spectra, (B) PXRD patterns, (C) solid UV-Vis absorption spectra and (D) ATR-IR spectra of 4% MOC-FA3/S-COF before and after photocatalysis. 36

**Figure S19.** Cyclic voltammograms of (A) ferrocene and (B) MOC-FA3 measured in DMSO/CH_3_CN (1:1, v/v) solutions containing 0.1 M TBAPF_6_ at a scan rate of 500 mV/s; (C) UV-Vis absorption and fluorescence emission spectra of MOC-FA3 in DMSO; (D) Schematic energy level diagram of MOC-FA3. 37

**Table S6.** Experimental electrochemical parameters and corresponding frontier molecular orbital energy levels ........................................................................................................................................38

**Figure S20.** (A) Mott-Schottky plot of S-COF in 0.5 M Na_2_SO_4_ (aq); (B) Tauc plot for band gap determination; (C) Valence band (VB) XPS spectrum; (D) Schematic energy level diagram of S-COF constructed from the results of (A-C). 39

**Figure S21.** Calculated energy levels of the MOC-FA3/S-COF composite. 40

**Figure S22.** Contrasting •OH generation mechanisms: (A) Direct radical formation on S-COF vs. (B) Electron-transfer-mediated •OH production on 4% MOC-FA3/S-COF. 42

**Figure S23.** SPV spectra of S-COF and 4% MOC-FA3/S-COF. 43

**Figure S24.** Transient absorption spectra of (A) S-COF and (B) MOC-FA3/S-COF, and transient absorption traces normalized to the 530 nm exciton bands for (C) S-COF and (D) MOC-FA3/S-COF. 44

**Figure S25.** Photocurrent profiles of S-COF, MOC-FA3 and 4% MOC-FA3/S-COF. 45

**Figure S26.** EIS Nyquist plots of MOC-FA3, S-COF and 4% MOC-FA3/S-COF measured in the dark (R_1_: Series resistance; R_2_: Charge-transfer resistance; CPE: Constant phase element). 46

**Table S7.** Fitting results of ElS Nyquist plots for MOC-FA3, S-COF and 4% MOC-FA3/S-COF under dark. 46

**Figure S27.** EIS Nyquist plots of MOC-FA3, S-COF and 4% MOC-FA3/S-COF under light irradiation. 47

**Table S8.** Fitting results of ElS Nyquist plots for MOC-FA3, S-COF and 4% MOC-FA3/S-COF under light irradiation. 47

**Figure S28.** Applied voltage-dependent resonant peaks for (A) 4% MOC-FA3/S-COF, (B) S-COF, and (C) MOC-FA3. 48

**Figure S29.** Linear correlation between operating voltage and resonant peak amplitude. 49

**Figure S30.** AFM characterization of 4% MOC-FA3/S-COF: (A) 2D height topography, (B) 3D rendered topography, (C) PFM amplitude, and (D) PFM phase images. 50

**Figure S31.** AFM characterization of S-COF: (A) 2D height topography, (B) 3D rendered topography, (C) PFM amplitude, and (D) PFM phase images. 51

**Figure S32.** Overlay of emission spectra: (A) for 4% MOC-FA3/S-COF, S-COF, and reference KDP (1400 nm femtosecond laser illumination); (B) for MOC-FA3 and reference KDP (1200 nm femtosecond laser illumination). 52

**Figure S33.** Piezoelectric coefficient (*d*_33_) of (A) 4% MOC-FA3/S-COF, (B) S-COF, and (C) MOC-FA3 measured via the Berlincourt quasi-static method. 53

**Figure S34.** (A) *In situ* high-pressure FT-IR spectra and (B) peak fitting analysis of S-COF. 54

**Figure S35.** (A) *In situ* high-pressure FT-IR spectra and (B) peak fitting analysis of MOC-FA3. 55

**Table S9.** Piezoelectric tensor components of S-COF calculated by DFPT. 56

**Table S10.** Piezoelectric tensor components of MOC-FA3 calculated by DFPT. 57

**Figure S36.** Atom-resolved piezoelectric tensor distribution in S-COF calculated by DFPT. 58

**Figure S37.** Atom-resolved piezoelectric tensor distribution in MOC-FA3 calculated by DFPT. 59

**Table S11.** Born effective charge tensors of key atoms in S-COF Computed via DFPT (Unit: |e|) 60

**Figure S38.** Spatial distribution of anomalously high born effective charges ( >3.0 |e|) in S-COF. 63

**Figure S39.** *In situ* solid UV-Vis images of 4% MOC-FA3/S-COF under different pressures. 64

**Figure S40.** *In situ* solid UV-Vis images of 4% MOC-FA3/S-COF by releasing pressure. 65

**Figure S41.** *In situ* high pressure solid UV-Vis absorption spectra (A) under pressure and (B) by releasing pressure of S-COF; (C) Pressure-dependent band gap and corresponding UV-Vis images of S-COF. 66

**Figure S42.** *In situ* solid UV-Vis images of S-COF under different pressures. 67

**Figure S43.** *In situ* solid UV-Vis images of S-COF by releasing pressure. 68

**Figure S44.** *In situ* high pressure solid UV-Vis absorption spectra (A) under pressure and (B) by releasing pressure of MOC-FA3; (C) Pressure-dependent band gap and corresponding UV-Vis images of MOC-FA3. 69

**Figure S45.** *In situ* solid UV-Vis images of MOC-FA3 under different pressures. 70

**Figure S46.** *In situ* solid UV-Vis images of MOC-FA3 by releasing pressure. 71

**Table S12.** Comparative analysis of reported catalysts for piezo-photocatalytic O_2_/H_2_O_2_ production. 72

**Table S13.** Comparative analysis of reported catalysts for piezo-photocatalytic H_2_ production. 73

**Figure S47.** Comparative performance of piezo-photocatalytic systems. (A) H_2_ and H_2_O_2_/O_2_ production; (B) H_2_ production. 74

**Figure S48.** (A) Solid UV-Vis absorption spectra, (B) FT-IR spectra, and (C) XPS spectra of 4% MOC-FA3/S-COF before and after 15 h of continuous piezo-photocatalysis. 75

**Figure S49.** (A) SEM and (B) TEM images of 4% MOC-FA3/S-COF before and after 15 h of continuous piezo-photocatalysis. 76

**Figure S50.** Piezocatalytic H_2_ and H_2_O_2_ by (A) S-COF and (B) MOC-FA3 under different ultrasonication frequencies on 60 W in 0.5 h. 77

**Figure S51.** Photocurrent profiles of (A) MOC-FA3 and (B) S-COF under ultrasound, light and both ultrasound and light. 78

**Figure S52.** Photocurrent profiles of comparison of all samples under both ultrasound and light. 79

**Figure S53.** Preparation process of working electrode for piezo-photoelectrochemical testing. 80

**Figure S54.** EIS Nyquist plots of (A) MOC-FA3, (B) S-COF and (C) 4% MOC-FA3/S-COF under ligth or both ultrasound and light; (D) Comparison EIS Nyquist plots of MOC-FA3, S-COF and 4% MOC-FA3/S-COF under ultrasound and light. 81

**Table S14.** Fitting results of ElS Nyquist plots for MOC-FA3, S-COF and 4% MOC-FA3/S-COF under the synergistic effect of light irradiation (L) and ultrasound (U). 81

**Figure S55.** *In situ* PL images of 4% MOC-FA3/S-COF under different pressures. 82

**Figure S56.** *In situ* PL images of 4% MOC-FA3/S-COF by releasing pressure. 83

**Figure S57.** *In situ* high pressure PL spectra (A) under pressure and (B) by releasing pressure of S-COF; (C) Pressure-dependent PL peak position (green line), PL peak intensity (orange line) and corresponding PL images of S-COF. 84

**Figure S58.** *In situ* high pressure PL spectra (A) under pressure and (B) by releasing pressure of MOC-FA3; (C) Pressure-dependent PL peak position (green line), PL peak intensity (orange line) and corresponding PL images of MOC-FA3. 85

**Figure S59.** *In situ* PL images of S-COF under different pressures. 86

**Figure S60.** *In situ* PL images of S-COF by releasing pressure. 87

**Figure S61.** *In situ* PL images of MOC-FA3 under different pressures. 88

**Figure S62.** *In situ* PL images of MOC-FA3 by releasing pressure. 89

**Figure S63.** The variation trend of pressure-dependent fluorescence intensity of S-COF, MOC-FA3 and 4% MOC-FA3/S-COF 90

**Figure S64.** Multiscale characterization of 4% MOC-FA3/S-COF. (A) 3D topographical mapping; (B) Surface morphology analysis; (C) Light-triggered surface potential response. 91

**Figure S65.** Multiscale characterization of MOC-FA3. (A) 3D topographical mapping; (B) Surface morphology analysis; (C) Light-triggered surface potential response; (D) Corresponding potential distribution map. 92

**Figure S66.** Multiscale characterization of S-COF. (A) 3D topographical mapping; (B) Surface morphology analysis; (C) Light-triggered surface potential response; (D) Corresponding potential distribution map. 93

**Figure S67.** Radical trapping (0.5 h) investigation for piezo-photocatalytic mechanisms over 4% MOC-FA3/S-COF. 94

**Figure S68.** Isotopic verification of hydroxyl radical (•OH) origin via GC-MS by 4% MOC-FA3/S-COF. (A) H_2_O system: m/z signals for •OH adducts; (B) H_2_^18^O system: m/z shift to •^18^OH products. 95

**Figure S69.** Photographs of in-situ EPR experimental systems using a ultrasonic cleaner and Xe lamp for piezo-catalytic reaction. 96

**Figure S70.** Time-dependent liquid PL spectra of TA-OH under 315 nm excitation for (A) MOC-FA3, (B) S-COF, and (C) 4% MOC-FA3/S-COF; (D) Comparison of liquid PL intensity for the three samples at 75 min under simultaneous ultrasound and irradiation. 97

**Figure S71.** Time-dependent liquid PL intensity of TA-OH under 315 nm excitation for MOC-FA3, S-COF, and 4% MOC-FA3/S-COF under simultaneous ultrasound and irradiation. 98

**Figure S72.** Conduction band spatial distribution in S-COF. 99

**Table S15.** Site-specific adsorption free energy of H^+^ on S-COF. 100

**Figure S73.** Calculated H^+^ adsorption free energy barriers (ΔG) at specific sites on S-COF for the HER. 101

**Figure S74.** Valence band spatial distribution in S-COF. 102

**Table S16.** Site-specific adsorption free energy of OH^-^ on S-COF. 103

**Figure S75.** Calculated OH^-^ adsorption free energy barriers (ΔG) at specific sites on S-COF for the OER and H_2_O_2_ evolution reactions. 104

**Figure S76.** Proposed reaction pathways for H_2_O_2_ formation and O_2_ evolution on S-COF. 105

**Figure S77.** Structure of MOC-FA3 and identified adsorption sites. 106

**Table S17.** Site-specific adsorption free energy of OH^-^ on MOC-FA3. 107

**Figure S78.** Calculated OH^-^ adsorption free energy barriers (ΔG) at specific sites on MOC-FA3 for the OER and H_2_O_2_ evolution reactions. 108

**Figure S79.** (A) Gibbs free energy diagrams for the H_2_O_2_ evolution reaction at different C-C reaction sites on MOC-FA3; (B) Gibbs free energy diagrams for both the OER and H_2_O_2_ evolution reaction at the C^11^-C^12^ site on MOC-FA3. 109

**Figure S80.** Proposed reaction pathways on MOC-FA3: one for H_2_O_2_ formation and one for O_2_ evolution on the catalyst. 110

**Figure S81.** The fluorescence spectrum of the ruby. 110

References ....................................................................................................................................111

Supplementary Experimental Details

**Synthesis of organic ligands**

1. Synthesis routes of the organic ligand **A-3**.

**Synthesis of A-0**

Under an argon atmosphere, 4,7-dibromo-2,1,3-benzothidiazole (2.94 g, 10.00 mmol), 2-thiopheneboronic acid (1.66 g, 13.00 mmol), Pd(PPh_3_)_4_ (0.69 g, 0.60 mmol), 1,4-dioxane (Diox, 100 mL) and aqueous K_2_CO_3_ solution (20 mL, 1.60 mol/L) were added to a 250 mL round-bottomed flask. The reaction mixture was stirred at 90 ^o^C and the reaction progress was monitored by TLC. After cooling to room temperature, the mixture was rotary evaporated, extracted with CH_2_Cl_2_, and the organic phase was dried. The crude product was purified by silica gel column chromatography and recrystallized using dichloromethane and petroleum ether (DCM/PE = 1/1 v/v) to give 2.55 g of yellow solid A-0. Yield: 86%.

**Synthesis of A-1**

Under an argon atmosphere, tris(4-(4,4,5,5-tetramethyl-1,3,2-dioxaborolan-2-yl)phenyl)amine (311.50 mg, 0.50 mmol), A-0 (891.50 mg, 13.00 mmol), Pd(PPh_3_)_4_ (173.30 mg, 0.15 mmol), N,N-dimethylformamide (DMF, 50 mL) and aqueous K_2_CO_3_ solution (5 mL, 1.50 mol/L) were added to a 250 mL round-bottomed flask. The reaction mixture was stirred at 90 ^o^C and the reaction progress was monitored by TLC. After cooling to room temperature, the mixture was rotary evaporated, extracted with CH_2_Cl_2_ and CHCl_3_, the organic phase was dried and the crude product was purified by silica gel column chromatography to give 315.20 mg of red solid A-1. Yield: 71%. ^1^H NMR (400 MHz, Chloroform-*d*) δ (ppm): 8.18 (d, *J* = 3.8, 3H), 8.03-7.99 (m, 9H), 7.80 (d, *J* = 7.5 Hz, 3H), 7.51 (d, *J* = 5.2 Hz, 3H), 7.47 (d, *J* = 8.1 Hz, 6H), 7.27 (dd, *J* = 5.1, 3.7 Hz, 3H). ESI-MS (m/z): C_48_H_27_N_7_S_6_ Calculated: 894.0652 ([M+H]^+^), found: 894.0725 ([M+H]^+^).

**Synthesis of A-2**

A-1 (893.00 mg, 1.00 mmol) was added to a 100 mL round bottom flask and dissolved in CHCl_3_ (80 mL) by heating. N-bromosuccinimide (NBS, 569.60 mg, 3.20 mmol) was added to the clear solution while stirring in an ice bath. The reaction mixture was stirred at room temperature in the dark, followed by rotary evaporation, water washing, suction filtration and vacuum drying to give 1024.20 mg of red solid A-2. Yield: 91%. ^1^H NMR (400 MHz, Chloroform-*d*) δ (ppm): 7.97 (d, *J* = 8.7 Hz, 6H), 7.88 (dd, *J* = 7.5, 2.3 Hz, 3H), 7.83 (dd, *J* = 3.9, 1.4 Hz, 3H), 7.74 (dd, *J* = 7.5, 2.2 Hz, 3H), 7.42 (d, *J* = 8.7 Hz, 6H), 7.17 (d, *J* = 4.1 Hz, 3H). ESI-MS (m/z): C_48_H_24_Br_3_N_7_S_6_ Calculated: 1131.7927 ([M+H]^+^), found: 1131.8005 ([M+H]^+^).

**Synthesis of A-3**

Under an argon atmosphere, A-2 (112.70 mg, 0.10 mmol), 3-pyridineboronic acid (73.40 mg, 0.60 mmol), Pd(PPh_3_)_4_ (34.60 mg, 0.03 mmol), diox (20 mL) and aqueous K_2_CO_3_ solution (2 mL, 1.50 mol/L) were added to a 100 mL round-bottomed flask. The reaction mixture was stirred at 95 ^o^C and the reaction progress was monitored by TLC. After cooling to room temperature, the mixture was rotary evaporated, extracted with CHCl_3_, the organic phase was dried and the crude product was purified by silica gel column chromatography to give 95.20 mg of red solid A-3. Yield: 85%. ^1^H NMR (400 MHz, Chloroform-*d*) δ (ppm): 9.00 (s, 3H), 8.56 (d, *J* = 4.8 Hz, 3H), 8.16 (d, *J* = 3.8 Hz, 3H), 8.01 (d, *J* = 7.9 Hz, 12H), 7.79 (d, *J* = 7.5 Hz, 3H), 7.50 (d, *J* = 3.9 Hz, 3H), 7.45 (d, *J* = 8.2 Hz, 6H), 7.37 (dd, *J* = 8.0, 4.9 Hz, 3H). ESI-MS (m/z): C_63_H_36_N_10_S_6_ Calculated: 1125.1482 ([M+H]^+^), found: 1125.1521 ([M+H]^+^).

**Supplementary Methods**

**Characterization**

Nuclear Magnetic Resonance (^1^H NMR) spectroscopy was obtained on a Bruker AVANCE III spectrometer (400 MHz). Electrospray Ionization Mass Spectrometry (ESI-MS) was conducted on a Bruker ultrafleXtreme instrument. UV-Vis absorption spectra were recorded using a Shimadzu UV-3600 spectrophotometer, while fluorescence emission spectra were measured on an Edinburgh FLS980 spectrometer. Low-temperature (77 K) N_2_ adsorption-desorption isotherms were obtained using a Quantachrome Auto-sorb-iQ2-MP analyzer; specific surface areas were calculated using the Brunauer-Emmett-Teller (BET) method, and pore size distributions were analyzed via the Non-Localized Density Functional Theory (NLDFT) method based on the adsorption isotherms. Transmission Electron Microscopy (TEM) and High-Resolution TEM (HRTEM) were performed on a JEOL JEM-2100F field-emission microscope operating at 300 kV. Scanning Electron Microscopy (SEM) utilized a TESCAN MIRA LMS ultra-high-resolution instrument. Aberration-Corrected High-Angle Annular Dark-Field Scanning TEM (AC-HAADF-STEM) imaging and elemental analysis were carried out using an Oxford energy-dispersive spectrometer integrated with the TEM. Attenuated Total Reflectance Fourier Transform Infrared (ATR-FTIR) spectroscopy was employed. Cyclic Voltammetry (CV) measurements were performed on a CHI-760E electrochemical workstation using a three-electrode configuration (Pt plate working/counter electrodes, saturated Ag/AgCl reference electrode). X-ray Photoelectron Spectroscopy (XPS) was conducted in an ultrahigh vacuum chamber (ESCALAB 250Xi) using a monochromated Al Kα source (*hν* = 1486.6 eV, spot size = 900 μm, pass energy = 20 eV). Inductively Coupled Plasma Atomic Emission Spectroscopy (ICP-AES) on a SPECTRO CIROS VISION instrument quantified the MOC-FA3 content in composites. Surface Photovoltage (SPV) spectra were measured on a CEL-SPS1000 system. Electrochemical Impedance Spectroscopy (EIS) was performed on the CHI-760E workstation in 0.5 M Na_2_SO_4_(aq) using photocatalyst-functionalized FTO as the working electrode. Powder X-ray Diffraction (PXRD) patterns were collected on a PANalytical EMPYREAN diffractometer (reflection geometry, Cu Kα radiation, λ = 1.54 Å, 45 kV, 35 mA). Single-Crystal X-ray Diffraction (SCXRD) data were collected at 150 K on an Agilent Sapphire3 Gemini Ultra diffractometer (Cu radiation, λ = 1.54178 Å, 40 kV, 40 mA); structures were solved by direct methods and refined by full-matrix least-squares on F^2^ using the SHELXTL program package. Piezoresponse Force Microscopy (PFM) and photo-enhanced Kelvin Probe Force Microscopy (KPFM) measurements were conducted using the integrated PFM module and an SKPM module (with a xenon lamp) on a Bruker Dimension Icon Atomic Force Microscope (AFM).

**Piezo-Photocatalytic H_2_ and H_2_O_2_ Evolution under different ultrasonic power or frequency**

Photocatalytic reactions were conducted in a 40 mL cylindrical quartz reactor (Ø 27.5 × 95 mm) containing 20 mL aqueous suspension of photocatalyst (2 mg). The suspension was simultaneously irradiated with a 300 W Xe lamp (PLS-SXE300, Perfect Light; λ ≥ 420 nm cut-off filter) and sonicated using a multi-frequency ultrasonic cleaner (YNLH0203-204060, Shenzhen Yunyi; selectable frequencies: 20/40/60 kHz; corresponding powers: 30/60/120 W) in 0.5 h. Temperature was maintained at 298 K via cooling water circulation. Prior to irradiation, dissolved oxygen was removed by three cycles of: (1) Evacuation (5 min, vacuum pump); (2) Argon purging (5 min). Hydrogen evolution was quantified by GC (FULI GC9790Plus, TCD detector). Hydrogen peroxide production was monitored by sampling 200 μL aliquots at designated intervals for external analysis.

**Measurement of H_2_O_2_**

The quantification of H_2_O_2_ was performed by a TMB-H_2_O_2_-HRP enzymatic assay, using horseradish peroxidase (HRP) as the instantaneous catalyzer of the reaction between H_2_O_2_ and 3,3',5,5'-tetramethylbenzidine (TMB):

| H_2_O_2_ + TMB | HPR | H_2_O + OxTMB | (1) |
| --- | --- | --- | --- |
|  |  |  |  |

TMB Solution**:** 0.015 g of 3,3',5,5'-tetramethylbenzidine (TMB) was dissolved in 0.3 mL dimethyl sulfoxide (DMSO). To this solution, 5 mL glycerol and 45 mL aqueous solution containing 0.02 g ethylenediaminetetraacetic acid (EDTA) and 0.095 g citric acid were added. The mixture was diluted to 500 mL with deionized water.

HRP Solution: Horseradish peroxidase (HRP, 0.002 g) was dissolved in 10 mL deionized water.

H_2_O_2_ Calibration: Standard solutions containing known H_2_O_2_ concentrations (100 μL) were reacted with 200 μL TMB solution and 10 μL HRP solution. After 3 min incubation, 10 μL HCl was added to quench the reaction. Absorbance at 450 nm was measured by UV-vis spectroscopy. A linear calibration curve (absorbance vs. H_2_O_2_ concentration) was generated to quantify H_2_O_2_ in test samples (Figure S31, Supporting Information).

**Apparent quantum yields (AQYs) measurements**

Apparent quantum yields (AQYs) were determined using samples (2 mg) in 0.1 M ascorbic acid water solution (5 mL) under an incident LED light source (Zolix, MLED4-1, λ = 425, 450, 470, 515 and 590 nm, irradiation area 0.8 cm^2^) for 30 min. Light intensity was measured with a solar power meter (SM206-SOLAR). The AQY was calculated as follows:

$$\eta=\frac{2\times M\times N_{A}\times h\times c}{S\times P\times t\times\lambda}\times100\% (2)$$

Where$M$ is the amount of H_2_ produced (mol), $N_{A}$is the Avogadro constant (6.022×10^23^/mol), $h$ is the Planck constant (6.626×10^-34^ J·s), $c$ is the speed of light (3×10^8^ m/s),$S$ is the irradiation area of the incident light (cm^2^ ), $P$ is the intensity of the incident light (W/cm^2^ ), $t$ is the photoreaction time (s), and $\lambda$ is the wavelength of the monochromatic light (m).

**Trapping experiments of Hydroxyl radicals**

Aqueous terephthalic acid (TA) solution (40 mL, 0.5 mM) was prepared in 2 mM NaOH and transferred to a quartz reactor. Photocatalyst (5 mg) was dispersed in the solution. The mixture was irradiated under visible light (300 W Xe lamp, λ > 420 nm, light intensity: 100 mW·cm^-2^) for 1 h at 25 °C with magnetic stirring. Post-irradiation, the suspension was centrifuged (10,000 rpm, 10 min). The fluorescence emission of the supernatant was measured at 425 nm (excitation: 315 nm) using a fluorescence spectrometer.

**Photoelectrochemical measurements**

Photoelectrochemical measurements were conducted using a standard three-electrode system on a CHI660E electrochemical station under visible light (λ ≥ 420 nm). The fabrication of the working electrode was as follows: 2 mg of catalyst was mixed in the solution of 0.5 mL of ethanol and 10 μL of Nafion. After ultrasonic treatment for 5 minutes, 50 μL of the obtained homogeneous suspension was applied to the clean FTO glass (2 × 3 cm). After being further dried and shaped with an infrared lamp, the prepared working electrode was used for photoelectrochemical testing.

**In-situ EPR monitoring experiments**

Electron paramagnetic resonance (EPR) spectra were acquired at 298 K on a Magnettech ESR 5000 spectrometer (X-band, 9.85 GHz) using the following parameters: modulation amplitude 1.000 G, modulation frequency 100 kHz, center field 3500 G, and sweep width 100 G. For in situ radical detection, 10 mg of MOC-FA3/S-COF dispersed in 20 mL aqueous solution containing 200 μL 0.1 M DMPO spin trap was subjected to concurrent ultrasonic irradiation (40 kHz, 60 W) and visible-light illumination (300 W Xe lamp, λ ≥ 420 nm) in a 40 mL quartz reactor. The reaction mixture was continuously circulated through the EPR sample chamber via a peristaltic pump at 1 mL/min flow rate, enabling real-time monitoring at 30-s intervals (Figure S67, Supporting Information).

**High piezoelectric coefficient (d_33_) test**

Powder samples were compressed under 4-ton hydraulic pressure to form 10 mm diameter × 1 mm thick disc-shaped pellets, followed by DC polarization in silicone oil bath at 298 K with 1.4 kV/mm electric field (leakage current maintained at 0.05 mA) for 30 min; piezoelectric coefficient d_33_ was subsequently measured via Berlincourt method using a quasi-static d_33_ meter where 0.25 N alternating force at 110 Hz was applied through upper/lower probes, with generated charge signals processed for real-time d_33_ value display in pC/N units.

**Piezo-photoelectrochemical measurements**

Working electrodes were fabricated by depositing 50 μL catalyst ink (2 mg catalyst ultrasonicated for 5 min in 0.5 mL ethanol + 10 μL 5 *w*t% Nafion) onto pre-cleaned FTO (2×3 cm), followed by precise positioning of a 1×1 cm Nafion 117 proton exchange membrane adjacent to the catalyst layer using thermally stable adhesive film and hot-pressing at 90 °C under 2.76 MPa (400 psi) for 3 min to form an integrated electrode assembly; piezo-photoelectrochemical measurements were subsequently conducted on a CHI660E workstation using a standard three-electrode system (Pt counter, Ag/AgCl reference) in 0.5 M Na_2_SO_4_ electrolyte under concurrent visible-light irradiation (300 W Xe lamp, λ ≥ 420 nm, 100 mW·cm^-2^) and ultrasonic stimulation (40 kHz, 60 W).

**Calculation methods**

Structures in this work were fully optimized by the Vienna Ab initio Simulation Package (version 6.3.0)^1, 2^ using the Perdew-Burke-Ernzerhof (PBE) generalized gradient approximation (GGA)^3, 4^. Zero damping DFT-D3 method of Grimme was used to include the van der Waals interaction^5^. The static energy calculations were carried out with an energy cutoff of 520 eV. The energy levels of models were determined by HSE06 functional. The energy level for the MOC-FA3 was based on its molecular structure with a counter ion of (NO_3_^-^). The energy levels of NO_3_^-^ were excluded in order to find corrected HOMO and LUMO. The density functional perturbation theory was used to determined the piezo tensor of crystals. The born effective charge was determined from the response to finite electric fields, which directly contribute to the piezoelectric tensor. All the data was visualized by the VESTA^6^ and Jmol^7^.

**Supplementary Figs and Tables**

| **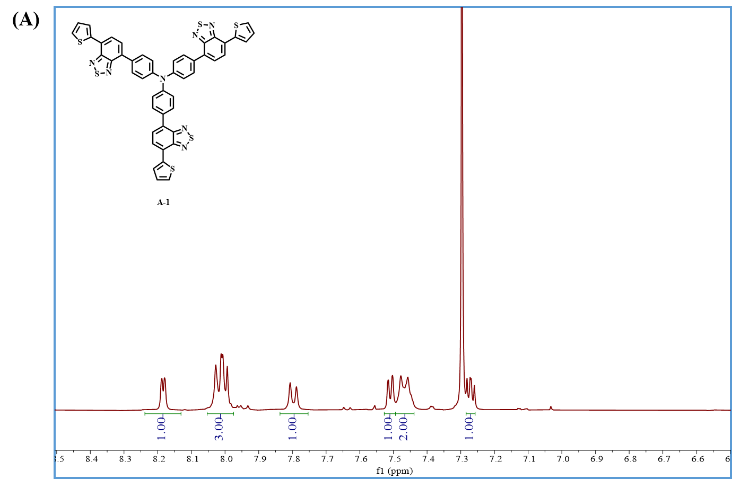** | **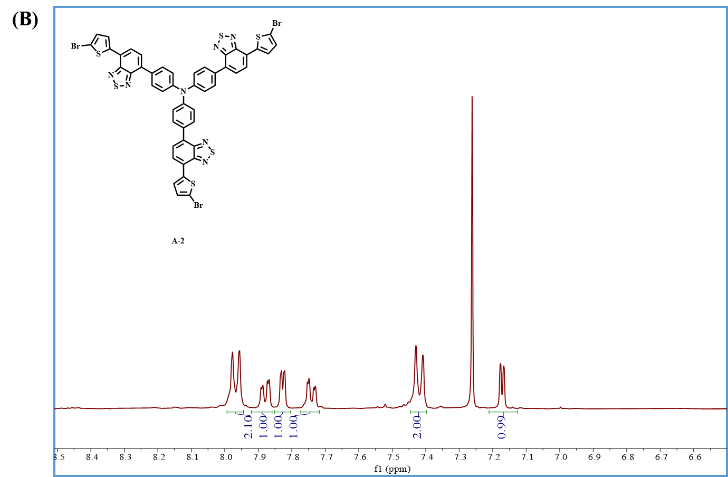** |
| --- | --- |
| **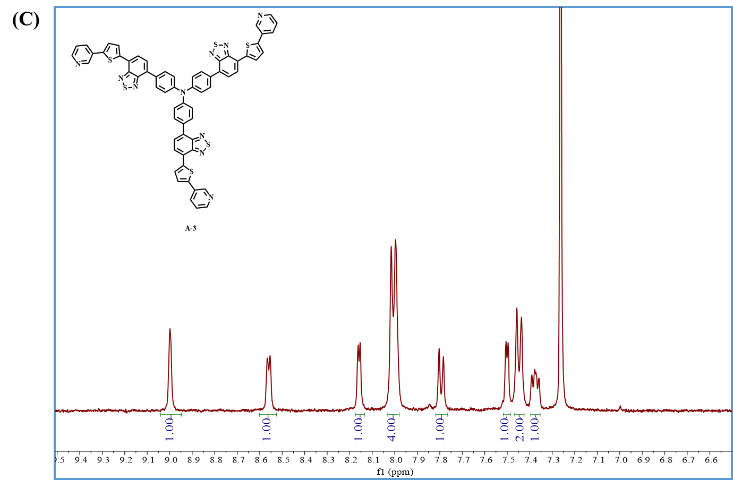** | 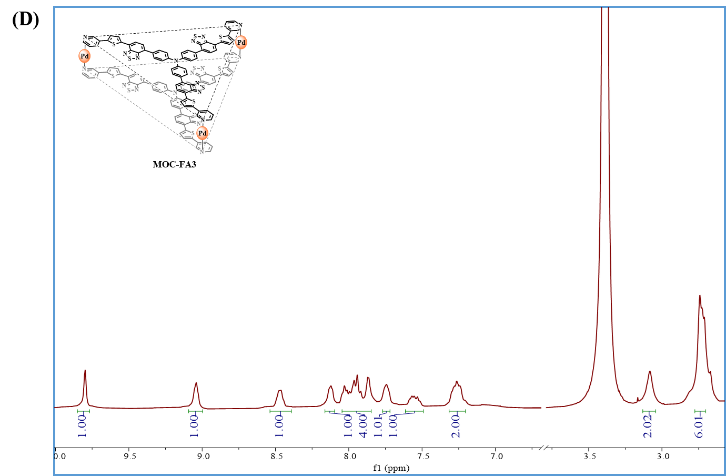 |

1. ^1^H NMR spectra in chloroform-*d* (400 MHz, 300 K) of (A) A-1, (B) A-2, (C) A-3, and ^1^H NMR spectra in DMSO-*d*_6_ (400 MHz, 300 K) of (D) MOC-FA3.

| 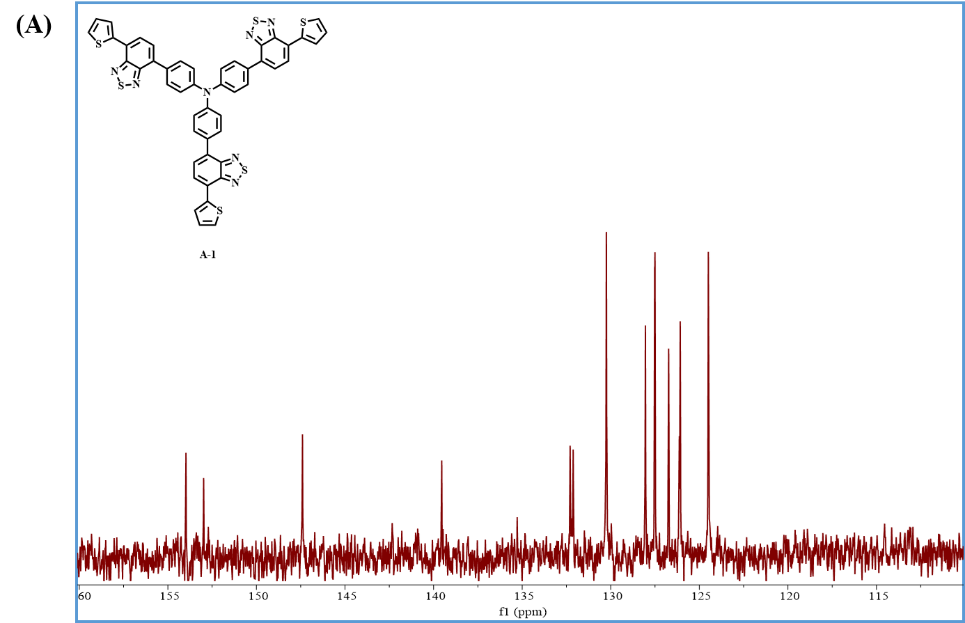 |
| --- |
| 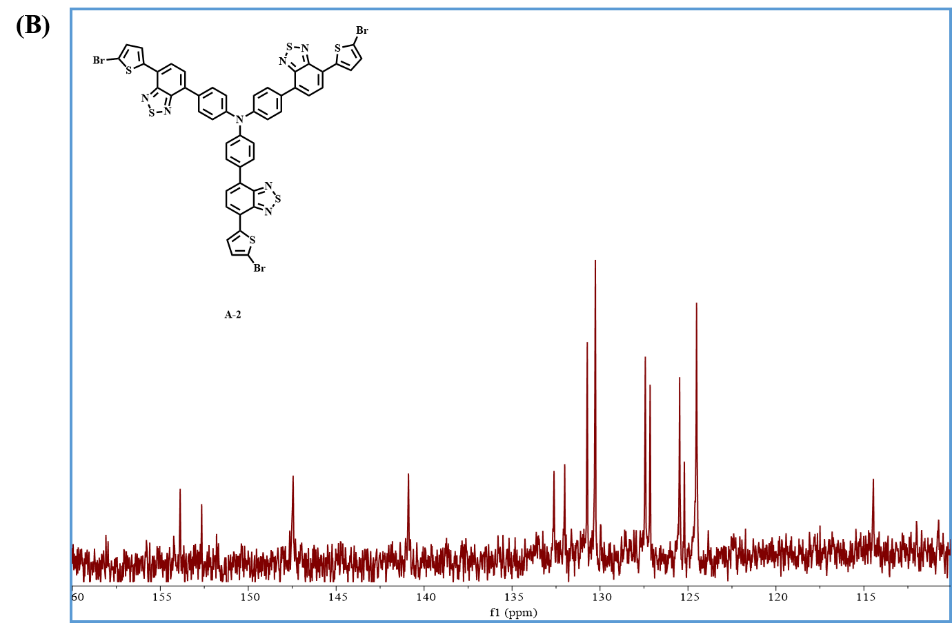 |
| 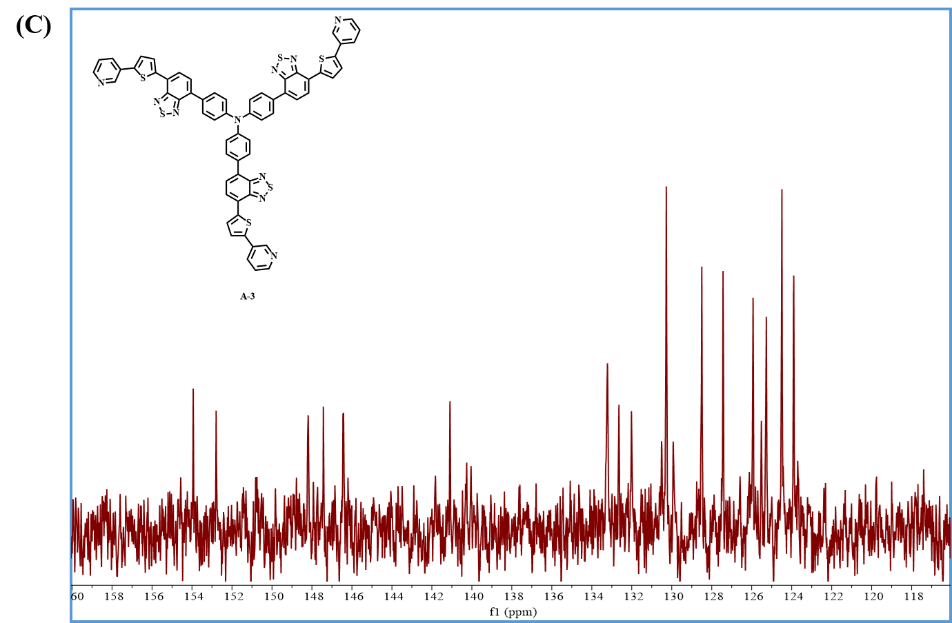 |

1. ^13^C NMR spectra in chloroform-*d* (101 MHz) of (A) A-1, (B) A-2 and (C) A-3.

| 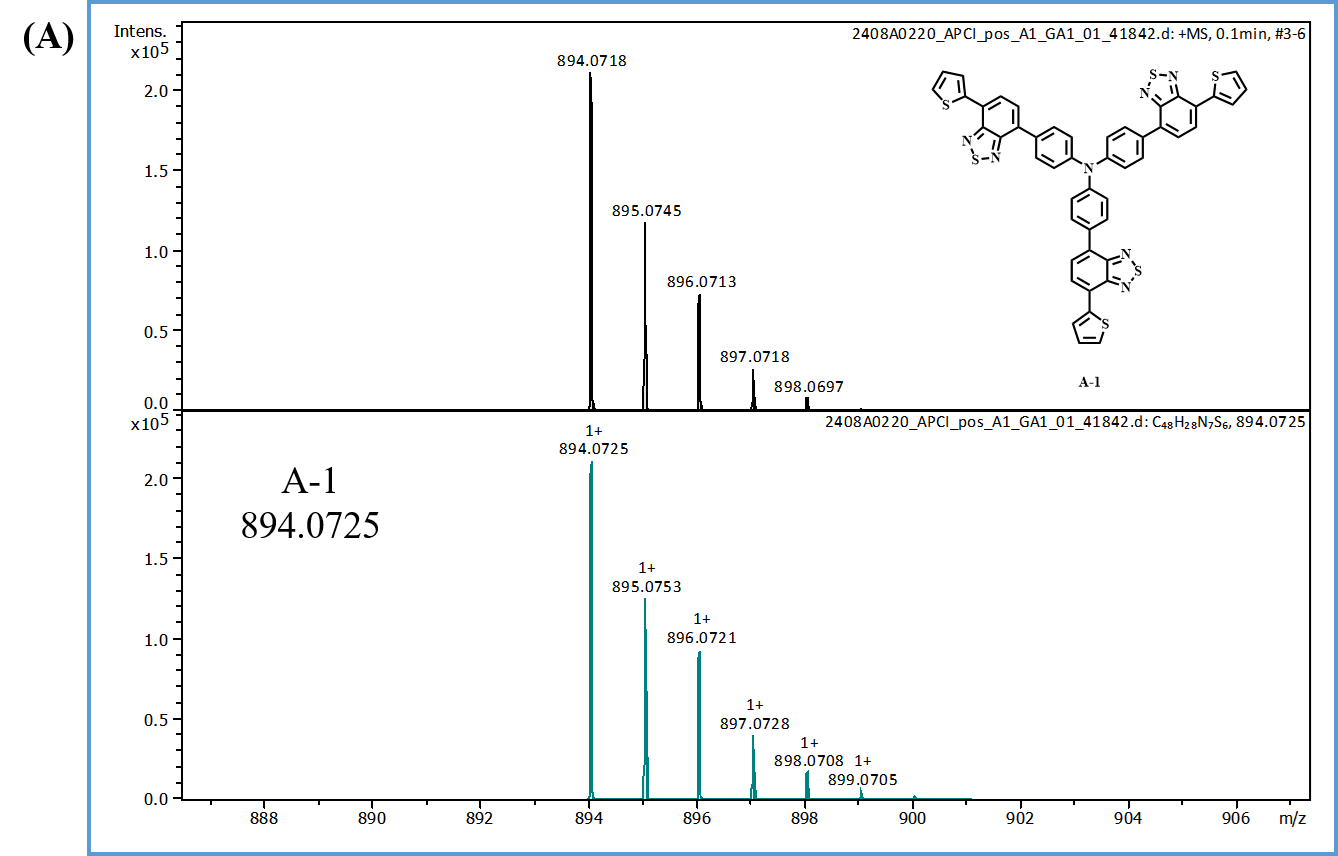 |
| --- |
| 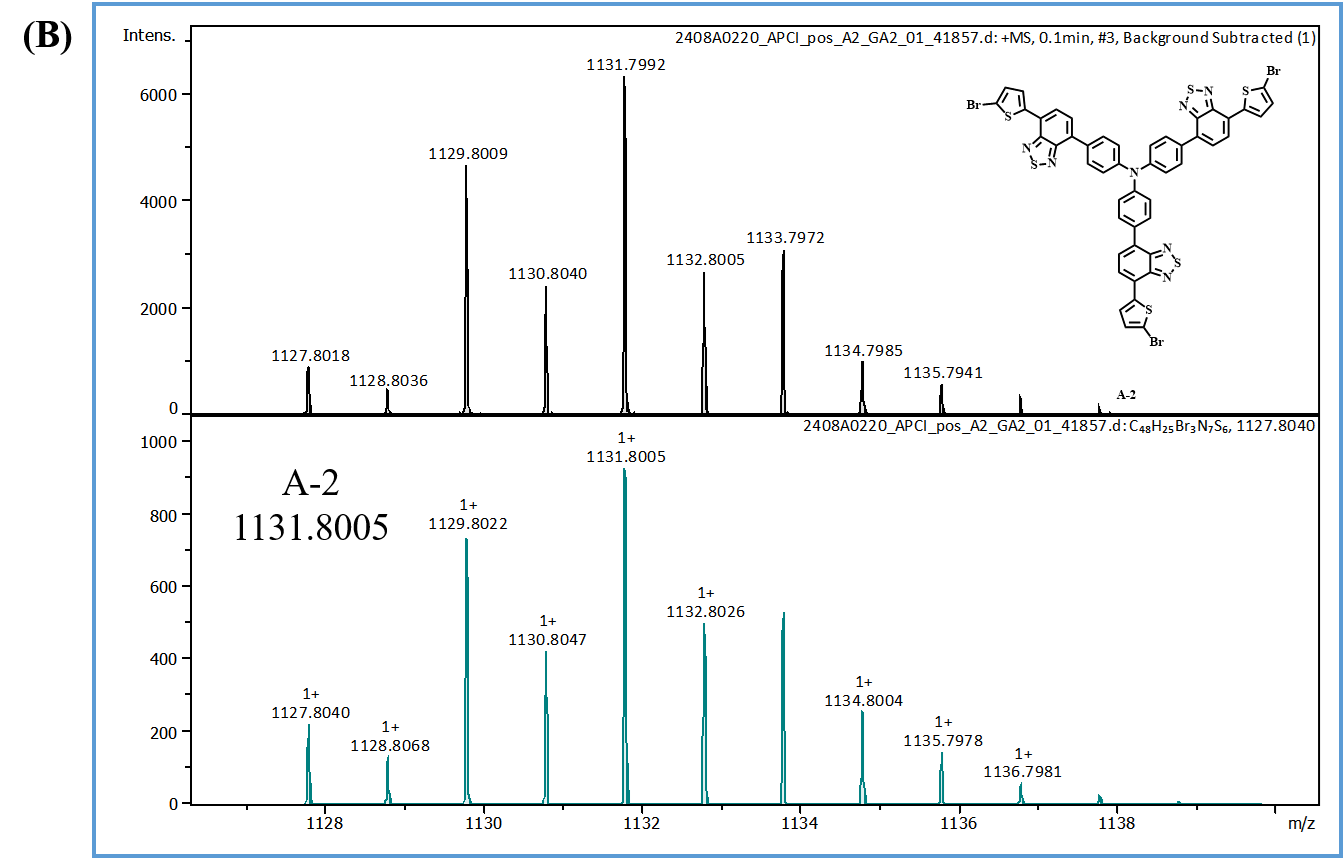 |
| 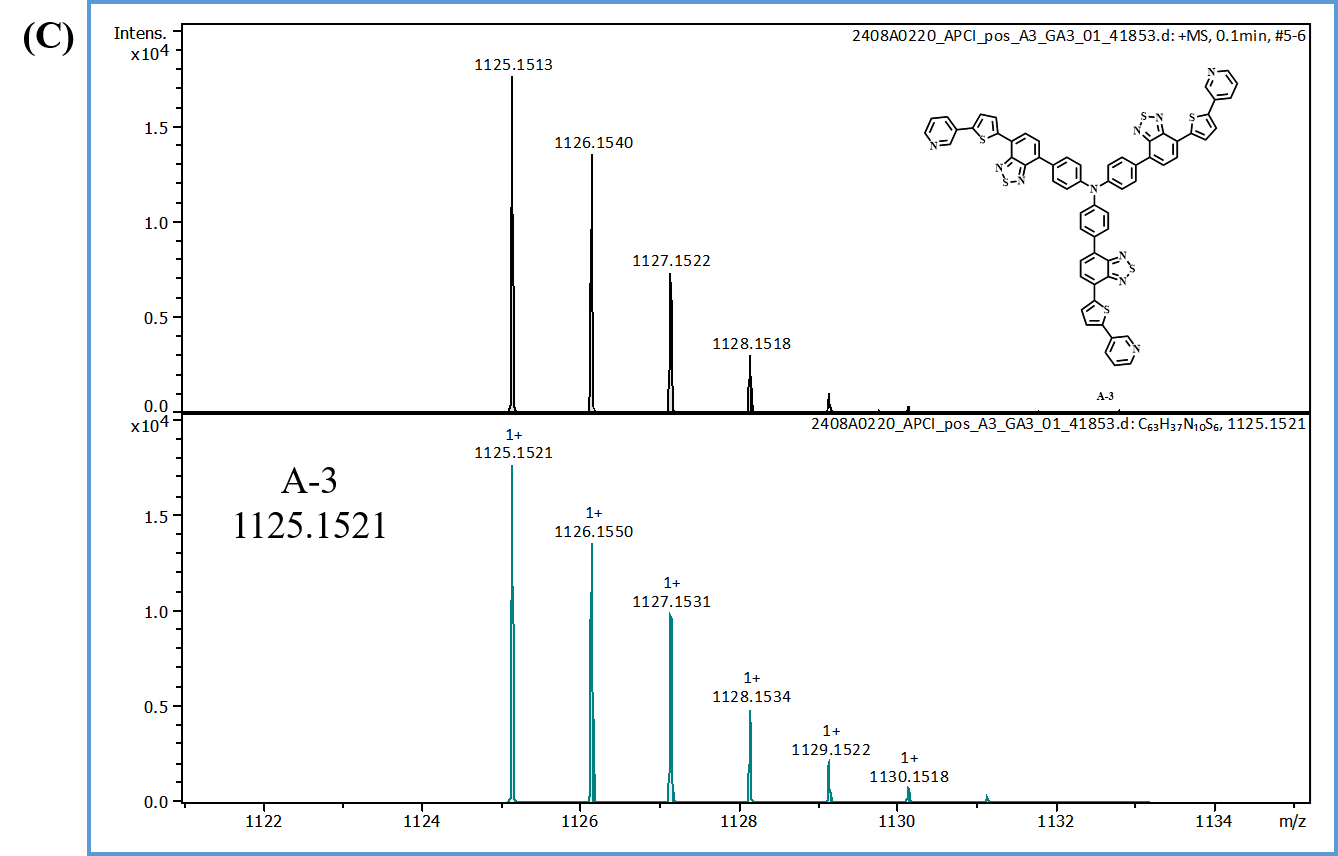 |

1. ESI-MS analysis of (A) A-1, (B) A-2 and (C) A-3.


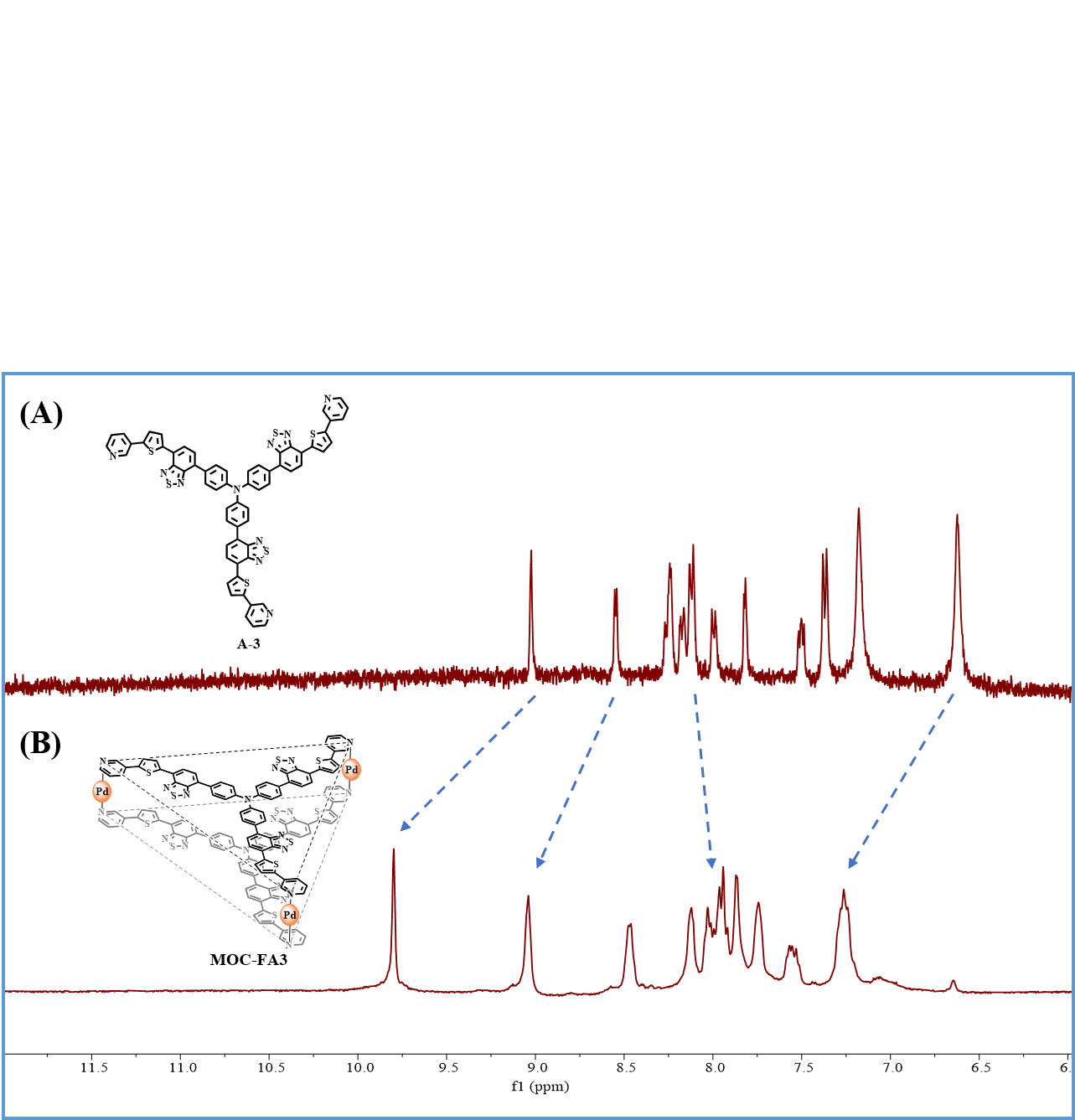


1. Comparative ^1^H NMR spectra in DMSO-*d*_6_ (400 MHz, 300 K) of (A) the ligand A-3 and (B) MOC-FA3.


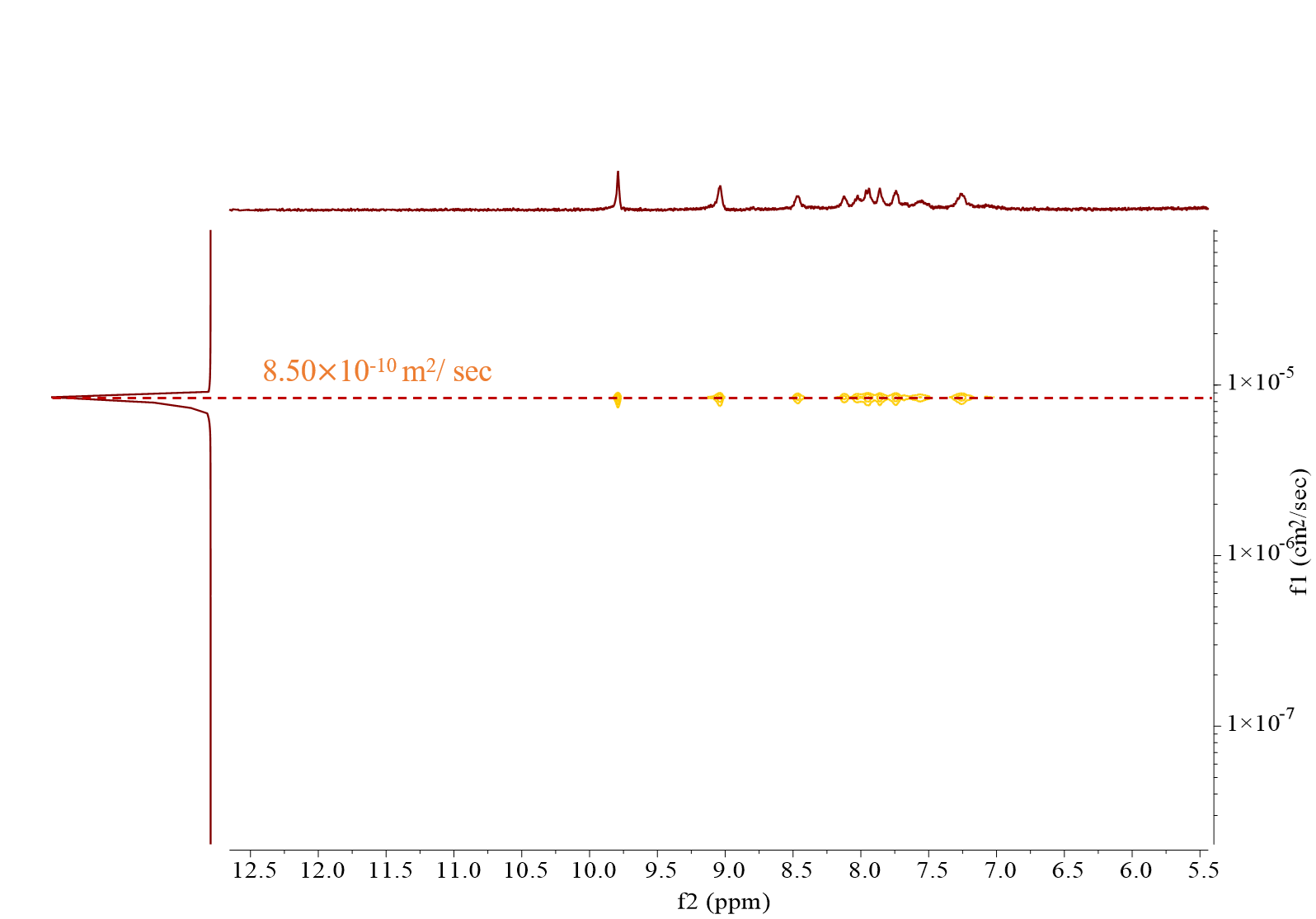


1. ^1^H DOSY spectrum in DMSO-*d*_6_ of MOC-FA3.


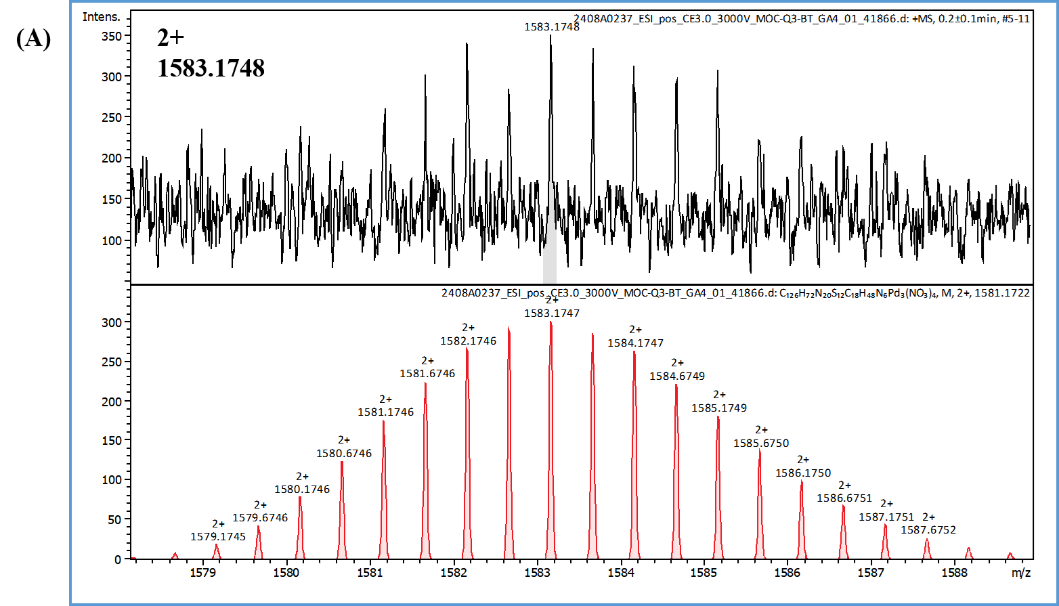


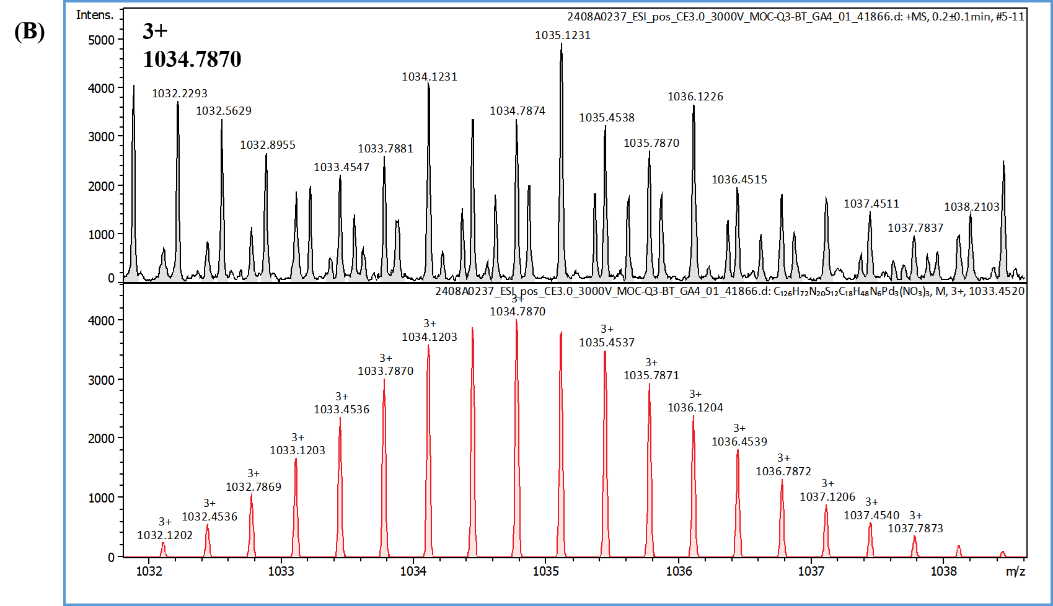


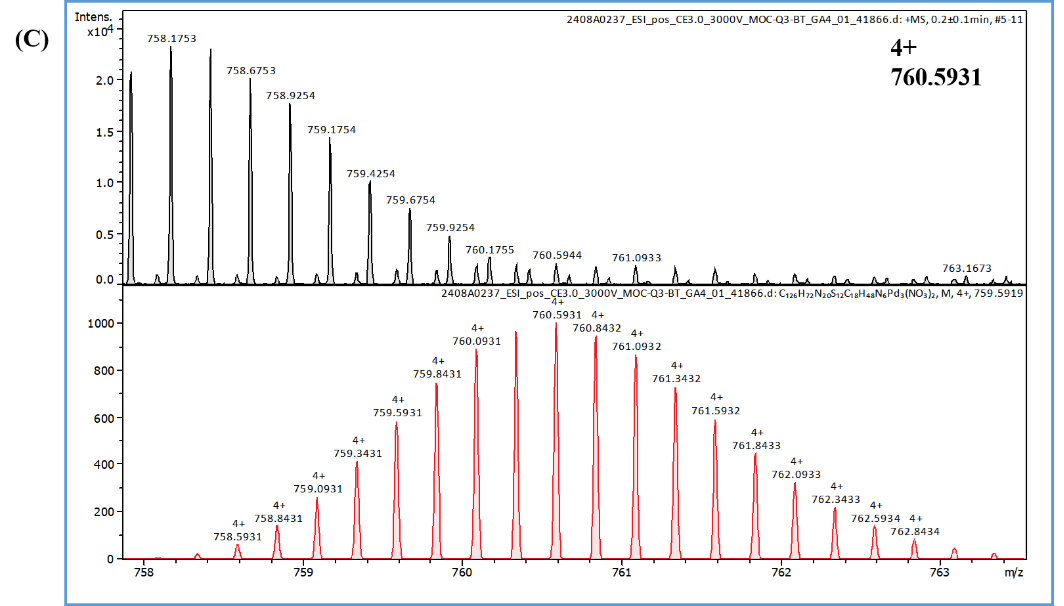


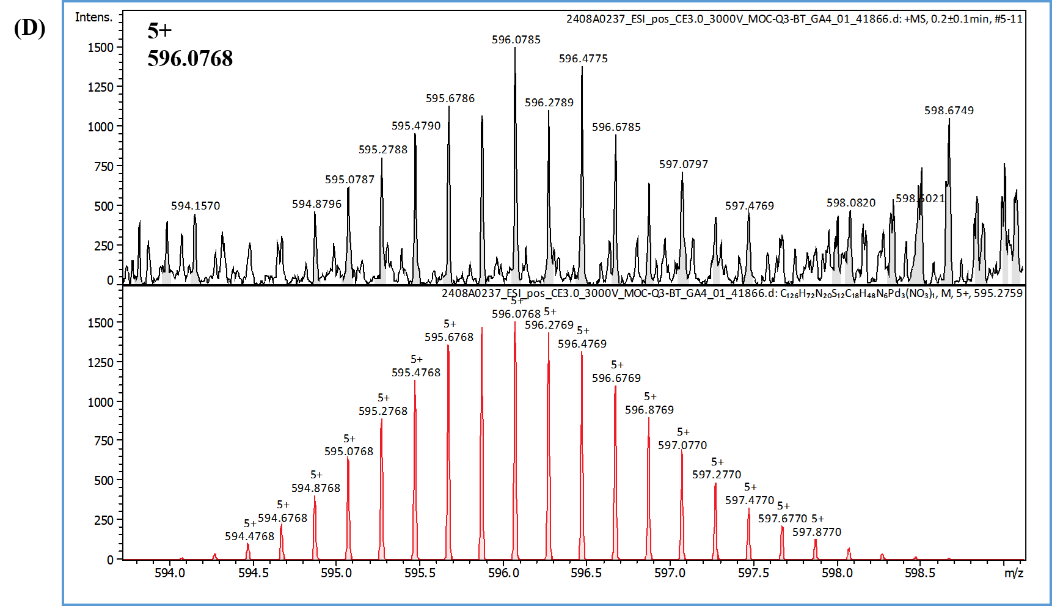


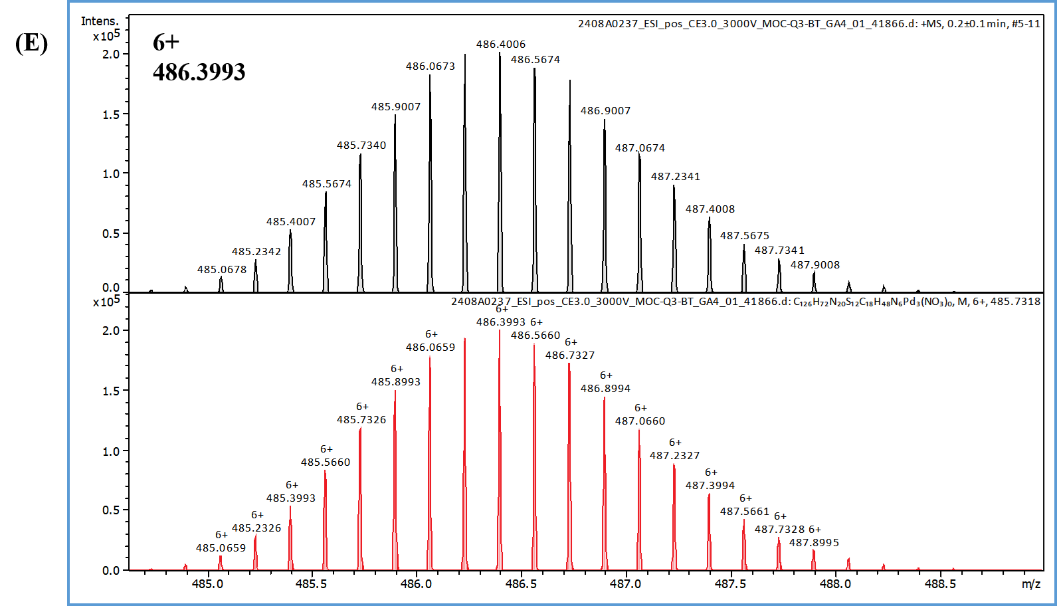


1. ESI-MS analysis of MOC-FA3 showing charge states (2+ to 6+).


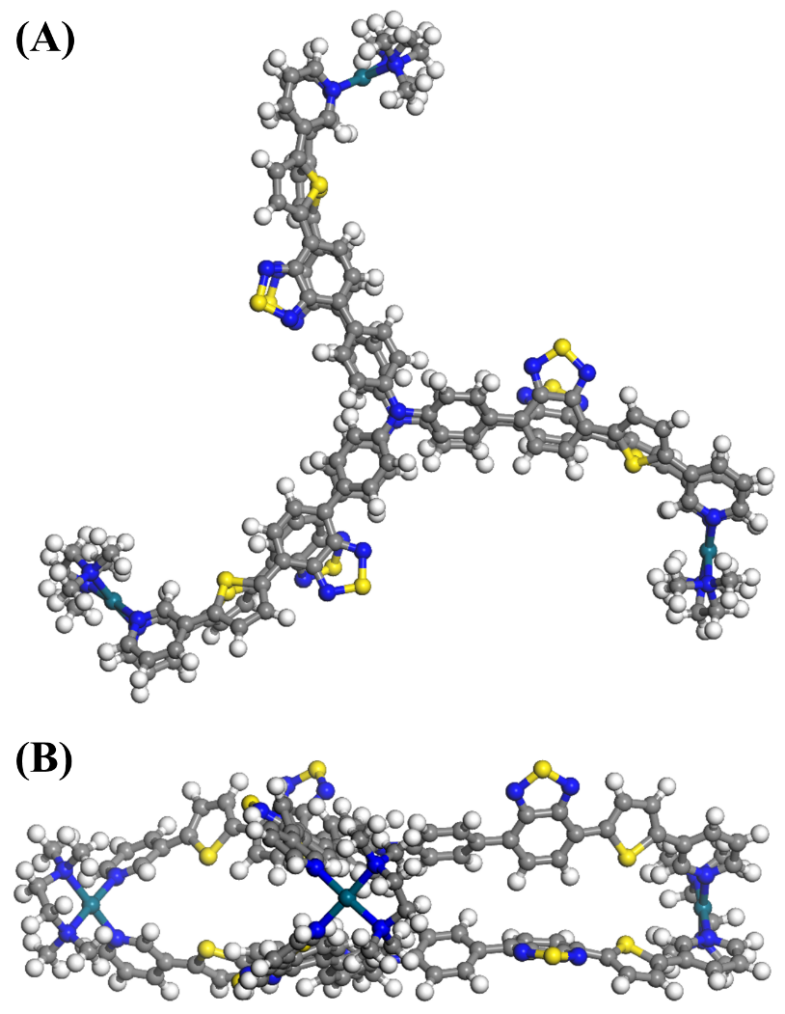


1. Simulated structure of MOC-FA3 showing (A) top and (B) side views.

1. ATR-IR spectra of (A) A-3/MOC-FA3 and (B) TFG/SA/S-COF; (C) ^13^C NMR spectrum and (D) chemical structure of S-COF.

1. (A) Experimental, pawley refined, and simulated PXRD patterns of S-COF; (B) Simulated PXRD patterns of S-COF with AA and AB stacking modes.
2. Fractional atomic coordinates for the unit cell of S-COF.

| S-COF: Space group symmetry *P-6* | | | |
| --- | --- | --- | --- |
| *a* = *b* = 30.1638 Å, *c* = 7.1475 Å | | | |
| *α* = *β* = 90°, *γ* = 120° | | | |
| Atom | x | y | z |
| N1 | 3.47684 | -1.2268 | 0.27938 |
| C2 | 3.39081 | -1.30464 | 0.24689 |
| C3 | 3.36149 | -1.27699 | 0.23879 |
| C4 | 3.96125 | -1.05481 | 0.23940 |
| C5 | 3.94411 | -1.01632 | 0.24738 |
| C6 | 3.89413 | -1.02836 | 0.25377 |
| O7 | 3.93000 | -1.10003 | 0.22037 |
| N8 | 3.85096 | -1.07927 | 0.27851 |
| O9 | 3.69292 | -1.24821 | 0.24874 |
| C10 | 3.55968 | -1.22667 | 0.25851 |
| C11 | 3.55847 | -1.14691 | 0.25113 |
| C12 | 3.61192 | -1.11936 | 0.24393 |
| S13 | 3.65526 | -1.22745 | 0.24945 |
| C14 | 3.61351 | -1.19905 | 0.24950 |
| C15 | 3.63878 | -1.14603 | 0.24418 |
| C16 | 3.79802 | -1.09299 | 0.25954 |
| C17 | 3.76067 | -1.14518 | 0.25533 |
| C18 | 3.70831 | -1.16064 | 0.24857 |
| C19 | 3.69379 | -1.12375 | 0.24432 |
| C20 | 3.73008 | -1.07152 | 0.24614 |
| C21 | 3.78213 | -1.05622 | 0.25402 |
| C22 | 3.53172 | -1.20067 | 0.25975 |
| O23 | 3.60888 | -1.28254 | 0.24969 |
| C24 | 3.44291 | -1.28106 | 0.25293 |
| O25 | 3.38393 | -1.23071 | 0.21923 |
| H26 | 3.46199 | -1.20397 | 0.32402 |
| H27 | 3.88655 | -0.99704 | 0.24440 |
| H28 | 3.85713 | -1.10867 | 0.32250 |
| H29 | 3.54047 | -1.26758 | 0.27042 |
| H30 | 3.53751 | -1.12642 | 0.25140 |
| H31 | 3.63164 | -1.07800 | 0.23928 |
| H32 | 3.77284 | -1.17328 | 0.25915 |
| H33 | 3.71864 | -1.04279 | 0.24430 |
| H34 | 3.80904 | -1.01577 | 0.26061 |
| H35 | 3.45981 | -1.30521 | 0.24170 |

1. MOC-FA3 content in 4% MOC-FA3/S-COF quantified by ICP-MS.

| **Calculated MOC-FA3 content**  **(*w*t%)** | **Actual MOC-FA3 content**  **(*w*t%)** | **Actual Pd content**  **(*w*t%)** | **Actual Pd content**  **(mg/L)** |
| --- | --- | --- | --- |
| 3 | 0.883 | 0.089 | 0.178 |
| 4 | 1.478 | 0.149 | 0.298 |
| 5 | 1.071 | 0.108 | 0.216 |


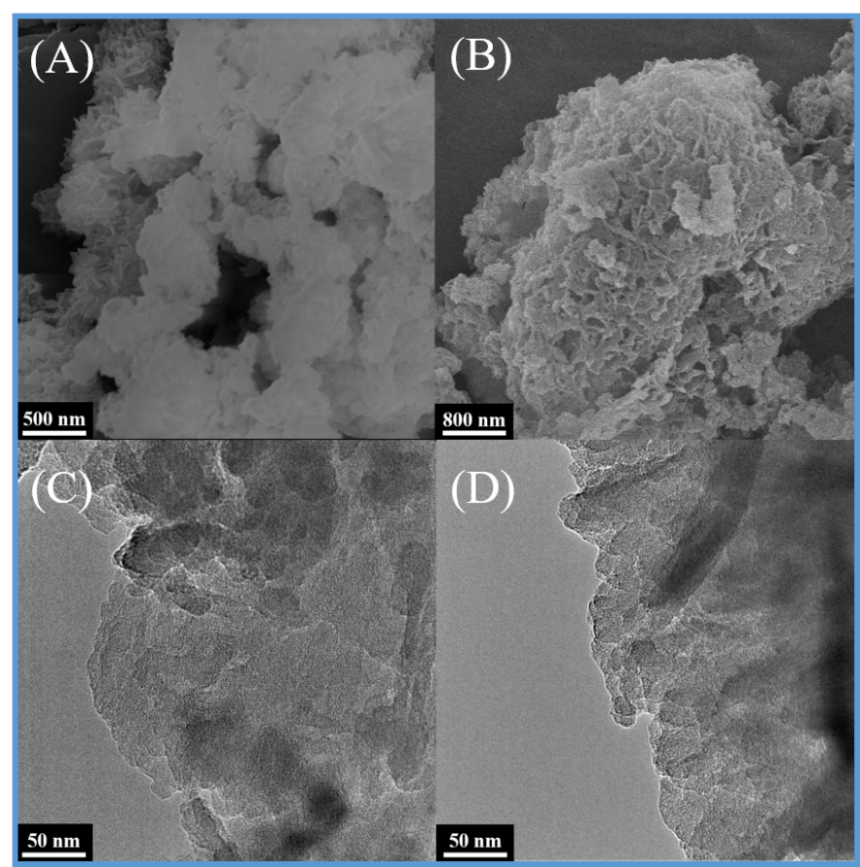


1. SEM images of (A) S-COF and (B) 4% MOC-FA3/S-COF, and TEM images of (C) S-COF and (D) 4% MOC-FA3/S-COF.


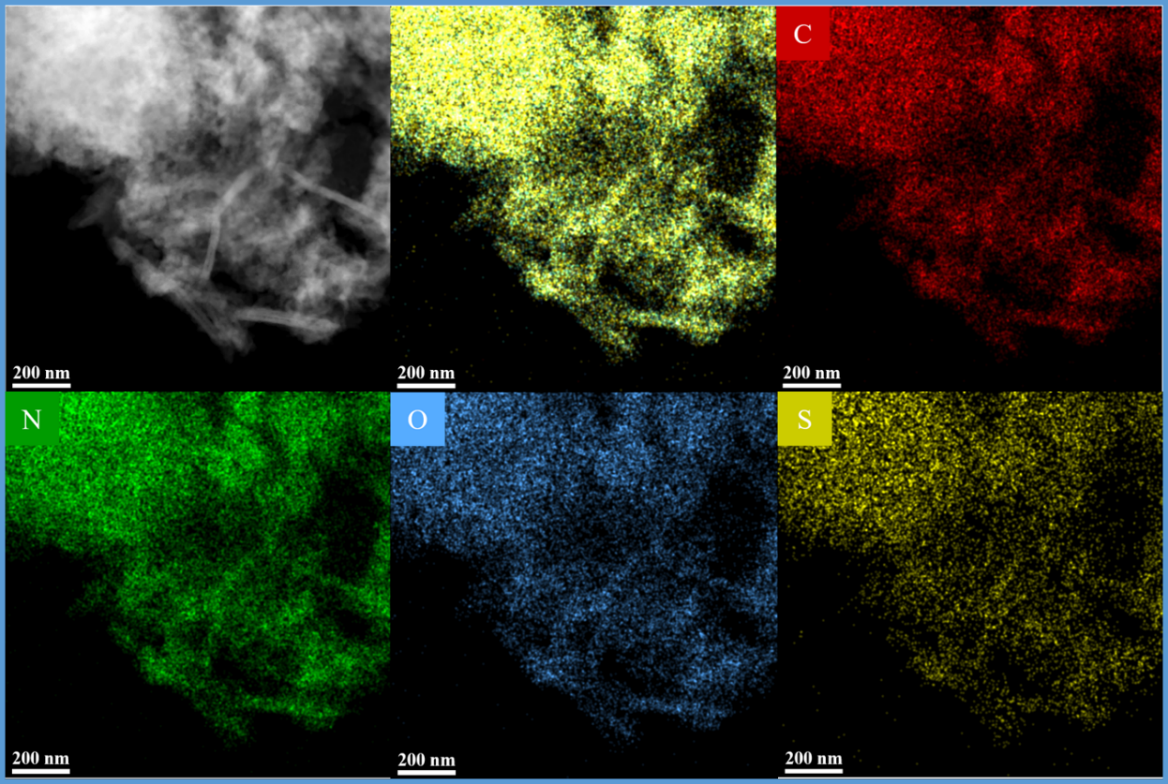


1. TEM elemental mapping results of S-COF.
2. Elemental contents of S-COF as determined by TEM mapping.

| Element | Line Type | Mass Fraction (%) |
| --- | --- | --- |
| C | K | 64.18 |
| N | K | 6.51 |
| O | K | 19.29 |
| S | K | 10.03 |


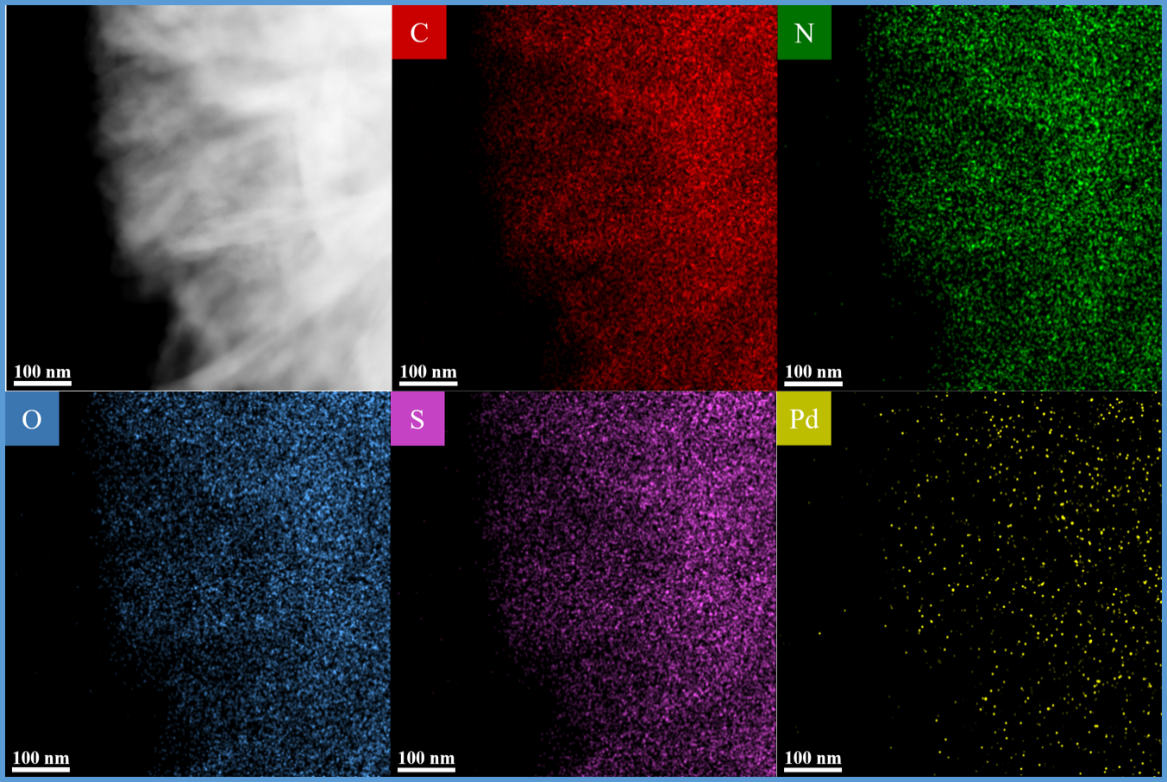


1. TEM elemental mapping results of 4% MOC-FA3/S-COF.
2. Elemental contents of 4% MOC-FA3/S-COF as determined by TEM mapping.

| Element | Line Type | Mass Fraction (%) |
| --- | --- | --- |
| C | K | 71.58 |
| N | K | 6.46 |
| O | K | 10.46 |
| S | K | 11.37 |
| Pd | L | 0.12 |


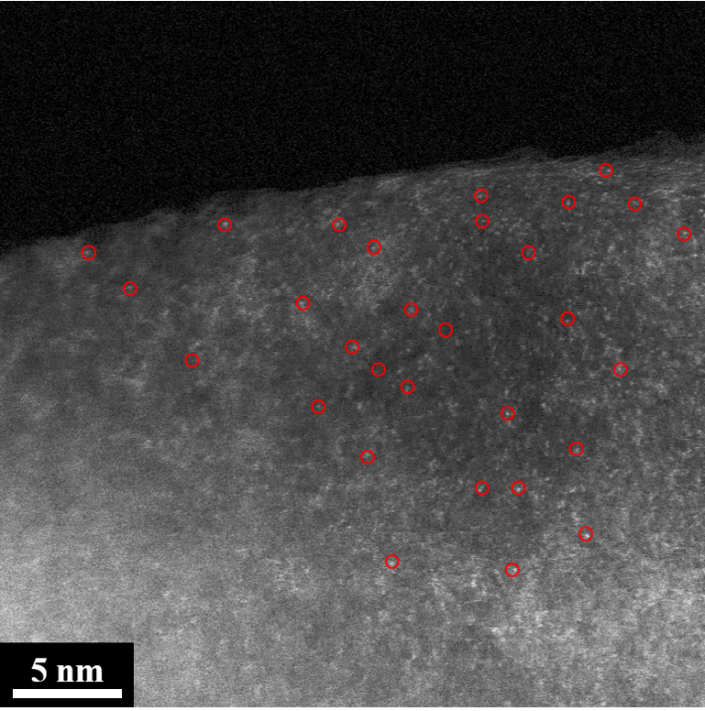


1. AC-HAADF-STEM image of 4% MOC-FA3/S-COF.

1. (A) N_2_ adsorption-desorption isotherms and (B) pore size distribution curves of S-COF; (C) N_2_ adsorption-desorption isotherms and (D) pore size distribution curves of 4% MOC-FA3/S-COF.

1. ATR-IR spectra of S-COF, 4% MOC-FA3/S-COF and MOC-FA3.

1. Pd_3d_ XPS spectra of 4% MOC-FA3/S-COF and MOC-FA3 samples.
2. The corresponding AQYs of 4% MOC-FA3/S-COF.

| Wavelength | 420 nm | 435 nm | 475 nm | 500 nm | 520 nm |
| --- | --- | --- | --- | --- | --- |
| AQY | 0.90% | 0.74% | 1.88% | 2.29% | 1.48% |

1. (A) Pd_3d_ XPS spectra, (B) PXRD patterns, (C) solid UV-Vis absorption spectra and (D) ATR-IR spectra of 4% MOC-FA3/S-COF before and after photocatalysis.

1. Cyclic voltammograms of (A) ferrocene and (B) MOC-FA3 measured in DMSO/CH_3_CN (1:1, v/v) solutions containing 0.1 M TBAPF_6_ at a scan rate of 500 mV/s; (C) UV-Vis absorption and fluorescence emission spectra of MOC-FA3 in DMSO; (D) Schematic energy level diagram of MOC-FA3.
2. Experimental electrochemical parameters and corresponding frontier molecular orbital energy levels.

| Sample  name | E_ox_/V  vs Fc^+^/Fc | E_ox_/V vs. NHE (HOMO) | Intersection/nm | E_0-0_  /eV | E_red_/V vs. NHE  (LUMO) |
| --- | --- | --- | --- | --- | --- |
| MOC-FA3 | 0.73 | 1.03 | 574 | 2.16 | -1.13 |

E_HOMO_ (vs Vac.) = - (E_ox_ (vs Fc^+^/Fe) +4.8) = - (E_ox_ (vs NHE) + 4.5)

E_0-0_ (eV) = 1240/Intersection (nm)

1. (A) Mott-Schottky plot of S-COF in 0.5 M Na_2_SO_4_ (aq); (B) Tauc plot for band gap determination; (C) Valence band (VB) XPS spectrum; (D) Schematic energy level diagram of S-COF constructed from the results of (A-C).

1. Calculated energy levels of the MOC-FA3/S-COF composite.

S-COF + hv → h_VB_^+^ + e_CB_^-^ (1)

e_CB_^-^ + O_2_ → O_2_•^-^  (2)

O_2_•^-^ + H^+^ → HO_2_• (3)

e_CB_^-^ + HO_2_• + H^+^ → H_2_O_2_ (4)

H_2_O_2_ + e_CB_^-^ → •OH + OH^-^ (5)

**Equation** **S1**. Proposed reaction mechanism for hydroxyl radical (•OH) oxidation.


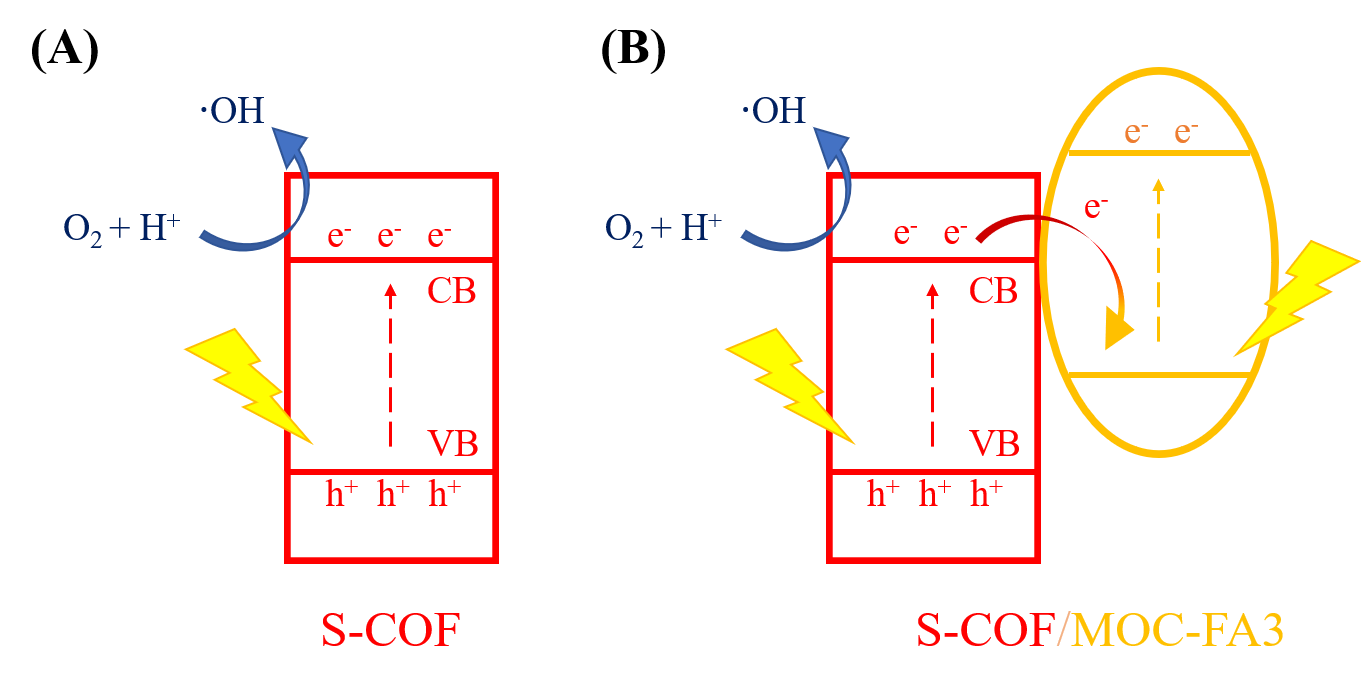


1. Contrasting •OH generation mechanisms: (A) Direct radical formation on S-COF vs. (B) Electron-transfer-mediated •OH production on 4% MOC-FA3/S-COF.

1. SPV spectra of S-COF and 4% MOC-FA3/S-COF.

1. Transient absorption spectra of (A) S-COF and (B) MOC-FA3/S-COF, and transient absorption traces normalized to the 530 nm exciton bands for (C) S-COF and (D) MOC-FA3/S-COF.

1. Photocurrent profiles of S-COF, MOC-FA3 and 4% MOC-FA3/S-COF.

1. EIS Nyquist plots of MOC-FA3, S-COF and 4% MOC-FA3/S-COF measured in the dark (R_1_: Series resistance; R_2_: Charge-transfer resistance; CPE: Constant phase element).
2. Fitting results of ElS Nyquist plots for MOC-FA3, S-COF and 4% MOC-FA3/S-COF under dark.

| Sample | Testing Condition | R_2_ (Ω) | Error (%) |
| --- | --- | --- | --- |
| MOC-FA3 | In the dark | 10910 | 1.77 |
| S-COF | In the dark | 21777 | 7.25 |
| 4% MOC-FA3  /S-COF | In the dark | 25500 | 5.42 |

1. EIS Nyquist plots of MOC-FA3, S-COF and 4% MOC-FA3/S-COF under light irradiation (R_1_: Series resistance; R_2_: Charge-transfer resistance; CPE: Constant phase element).
2. Fitting results of ElS Nyquist plots for MOC-FA3, S-COF and 4% MOC-FA3/S-COF under light irradiation.

| Sample | Testing Condition | R_2_ (Ω) | Error (%) |
| --- | --- | --- | --- |
| MOC-FA3 | Light irradiation | 7113 | 3.80 |
| S-COF | Light irradiation | 6274 | 6.94 |
| 4% MOC-FA3  /S-COF | Light irradiation | 3915 | 4.98 |

1. Applied voltage-dependent resonant peaks for (A) 4% MOC-FA3/S-COF, (B) S-COF, and (C) MOC-FA3.

1. Linear correlation between operating voltage and resonant peak amplitude.


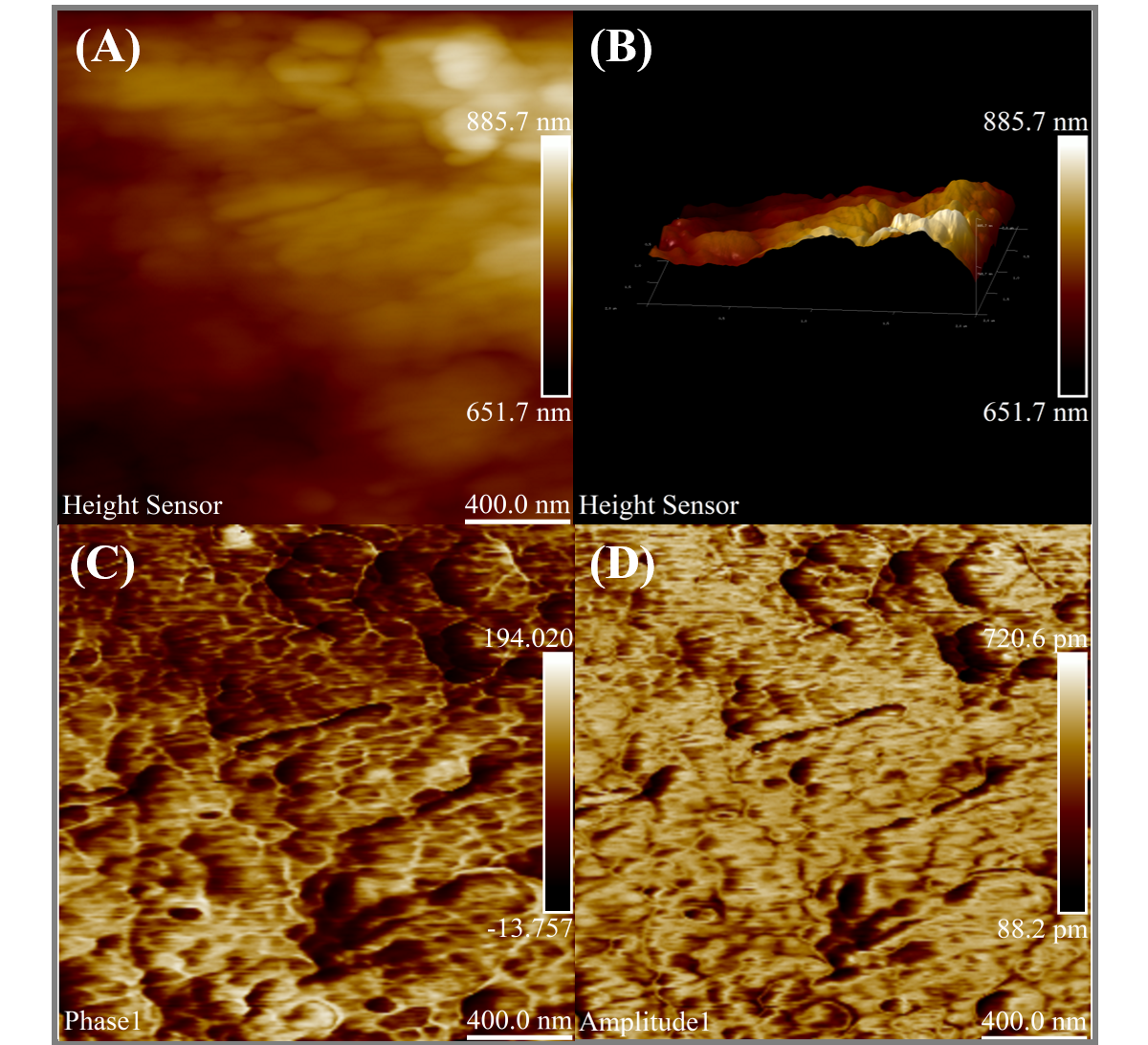


1. AFM characterization of 4% MOC-FA3/S-COF: (A) 2D height topography, (B) 3D rendered topography, (C) PFM amplitude, and (D) PFM phase images.


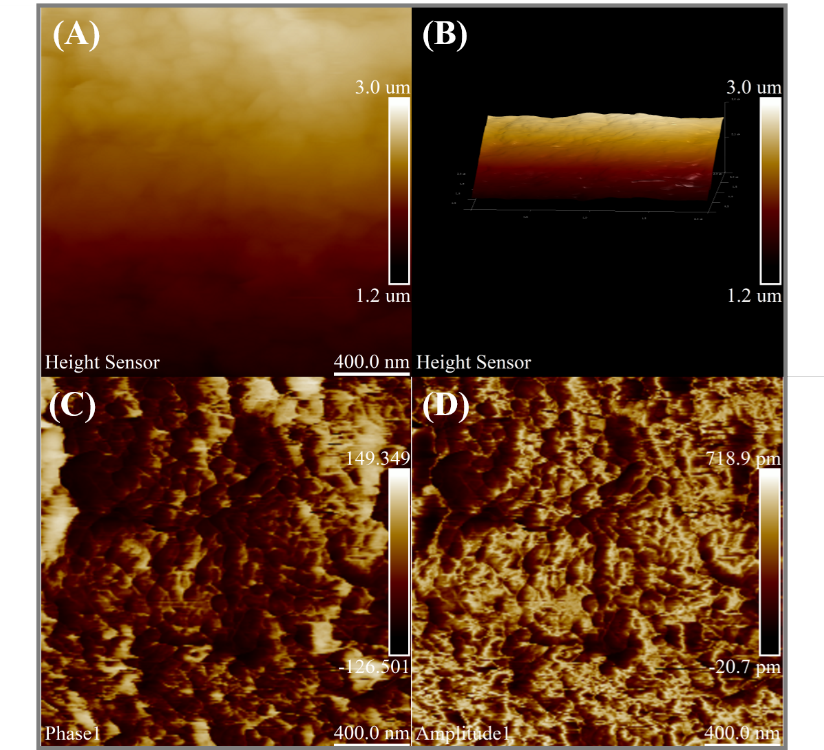


1. AFM characterization of S-COF: (A) 2D height topography, (B) 3D rendered topography, (C) PFM amplitude, and (D) PFM phase images.

1. Overlay of emission spectra: (A) for 4% MOC-FA3/S-COF, S-COF, and reference KDP (1400 nm femtosecond laser illumination); (B) for MOC-FA3 and reference KDP (1200 nm femtosecond laser illumination).


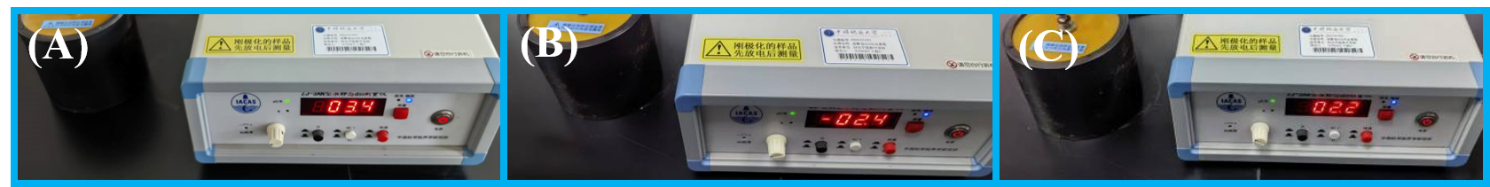


1. Piezoelectric coefficient (*d*_33_) of (A) 4% MOC-FA3/S-COF, (B) S-COF, and (C) MOC-FA3 measured via the Berlincourt quasi-static method.

1. (A) *In situ* high-pressure FT-IR spectra and (B) peak fitting analysis of S-COF.

1. (A) *In situ* high-pressure FT-IR spectra and (B) peak fitting analysis of MOC-FA3.
2. Piezoelectric tensor components of S-COF calculated by DFPT.

| Piezoelectric tensor ionic contr for field in x, y, z (C/m^2^) | | | | | | |
| --- | --- | --- | --- | --- | --- | --- |
|  | XX | YY | ZZ | XY | YZ | ZX |
| X | -0.91453 | 0.91453 | 0.00000 | 0.17863 | -0.00000 | 0.00000 |
| Y | 0.17863 | -0.17863 | -0.00000 | 0.91453 | -0.00000 | -0.00000 |
| Z | 0.00000 | -0.00000 | 0.00000 | 0.00000 | -0.00000 | -0.00000 |

1. Piezoelectric tensor components of MOC-FA3 calculated by DFPT.

| PIEZOELECTRIC TENSOR IONIC CONTR for field in x, y, z (C/m^2^) | | | | | | |
| --- | --- | --- | --- | --- | --- | --- |
|  | XX | YY | ZZ | XY | YZ | ZX |
| X | -0.06633 | -0.26291 | -0.00635 | 0.03467 | 0.13720 | 0.00608 |
| Y | -0.09283 | -0.05422 | -0.24613 | -0.10256 | 0.04282 | 0.05430 |
| Z | -0.09478 | 0.00374 | -0.24499 | 0.06637 | 0.21183 | -0.04006 |


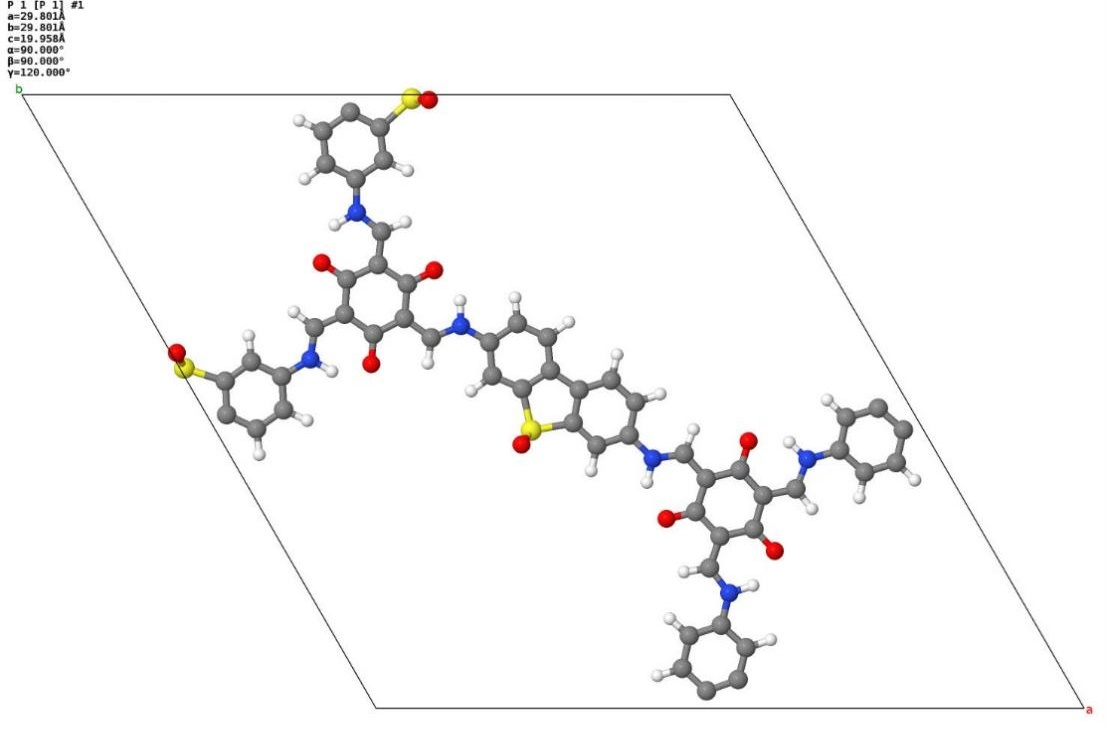


1. Atom-resolved piezoelectric tensor distribution in S-COF calculated by DFPT.


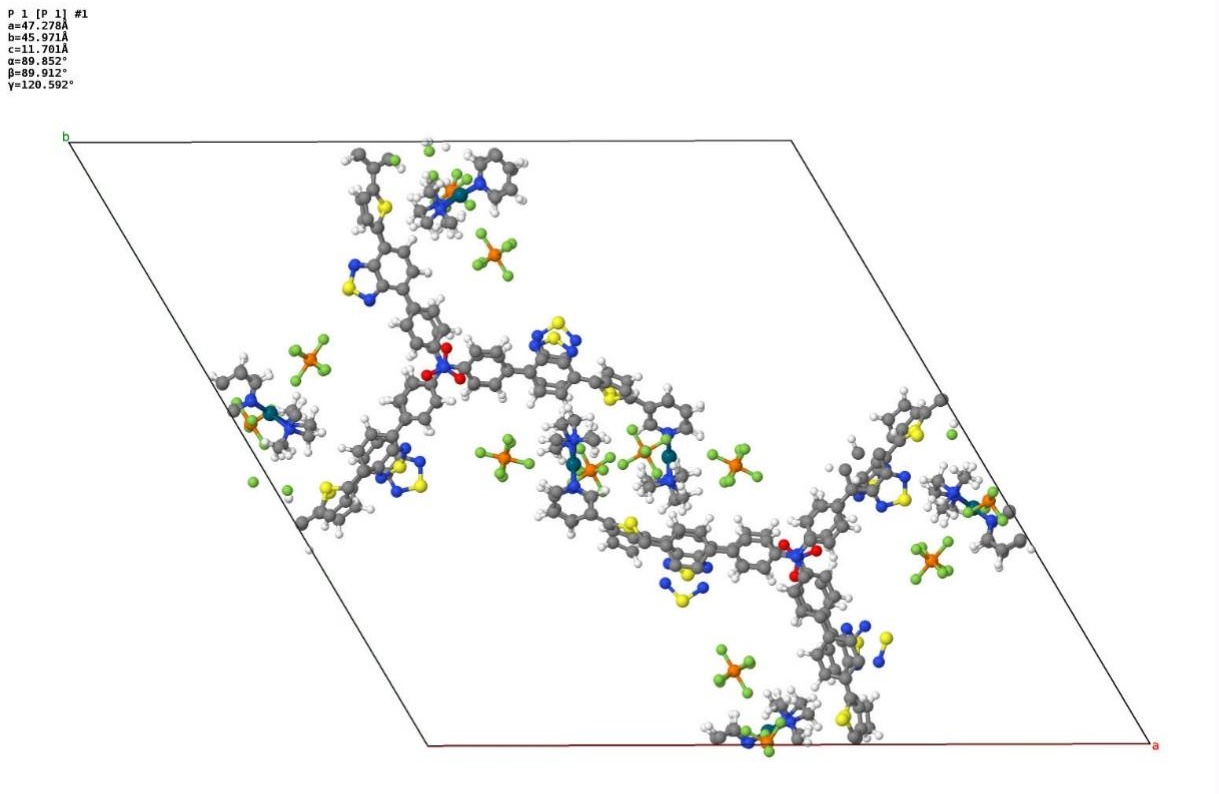


1. Atom-resolved piezoelectric tensor distribution in MOC-FA3 calculated by DFPT.
2. Born effective charge tensors of key atoms in S-COF Computed via DFPT (Unit: |e|)

| ion | 1 |  |  |  | ion | 36 |  |  |  | ion | 71 |  |  |
| --- | --- | --- | --- | --- | --- | --- | --- | --- | --- | --- | --- | --- | --- |
| 1 | -0.852 | -0.036 | -0.159 |  | 1 | -1.319 | 0.253 | 0.000 |  | 1 | -0.387 | -0.250 | 0.000 |
| 2 | -0.031 | -0.812 | 0.329 |  | 2 | -0.346 | -3.909 | 0.000 |  | 2 | 1.368 | -4.483 | 0.000 |
| 3 | -0.056 | 0.114 | -0.690 |  | 3 | 0.000 | 0.000 | -0.183 |  | 3 | 0.000 | 0.000 | -0.203 |
| ion | 2 |  |  |  | ion | 37 |  |  |  | ion | 72 |  |  |
| 1 | -0.851 | 0.031 | -0.206 |  | 1 | 4.221 | -0.144 | 0.000 |  | 1 | -0.553 | 0.083 | 0.000 |
| 2 | 0.037 | -0.812 | -0.302 |  | 2 | -2.055 | 0.117 | 0.000 |  | 2 | 2.220 | -4.080 | 0.000 |
| 3 | -0.071 | -0.105 | -0.690 |  | 3 | 0.000 | 0.000 | 0.000 |  | 3 | 0.000 | 0.000 | -0.217 |
| ion | 3 |  |  |  | ion | 38 |  |  |  | ion | 73 |  |  |
| 1 | -0.793 | -0.003 | 0.365 |  | 1 | 2.660 | -1.345 | 0.000 |  | 1 | 0.086 | 0.173 | 0.000 |
| 2 | 0.002 | -0.871 | -0.027 |  | 2 | -1.383 | 0.729 | 0.000 |  | 2 | 0.130 | 0.191 | 0.000 |
| 3 | 0.126 | -0.009 | -0.690 |  | 3 | 0.000 | 0.000 | -0.010 |  | 3 | 0.000 | 0.000 | 0.238 |
| ion | 4 |  |  |  | ion | 39 |  |  |  | ion | 74 |  |  |
| 1 | -0.852 | -0.036 | 0.159 |  | 1 | -0.265 | 0.096 | 0.000 |  | 1 | 0.979 | -0.265 | 0.000 |
| 2 | -0.031 | -0.812 | -0.329 |  | 2 | 0.003 | -0.180 | 0.000 |  | 2 | 0.055 | 0.085 | 0.000 |
| 3 | 0.056 | -0.114 | -0.690 |  | 3 | 0.000 | 0.000 | -0.211 |  | 3 | 0.000 | 0.000 | 0.327 |
| ion | 5 |  |  |  | ion | 40 |  |  |  | ion | 75 |  |  |
| 1 | -0.851 | 0.031 | 0.206 |  | 1 | -0.838 | -0.130 | 0.000 |  | 1 | 0.030 | 0.158 | 0.000 |
| 2 | 0.037 | -0.812 | 0.302 |  | 2 | 0.516 | 0.063 | 0.000 |  | 2 | 0.080 | 0.217 | 0.000 |
| 3 | 0.071 | 0.105 | -0.690 |  | 3 | 0.000 | 0.000 | -0.095 |  | 3 | 0.000 | 0.000 | 0.237 |
| ion | 6 |  |  |  | ion | 41 |  |  |  | ion | 76 |  |  |
| 1 | -0.793 | -0.003 | -0.365 |  | 1 | -0.103 | 0.793 | 0.000 |  | 1 | 1.107 | -0.087 | 0.000 |
| 2 | 0.002 | -0.871 | 0.027 |  | 2 | 0.029 | -0.532 | 0.000 |  | 2 | 0.149 | 0.083 | 0.000 |
| 3 | -0.126 | 0.009 | -0.690 |  | 3 | 0.000 | 0.000 | -0.144 |  | 3 | 0.000 | 0.000 | 0.320 |
| ion | 7 |  |  |  | ion | 42 |  |  |  | ion | 77 |  |  |
| 1 | -1.510 | 0.356 | 0.000 |  | 1 | -0.065 | -0.109 | 0.000 |  | 1 | -0.002 | 0.101 | 0.000 |
| 2 | 0.388 | -1.186 | 0.000 |  | 2 | 0.122 | 0.154 | 0.000 |  | 2 | 0.010 | 0.077 | 0.000 |
| 3 | 0.000 | 0.000 | -0.418 |  | 3 | 0.000 | 0.000 | -0.135 |  | 3 | 0.000 | 0.000 | 0.263 |
| ion | 8 |  |  |  | ion | 43 |  |  |  | ion | 78 |  |  |
| 1 | -1.622 | 0.143 | 0.000 |  | 1 | 0.242 | -0.094 | 0.000 |  | 1 | 0.105 | -0.096 | 0.000 |
| 2 | 0.412 | -1.121 | 0.000 |  | 2 | -0.209 | -0.112 | 0.000 |  | 2 | -0.019 | -0.078 | 0.000 |
| 3 | 0.000 | 0.000 | -0.431 |  | 3 | 0.000 | 0.000 | -0.079 |  | 3 | 0.000 | 0.000 | 0.261 |
| ion | 9 |  |  |  | ion | 44 |  |  |  | ion | 79 |  |  |
| 1 | -0.944 | -0.062 | 0.000 |  | 1 | -0.121 | 0.814 | 0.000 |  | 1 | -0.044 | 0.082 | 0.000 |
| 2 | -0.030 | -1.751 | 0.000 |  | 2 | 0.010 | -0.495 | 0.000 |  | 2 | 0.058 | 0.024 | 0.000 |
| 3 | 0.000 | 0.000 | -0.418 |  | 3 | 0.000 | 0.000 | -0.136 |  | 3 | 0.000 | 0.000 | 0.254 |
| ion | 10 |  |  |  | ion | 45 |  |  |  | ion | 80 |  |  |
| 1 | -1.006 | -0.057 | 0.000 |  | 1 | -0.482 | -0.020 | 0.000 |  | 1 | 0.150 | -0.049 | 0.000 |
| 2 | 0.212 | -1.737 | 0.000 |  | 2 | 0.258 | -0.176 | 0.000 |  | 2 | 0.059 | -0.033 | 0.000 |
| 3 | 0.000 | 0.000 | -0.431 |  | 3 | 0.000 | 0.000 | -0.188 |  | 3 | 0.000 | 0.000 | 0.248 |
| ion | 11 |  |  |  | ion | 46 |  |  |  | ion | 81 |  |  |
| 1 | -1.589 | -0.342 | 0.000 |  | 1 | 0.224 | 0.072 | 0.000 |  | 1 | 0.077 | -0.021 | 0.000 |
| 2 | -0.311 | -1.107 | 0.000 |  | 2 | -0.262 | -0.121 | 0.000 |  | 2 | 0.002 | -0.088 | 0.000 |
| 3 | 0.000 | 0.000 | -0.418 |  | 3 | 0.000 | 0.000 | -0.055 |  | 3 | 0.000 | 0.000 | 0.261 |
| ion | 12 |  |  |  | ion | 47 |  |  |  | ion | 82 |  |  |
| 1 | -1.487 | -0.490 | 0.000 |  | 1 | 0.281 | -0.054 | 0.000 |  | 1 | -0.098 | 0.050 | 0.000 |
| 2 | -0.221 | -1.256 | 0.000 |  | 2 | -0.093 | 0.101 | 0.000 |  | 2 | 0.003 | 0.077 | 0.000 |
| 3 | 0.000 | 0.000 | -0.431 |  | 3 | 0.000 | 0.000 | -0.108 |  | 3 | 0.000 | 0.000 | 0.268 |
| ion | 13 |  |  |  | ion | 48 |  |  |  | ion | 83 |  |  |
| 1 | 3.927 | -0.514 | 0.000 |  | 1 | -0.952 | -0.132 | 0.000 |  | 1 | 0.295 | -0.009 | 0.000 |
| 2 | -0.167 | 0.629 | 0.000 |  | 2 | 0.484 | 0.099 | 0.000 |  | 2 | -0.052 | -0.019 | 0.000 |
| 3 | 0.000 | 0.000 | 0.074 |  | 3 | 0.000 | 0.000 | -0.138 |  | 3 | 0.000 | 0.000 | 0.238 |
| ion | 14 |  |  |  | ion | 49 |  |  |  | ion | 84 |  |  |
| 1 | -2.834 | 1.817 | 0.000 |  | 1 | 1.748 | 1.425 | 0.000 |  | 1 | 0.217 | -0.495 | 0.000 |
| 2 | 0.704 | -2.356 | 0.000 |  | 2 | 1.772 | 2.807 | 0.000 |  | 2 | -0.175 | 0.847 | 0.000 |
| 3 | 0.000 | 0.000 | -0.189 |  | 3 | 0.000 | 0.000 | 0.074 |  | 3 | 0.000 | 0.000 | 0.327 |
| ion | 15 |  |  |  | ion | 50 |  |  |  | ion | 85 |  |  |
| 1 | 2.942 | 2.972 | 0.000 |  | 1 | -3.567 | -0.281 | 0.000 |  | 1 | 0.274 | 0.061 | 0.000 |
| 2 | 1.563 | 1.623 | 0.000 |  | 2 | -1.393 | -1.623 | 0.000 |  | 2 | -0.017 | -0.027 | 0.000 |
| 3 | 0.000 | 0.000 | 0.006 |  | 3 | 0.000 | 0.000 | -0.189 |  | 3 | 0.000 | 0.000 | 0.237 |
| ion | 16 |  |  |  | ion | 51 |  |  |  | ion | 86 |  |  |
| 1 | 2.887 | 0.957 | 0.000 |  | 1 | -0.010 | 0.142 | 0.000 |  | 1 | 0.366 | -0.577 | 0.000 |
| 2 | 1.407 | 0.455 | 0.000 |  | 2 | -1.267 | 4.575 | 0.000 |  | 2 | -0.341 | 0.824 | 0.000 |
| 3 | 0.000 | 0.000 | 0.020 |  | 3 | 0.000 | 0.000 | 0.006 |  | 3 | 0.000 | 0.000 | 0.320 |
| ion | 17 |  |  |  | ion | 52 |  |  |  | ion | 87 |  |  |
| 1 | 3.970 | 0.196 | 0.000 |  | 1 | 0.039 | 0.238 | 0.000 |  | 1 | 0.106 | 0.052 | 0.000 |
| 2 | 0.176 | 0.605 | 0.000 |  | 2 | 0.687 | 3.303 | 0.000 |  | 2 | -0.039 | -0.031 | 0.000 |
| 3 | 0.000 | 0.000 | 0.077 |  | 3 | 0.000 | 0.000 | 0.020 |  | 3 | 0.000 | 0.000 | 0.263 |
| ion | 18 |  |  |  | ion | 53 |  |  |  | ion | 88 |  |  |
| 1 | -3.221 | 1.444 | 0.000 |  | 1 | 1.286 | 1.374 | 0.000 |  | 1 | -0.082 | -0.089 | 0.000 |
| 2 | 0.845 | -2.007 | 0.000 |  | 2 | 1.354 | 3.290 | 0.000 |  | 2 | -0.012 | 0.109 | 0.000 |
| 3 | 0.000 | 0.000 | -0.183 |  | 3 | 0.000 | 0.000 | 0.077 |  | 3 | 0.000 | 0.000 | 0.261 |
| ion | 19 |  |  |  | ion | 54 |  |  |  | ion | 89 |  |  |
| 1 | 2.095 | 3.283 | 0.000 |  | 1 | -3.301 | -0.798 | 0.000 |  | 1 | 0.067 | 0.006 | 0.000 |
| 2 | 1.371 | 2.243 | 0.000 |  | 2 | -1.398 | -1.926 | 0.000 |  | 2 | -0.017 | -0.087 | 0.000 |
| 3 | 0.000 | 0.000 | 0.000 |  | 3 | 0.000 | 0.000 | -0.183 |  | 3 | 0.000 | 0.000 | 0.254 |
| ion | 20 |  |  |  | ion | 55 |  |  |  | ion | 90 |  |  |
| 1 | 2.393 | 1.537 | 0.000 |  | 1 | 0.191 | -0.272 | 0.000 |  | 1 | 0.018 | -0.136 | 0.000 |
| 2 | 1.499 | 0.996 | 0.000 |  | 2 | -2.183 | 4.147 | 0.000 |  | 2 | -0.028 | 0.100 | 0.000 |
| 3 | 0.000 | 0.000 | -0.010 |  | 3 | 0.000 | 0.000 | 0.000 |  | 3 | 0.000 | 0.000 | 0.248 |
| ion | 21 |  |  |  | ion | 56 |  |  |  | ion | 91 |  |  |
| 1 | -0.244 | -0.015 | 0.000 |  | 1 | 0.031 | -0.135 | 0.000 |  | 1 | -0.055 | -0.078 | 0.000 |
| 2 | -0.108 | -0.200 | 0.000 |  | 2 | -0.173 | 3.359 | 0.000 |  | 2 | -0.055 | 0.044 | 0.000 |
| 3 | 0.000 | 0.000 | -0.211 |  | 3 | 0.000 | 0.000 | -0.010 |  | 3 | 0.000 | 0.000 | 0.261 |
| ion | 22 |  |  |  | ion | 57 |  |  |  | ion | 92 |  |  |
| 1 | -0.330 | -0.810 | 0.000 |  | 1 | -0.158 | 0.058 | 0.000 |  | 1 | 0.056 | 0.087 | 0.000 |
| 2 | -0.163 | -0.446 | 0.000 |  | 2 | -0.034 | -0.287 | 0.000 |  | 2 | 0.039 | -0.077 | 0.000 |
| 3 | 0.000 | 0.000 | -0.095 |  | 3 | 0.000 | 0.000 | -0.211 |  | 3 | 0.000 | 0.000 | 0.268 |
| ion | 23 |  |  |  | ion | 58 |  |  |  | ion | 93 |  |  |
| 1 | -0.781 | 0.362 | 0.000 |  | 1 | 0.004 | -0.030 | 0.000 |  | 1 | 0.034 | -0.099 | 0.000 |
| 2 | -0.402 | 0.146 | 0.000 |  | 2 | 0.617 | -0.780 | 0.000 |  | 2 | -0.142 | 0.243 | 0.000 |
| 3 | 0.000 | 0.000 | -0.144 |  | 3 | 0.000 | 0.000 | -0.095 |  | 3 | 0.000 | 0.000 | 0.238 |
| ion | 24 |  |  |  | ion | 59 |  |  |  | ion | 94 |  |  |
| 1 | 0.093 | -0.213 | 0.000 |  | 1 | -0.069 | -0.009 | 0.000 |  | 1 | 0.400 | 0.280 | 0.000 |
| 2 | 0.017 | -0.005 | 0.000 |  | 2 | -0.773 | -0.567 | 0.000 |  | 2 | 0.600 | 0.665 | 0.000 |
| 3 | 0.000 | 0.000 | -0.135 |  | 3 | 0.000 | 0.000 | -0.144 |  | 3 | 0.000 | 0.000 | 0.327 |
| ion | 25 |  |  |  | ion | 60 |  |  |  | ion | 95 |  |  |
| 1 | 0.107 | 0.287 | 0.000 |  | 1 | 0.105 | -0.024 | 0.000 |  | 1 | 0.067 | -0.102 | 0.000 |
| 2 | 0.172 | 0.023 | 0.000 |  | 2 | 0.207 | -0.016 | 0.000 |  | 2 | -0.180 | 0.180 | 0.000 |
| 3 | 0.000 | 0.000 | -0.079 |  | 3 | 0.000 | 0.000 | -0.135 |  | 3 | 0.000 | 0.000 | 0.237 |
| ion | 26 |  |  |  | ion | 61 |  |  |  | ion | 96 |  |  |
| 1 | -0.758 | 0.358 | 0.000 |  | 1 | -0.155 | -0.021 | 0.000 |  | 1 | 0.312 | 0.309 | 0.000 |
| 2 | -0.446 | 0.143 | 0.000 |  | 2 | -0.136 | 0.285 | 0.000 |  | 2 | 0.545 | 0.878 | 0.000 |
| 3 | 0.000 | 0.000 | -0.136 |  | 3 | 0.000 | 0.000 | -0.079 |  | 3 | 0.000 | 0.000 | 0.320 |
| ion | 27 |  |  |  | ion | 62 |  |  |  | ion | 97 |  |  |
| 1 | -0.356 | -0.332 | 0.000 |  | 1 | -0.044 | 0.034 | 0.000 |  | 1 | 0.009 | -0.017 | 0.000 |
| 2 | -0.053 | -0.303 | 0.000 |  | 2 | -0.770 | -0.572 | 0.000 |  | 2 | -0.108 | 0.066 | 0.000 |
| 3 | 0.000 | 0.000 | -0.188 |  | 3 | 0.000 | 0.000 | -0.136 |  | 3 | 0.000 | 0.000 | 0.263 |
| ion | 28 |  |  |  | ion | 63 |  |  |  | ion | 98 |  |  |
| 1 | 0.047 | 0.364 | 0.000 |  | 1 | -0.149 | -0.066 | 0.000 |  | 1 | 0.018 | 0.069 | 0.000 |
| 2 | 0.029 | 0.055 | 0.000 |  | 2 | 0.212 | -0.509 | 0.000 |  | 2 | 0.146 | 0.010 | 0.000 |
| 3 | 0.000 | 0.000 | -0.055 |  | 3 | 0.000 | 0.000 | -0.188 |  | 3 | 0.000 | 0.000 | 0.261 |
| ion | 29 |  |  |  | ion | 64 |  |  |  | ion | 99 |  |  |
| 1 | 0.209 | 0.134 | 0.000 |  | 1 | -0.117 | 0.065 | 0.000 |  | 1 | -0.054 | -0.052 | 0.000 |
| 2 | 0.095 | 0.173 | 0.000 |  | 2 | -0.269 | 0.220 | 0.000 |  | 2 | -0.076 | 0.033 | 0.000 |
| 3 | 0.000 | 0.000 | -0.108 |  | 3 | 0.000 | 0.000 | -0.055 |  | 3 | 0.000 | 0.000 | 0.254 |
| ion | 30 |  |  |  | ion | 65 |  |  |  | ion | 100 |  |  |
| 1 | -0.316 | -0.851 | 0.000 |  | 1 | 0.082 | -0.022 | 0.000 |  | 1 | 0.009 | 0.023 | 0.000 |
| 2 | -0.235 | -0.537 | 0.000 |  | 2 | -0.061 | 0.300 | 0.000 |  | 2 | 0.130 | 0.109 | 0.000 |
| 3 | 0.000 | 0.000 | -0.138 |  | 3 | 0.000 | 0.000 | -0.108 |  | 3 | 0.000 | 0.000 | 0.248 |
| ion | 31 |  |  |  | ion | 66 |  |  |  | ion | 101 |  |  |
| 1 | 1.158 | -1.431 | 0.000 |  | 1 | -0.012 | 0.059 | 0.000 |  | 1 | -0.038 | 0.065 | 0.000 |
| 2 | -1.084 | 3.397 | 0.000 |  | 2 | 0.675 | -0.842 | 0.000 |  | 2 | 0.087 | 0.027 | 0.000 |
| 3 | 0.000 | 0.000 | 0.074 |  | 3 | 0.000 | 0.000 | -0.138 |  | 3 | 0.000 | 0.000 | 0.261 |
| ion | 32 |  |  |  | ion | 67 |  |  |  | ion | 102 |  |  |
| 1 | -1.384 | 0.133 | 0.000 |  | 1 | -2.975 | -2.862 | 0.000 |  | 1 | 0.010 | -0.065 | 0.000 |
| 2 | -0.980 | -3.806 | 0.000 |  | 2 | -1.244 | -1.895 | 0.000 |  | 2 | -0.113 | -0.032 | 0.000 |
| 3 | 0.000 | 0.000 | -0.189 |  | 3 | 0.000 | 0.000 | -0.203 |  | 3 | 0.000 | 0.000 | 0.268 |
| ion | 33 |  |  |  | ion | 68 |  |  |  | ion | 103 |  |  |
| 1 | 3.916 | -1.000 | 0.000 |  | 1 | -2.201 | -3.171 | 0.000 |  | 1 | 2.275 | 0.202 | 0.000 |
| 2 | -2.409 | 0.649 | 0.000 |  | 2 | -1.035 | -2.432 | 0.000 |  | 2 | 0.328 | 1.981 | 0.000 |
| 3 | 0.000 | 0.000 | 0.006 |  | 3 | 0.000 | 0.000 | -0.217 |  | 3 | 0.000 | 0.000 | 1.465 |
| ion | 34 |  |  |  | ion | 69 |  |  |  | ion | 104 |  |  |
| 1 | 2.087 | -1.869 | 0.000 |  | 1 | -3.943 | 0.685 | 0.000 |  | 1 | 2.284 | -0.323 | 0.000 |
| 2 | -1.420 | 1.256 | 0.000 |  | 2 | 2.303 | -0.927 | 0.000 |  | 2 | -0.197 | 1.972 | 0.000 |
| 3 | 0.000 | 0.000 | 0.020 |  | 3 | 0.000 | 0.000 | -0.203 |  | 3 | 0.000 | 0.000 | 1.465 |
| ion | 35 |  |  |  | ion | 70 |  |  |  | ion | 105 |  |  |
| 1 | 1.607 | -1.540 | 0.000 |  | 1 | -4.195 | -0.117 | 0.000 |  | 1 | 1.825 | -0.068 | 0.000 |
| 2 | -1.560 | 2.968 | 0.000 |  | 2 | 2.020 | -0.438 | 0.000 |  | 2 | 0.058 | 2.431 | 0.000 |
| 3 | 0.000 | 0.000 | 0.077 |  | 3 | 0.000 | 0.000 | -0.217 |  | 3 | 0.000 | 0.000 | 1.465 |


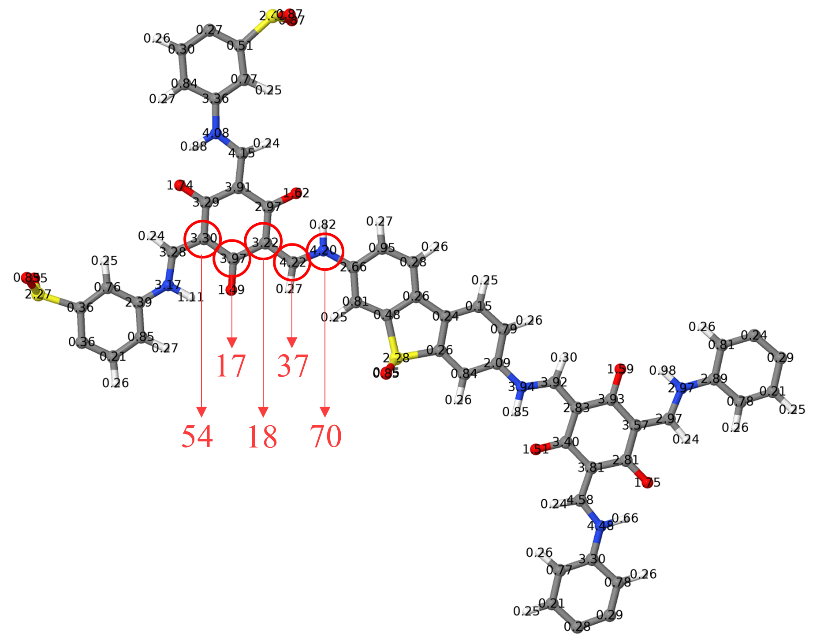


1. Spatial distribution of anomalously high born effective charges ( >3.0 |e|) in S-COF.


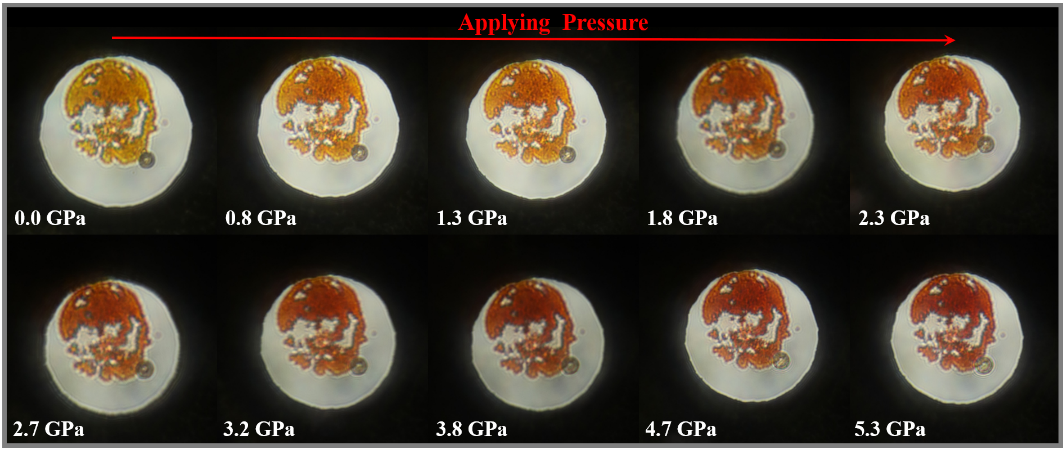


1. *In situ* solid UV-Vis images of 4% MOC-FA3/S-COF under different pressures.


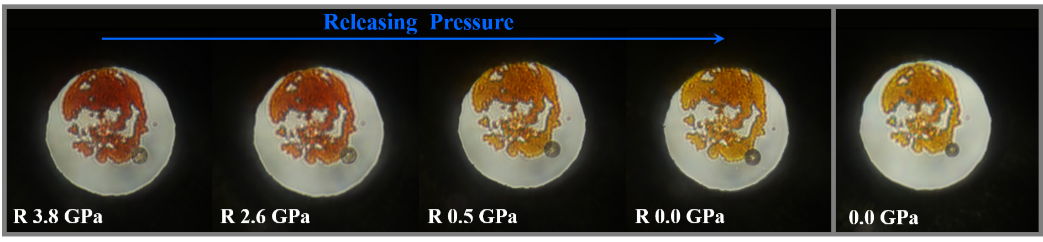


1. *In situ* solid UV-Vis images of 4% MOC-FA3/S-COF by releasing pressure.

1. *In situ* high pressure solid UV-Vis absorption spectra (A) under pressure and (B) by releasing pressure of S-COF; (C) Pressure-dependent band gap and corresponding UV-Vis images of S-COF.


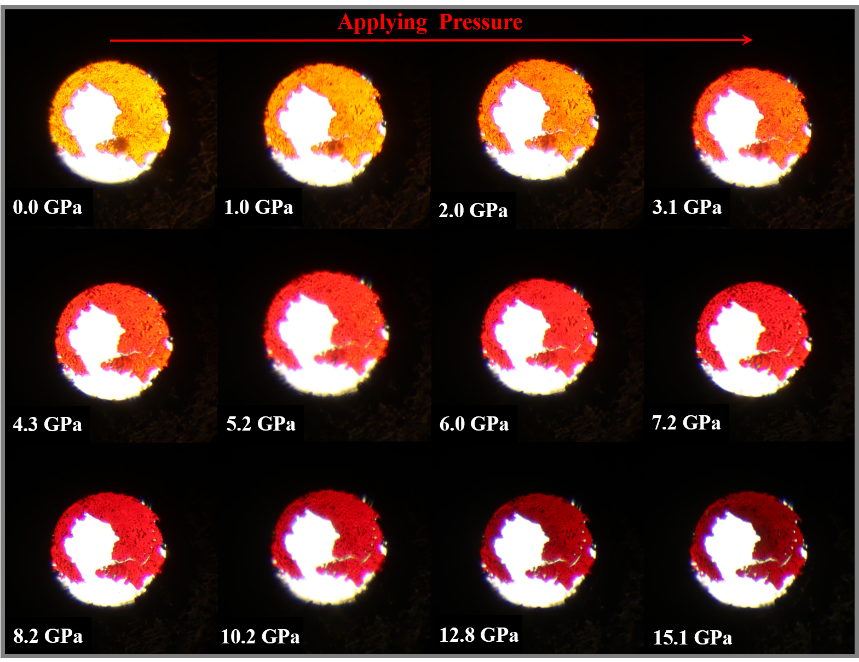


1. *In situ* solid UV-Vis images of S-COF under different pressures.


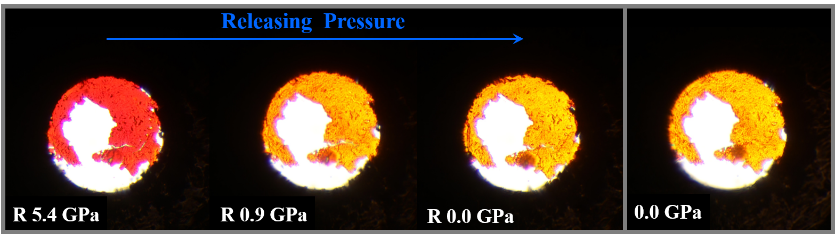


1. *In situ* solid UV-Vis images of S-COF by releasing pressure.

1. *In situ* high pressure solid UV-Vis absorption spectra (A) under pressure and (B) by releasing pressure of MOC-FA3; (C) Pressure-dependent band gap and corresponding UV-Vis images of MOC-FA3.


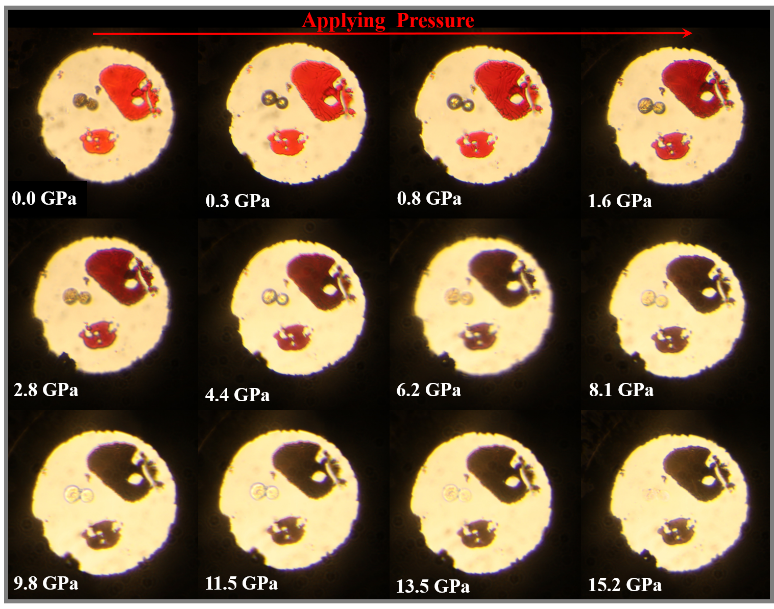


1. *In situ* solid UV-Vis images of MOC-FA3 under different pressures.


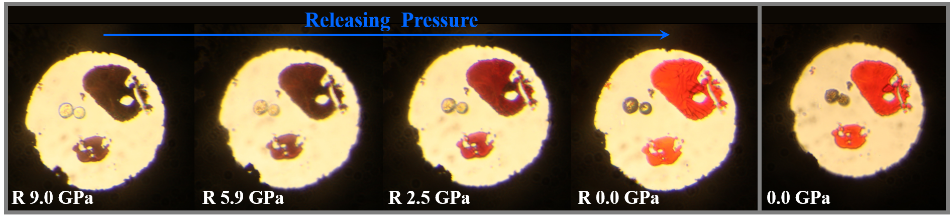


1. *In situ* solid UV-Vis images of MOC-FA3 by releasing pressure.
2. Comparative analysis of reported catalysts for piezo-photocatalytic O_2_/H_2_O_2_ production.

| Catalyst | Mass | Atmosphere | Ultrasonic Power | Ultrasonic Frequency | Light Source | Evolution yield | Ref. |
| --- | --- | --- | --- | --- | --- | --- | --- |
| TAPA-PDA | 5 mg | Ar | 110 W | 40 kHz | - | H_2_: 6.6 mmol/g/h  O_2_: 2.1 mmol/g/h | 8 |
| COF-DH-Eth | 5 mg | O_2_ | 50 W | 40 kHz | 300 W Xe lamp  (λ > 420 nm) | H_2_O_2_: 9212 μmol/g/h | 9 |
| MOC-Q3/EA-COF | 2 mg | Ar | 60 W | 40 kHz | 300 W Xe lamp  (λ > 420 nm) | H_2_: 426.4 μmol/g/h  H_2_O_2_: 535.1 μmol/g/h | 10 |
| ZnIn_2_S_4_-BiOCl | 20 mg | O_2_ | N/A | N/A | 300 W Xe lamp  (λ > 420 nm) | H_2_O_2_: 1987.3 μmol/g/h | 11 |
| NH_2_-UiO-66(Hf)/CdIn_2_S_4_ | 50 mg | O_2_ | 100 W | N/A | 300 W Xe lamp  (λ > 420 nm) | H_2_O_2_: 920 μmol/g/h | 12 |
| E500-CN | 1 mg | Air/N_2_/O_2_ | 80 W | 40 kHz | 120W  LED lamp | H_2_O_2_: 5.3 mmol/g/h (Air)  2.8 mmol/g/h (N_2_)  6.9 mmol/g/h (O_2_) | 13 |
| Au-ZnO | 25 mg | O_2_ | 200 W | 40 kHz | 300 W Xe lamp  (λ > 420 nm) | H_2_O_2_: 7.4 mmol/g/h | 14 |
| (4,4-DFPD)_2_PbI_4_ | 50 mg | O_2_ | 100 W | N/A | 150 W Xe lamp  (λ > 400 nm) | H_2_O_2_: 1185.1 μmol/g/h | 15 |
| C_5_N_2_ | 40 mg | O_2_ | 400 W | 40 kHz | 10 mW/cm^2^  Xe lamp  (λ > 400 nm) | H_2_O_2_: 9184 μmol/g/h | 16 |
| C_3_N_5_-x-CN | 50 mg/L | O_2_ | N/A | N/A | 300 W Xe lamp  (λ > 420 nm) | H_2_O_2_: 1359 μmol/g/h | 17 |
| BiFeO_3_@TpPa-1-COF | 5 mg | N/A | 100 W | 40 kHz | 300 W Xe lamp  (λ > 420 nm) | H_2_: 1416.4 μmol/g/h  O_2_: 708.2 μmol/g/h | 18 |
| 4% MOC-FA3/S-COF | 2 mg | Ar | 60 W | 40 kHz | 300 W Xe lamp  (λ > 420 nm) | H_2_: 1297.6 μmol/g/h  H_2_O_2_: 1304.2 μmol/g/h | This work |
|  |  |  | 120 W | 40 kHz |  | H_2_: 4089.6 μmol/g/h  H_2_O_2_: 3985.4 μmol/g/h |  |

1. Comparative analysis of reported catalysts for piezo-photocatalytic H_2_ production.

| Catalyst | Mass | Sacrificial agent | Ultrasonic Power | Ultrasonic Frequency | Light Source | Evolution yield | Ref. |
| --- | --- | --- | --- | --- | --- | --- | --- |
| TAPA-PDA | 5 mg | - | 110 W | 40 kHz | - | H_2_: 6.6 mmol/g/h  O_2_: 2.1 mmol/g/h | 8 |
| MOC-Q3/EA-COF | 2 mg | - | 60 W | 40 kHz | 300 W Xe lamp  (λ > 420 nm) | H_2_: 426.4 μmol/g/h  H_2_O_2_: 535.1 μmol/g/h | 10 |
| BiFeO_3_@TpPa-1-COF | 5 mg | - | 100 W | 40 kHz | 300 W Xe lamp  (λ > 420 nm) | H_2_: 1416.4 μmol/g/h  O_2_: 708.2 μmol/g/h | 18 |
| Bi_2_Fe_4_O_9_ nanoplates | 2 mg | - | 200 W | 40 kHz | - | H_2_: 1058 μmol/g/h | 19 |
| MoC@NG | 2 mg | - | 100 W | 40 kHz | - | H_2_: 1690 μmol/g/h | 20 |
| 1 *w*t% Pt@Sn_0.97_Ag_0.03_S_2_ | 10 mg | 0.05 M Na_2_SO_3_ | 600 W | 45 kHz | - | H_2_: 390 μmol/g/h | 21 |
| BMO-0.1BTO | 50 mg | 10 *vol*% methanol | 300 W | 99 kHz | - | H_2_: 152.6 μmol/g/h | 22 |
| MCC-200 | 50 mg | 10 *vol*% methanol | 300 W | 75 kHz | - | H_2_: 84.5 μmol/g/h | 23 |
| BiOBr | 20 mg | - | 40 W | 40 kHz | - | H_2_: 813 μmol/g/h | 24 |
| *r*-FAPbBr_3-x_I_x_ | 100 mg | 50 *w*t% H_3_PO_2_ | 112 W | 40 kHz | - | H_2_: 157.8 μmol/g/h | 25 |
| BTO@MoSe_2_ | 10 mg | 25 *vol*% methanol | 300 W | 40 kHz | 150 W Xe lamp | H_2_: 4533 μmol/g/h | 26 |
| 4% MOC-FA3/S-COF | 2 mg | - | 60 W | 40 kHz | 300 W Xe lamp  (λ > 420 nm) | H_2_: 1297.6 μmol/g/h  H_2_O_2_: 1304.2 μmol/g/h | This work |
|  |  |  | 120 W | 40 kHz |  | H_2_: 4089.6 μmol/g/h  H_2_O_2_: 3985.4 μmol/g/h |  |

1. Comparative performance of piezo-photocatalytic systems. (A) H_2_ and H_2_O_2_/O_2_ production; (B) H_2_ production.

1. (A) Solid UV-Vis absorption spectra, (B) FT-IR spectra, and (C) XPS spectra of 4% MOC-FA3/S-COF before and after 15 h of continuous piezo-photocatalysis.


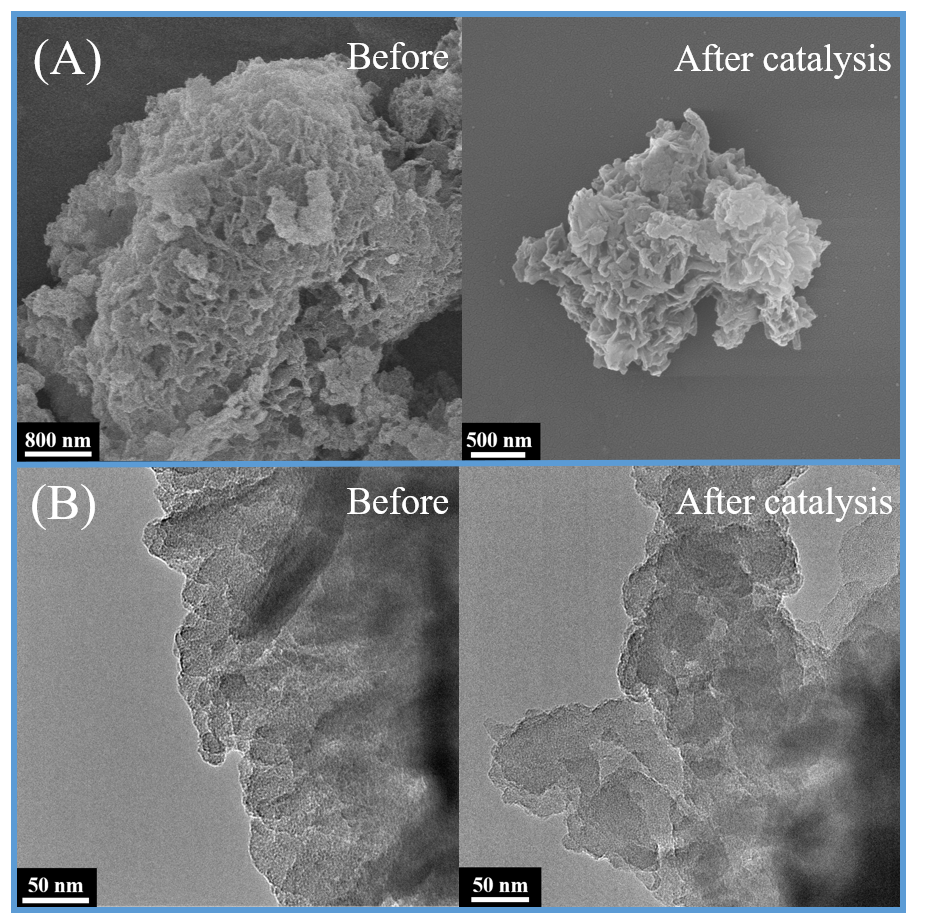


1. (A) SEM and (B) TEM images of 4% MOC-FA3/S-COF before and after 15 h of continuous piezo-photocatalysis.

1. Piezocatalytic H_2_ and H_2_O_2_ by (A) S-COF and (B) MOC-FA3 under different ultrasonication frequencies on 60 W in 0.5 h.

1. Photocurrent profiles of (A) MOC-FA3 and (B) S-COF under ultrasound, light and both ultrasound and light.

1. Photocurrent profiles of comparison of all samples under both ultrasound and light.


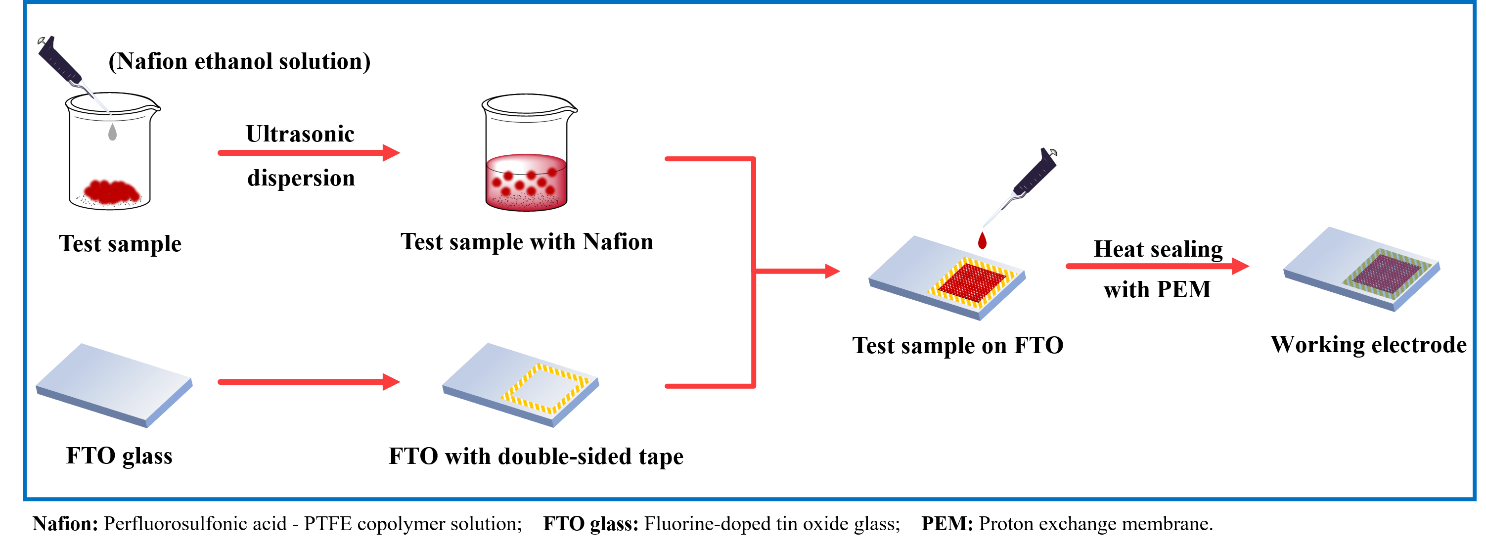


1. Preparation process of working electrode for piezo-photoelectrochemical testing.

1. EIS Nyquist plots of (A) MOC-FA3, (B) S-COF and (C) 4% MOC-FA3/S-COF under ligth or both ultrasound and light; (D) Comparison EIS Nyquist plots of MOC-FA3, S-COF and 4% MOC-FA3/S-COF under ultrasound and light (R_1_: Series resistance; R_2_: Charge-transfer resistance; CPE_1_: Constant phase element).
2. .
3. Fitting results of ElS Nyquist plots for MOC-FA3, S-COF and 4% MOC-FA3/S-COF under the synergistic effect of light irradiation (L) and ultrasound (U).

| Sample | Testing Condition | R_2_ (Ω) | Error (%) |
| --- | --- | --- | --- |
| MOC-FA3 | U+L | 3175 | 7.05 |
|  | U | 3664 | 8.94 |
| S-COF | U+L | 2871 | 5.18 |
|  | U | 4255 | 4.45 |
| 4% MOC-FA3  /S-COF | U+L | 1463 | 4.58 |
|  | U | 2109 | 4.50 |


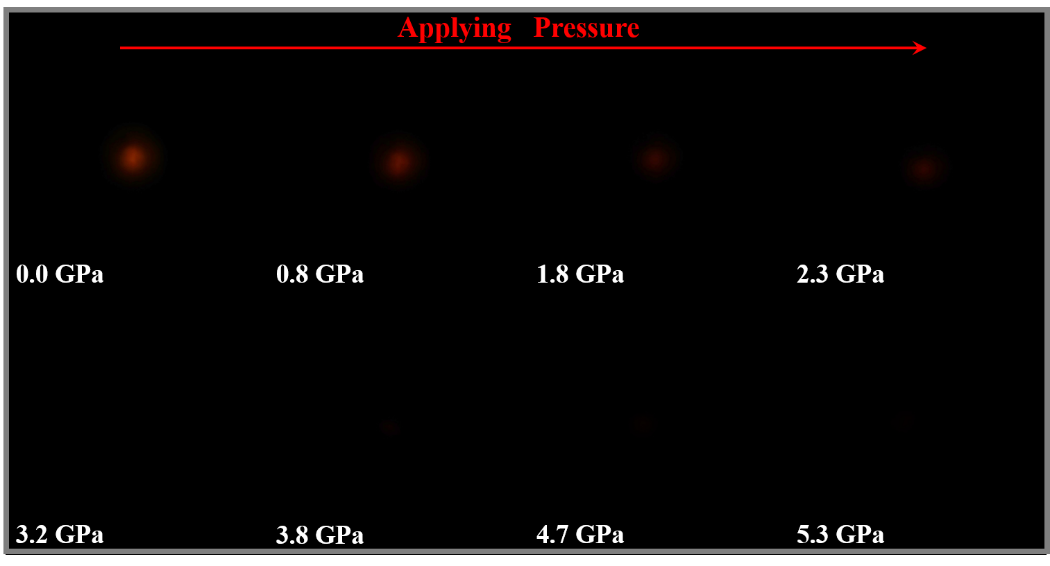


1. *In situ* PL images of 4% MOC-FA3/S-COF under different pressures.


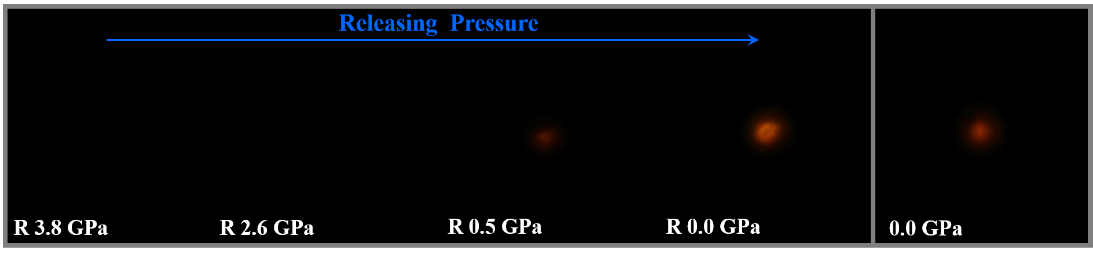


1. *In situ* PL images of 4% MOC-FA3/S-COF by releasing pressure.

1. *In situ* high pressure PL spectra (A) under pressure and (B) by releasing pressure of S-COF; (C) Pressure-dependent PL peak position (green line), PL peak intensity (orange line) and corresponding PL images of S-COF.

1. *In situ* high pressure PL spectra (A) under pressure and (B) by releasing pressure of MOC-FA3; (C) Pressure-dependent PL peak position (green line), PL peak intensity (orange line) and corresponding PL images of MOC-FA3.


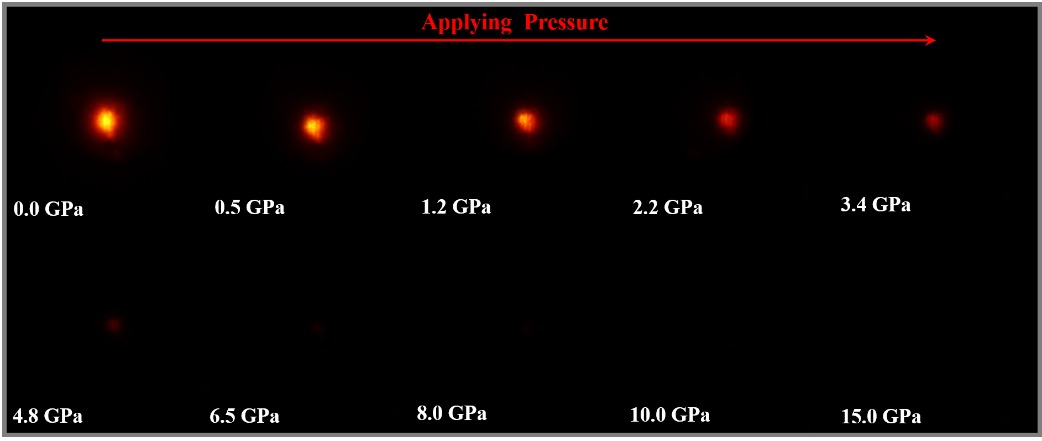


1. *In situ* PL images of S-COF under different pressures.


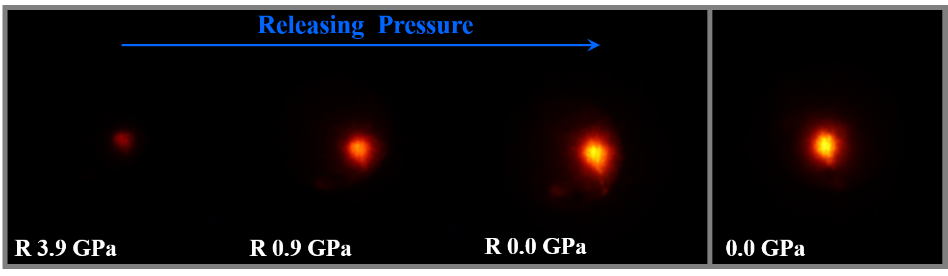


1. *In situ* PL images of S-COF by releasing pressure.


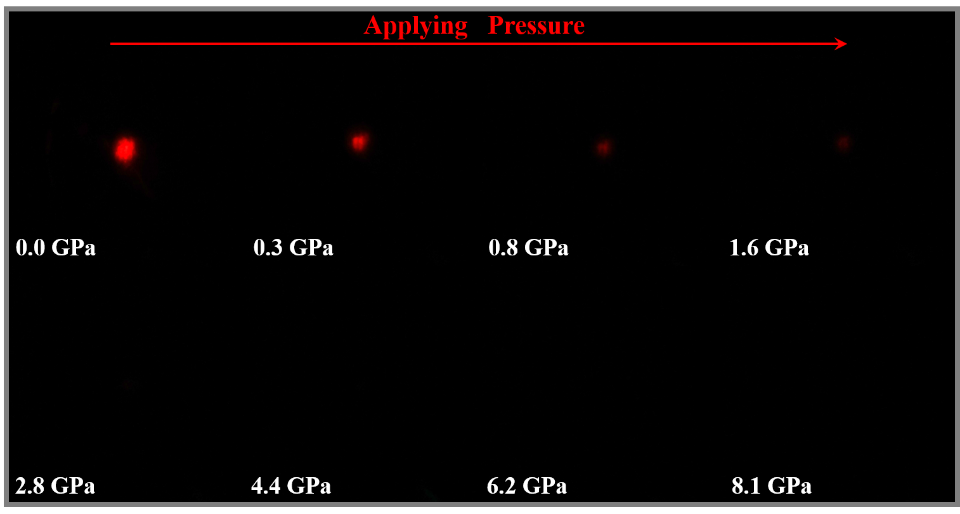


1. *In situ* PL images of MOC-FA3 under different pressures.


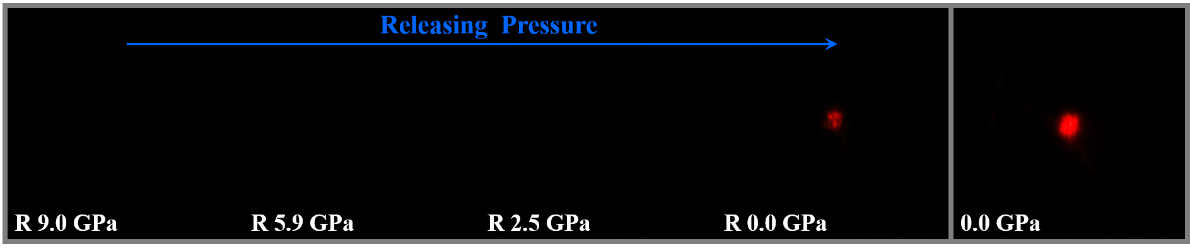


1. *In situ* PL images of MOC-FA3 by releasing pressure.

1. The variation trend of pressure-dependent fluorescence intensity of S-COF, MOC-FA3 and 4% MOC-FA3/S-COF

1. Multiscale characterization of 4% MOC-FA3/S-COF. (A) 3D topographical mapping; (B) Surface morphology analysis; (C) Light-triggered surface potential response.

1. Multiscale characterization of MOC-FA3. (A) 3D topographical mapping; (B) Surface morphology analysis; (C) Light-triggered surface potential response; (D) Corresponding potential distribution map.

1. Multiscale characterization of S-COF. (A) 3D topographical mapping; (B) Surface morphology analysis; (C) Light-triggered surface potential response; (D) Corresponding potential distribution map.

1. Radical trapping (0.5 h) investigation for piezo-photocatalytic mechanisms over 4% MOC-FA3/S-COF.

1. Isotopic verification of hydroxyl radical (•OH) origin via GC-MS by 4% MOC-FA3/S-COF. (A) H_2_O system: m/z signals for •OH adducts; (B) H_2_^18^O system: m/z shift to •^18^OH products.


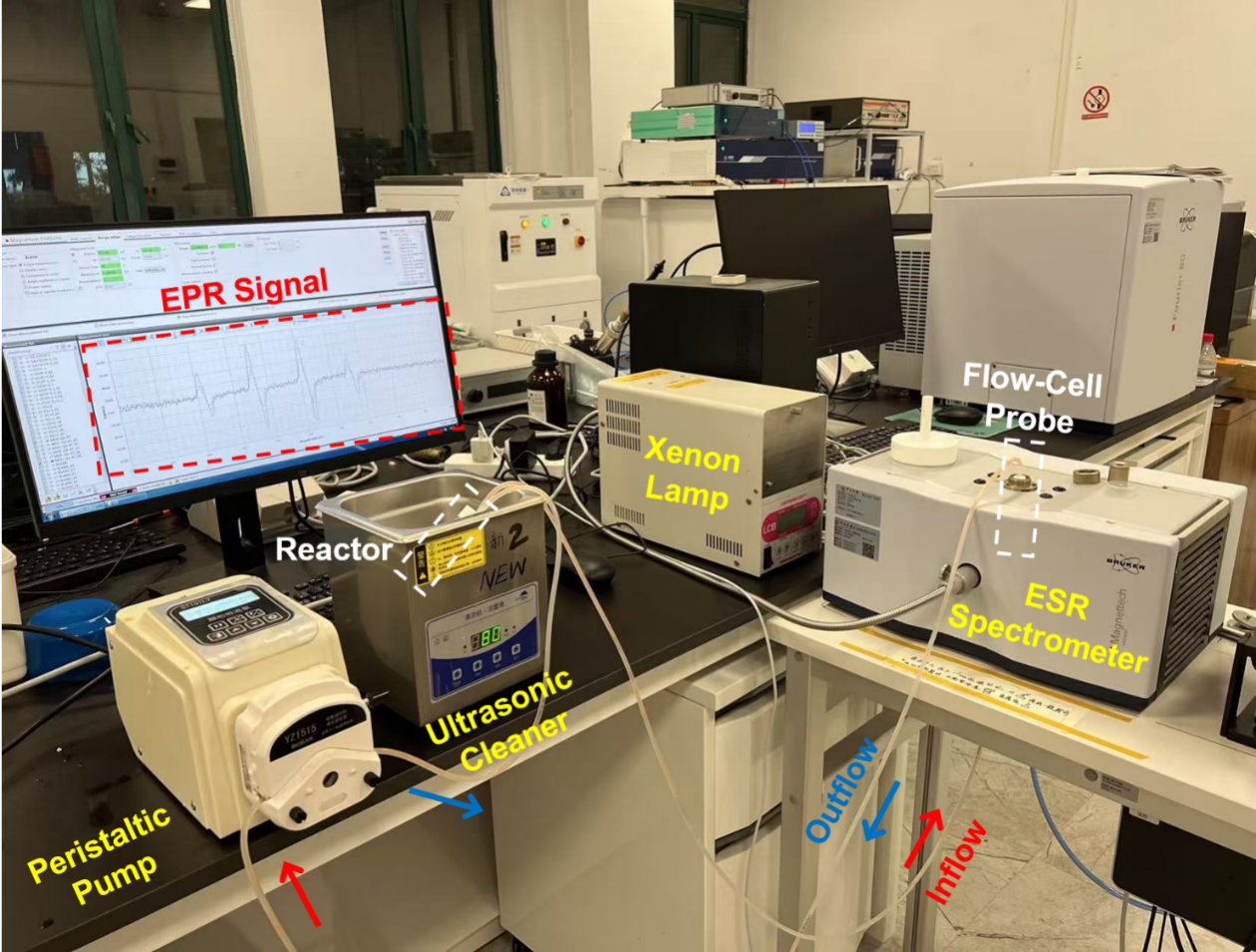


1. Photographs of in-situ EPR experimental systems using a ultrasonic cleaner and Xe lamp for piezo-catalytic reaction.

1. Time-dependent liquid PL spectra of TA-OH under 315 nm excitation for (A) MOC-FA3, (B) S-COF, and (C) 4% MOC-FA3/S-COF; (D) Comparison of liquid PL intensity for the three samples at 75 min under simultaneous ultrasound and irradiation.

1. Time-dependent liquid PL intensity of TA-OH under 315 nm excitation for MOC-FA3, S-COF, and 4% MOC-FA3/S-COF under simultaneous ultrasound and irradiation.


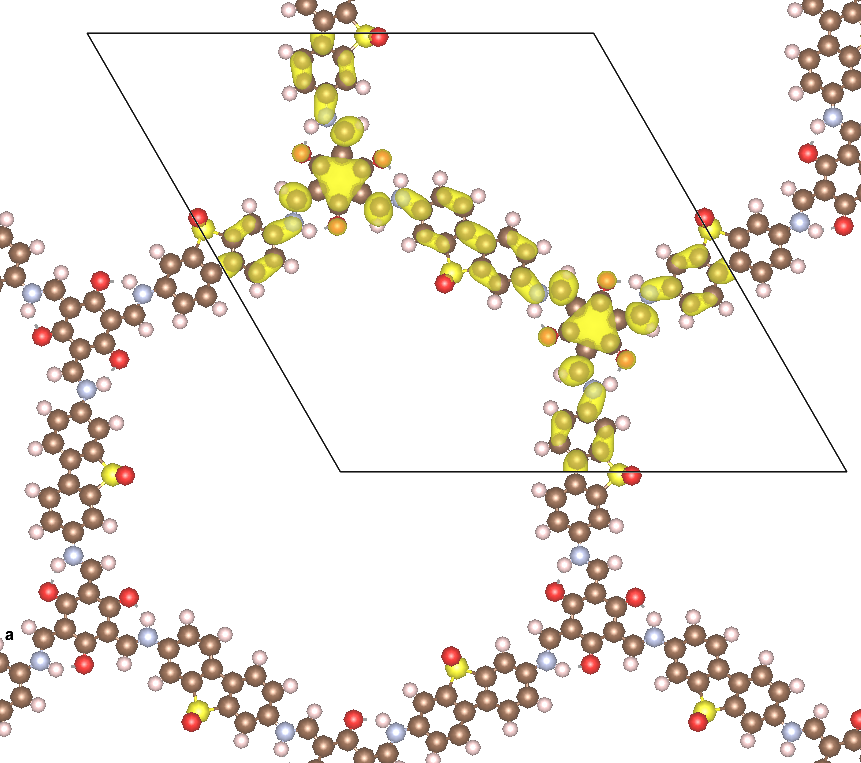


1. Conduction band spatial distribution in S-COF.
2. Site-specific adsorption free energy of H^+^ on S-COF.

| Sites | 1 | 2 | 3 | 4 | 5 |
| --- | --- | --- | --- | --- | --- |
| G ads (eV) | 1.867 | 1.552 | 1.126 | Not stable | 1.772 |
|  |  |  |  |  |  |
| Sites | 6 | 7 | 8 | 9 | 10 |
| G ads (eV) | 1.187 | 1.091 | 1.352 | 1.322 | 1.574 |

1. Calculated H^+^ adsorption free energy barriers (ΔG) at specific sites on S-COF for the HER.


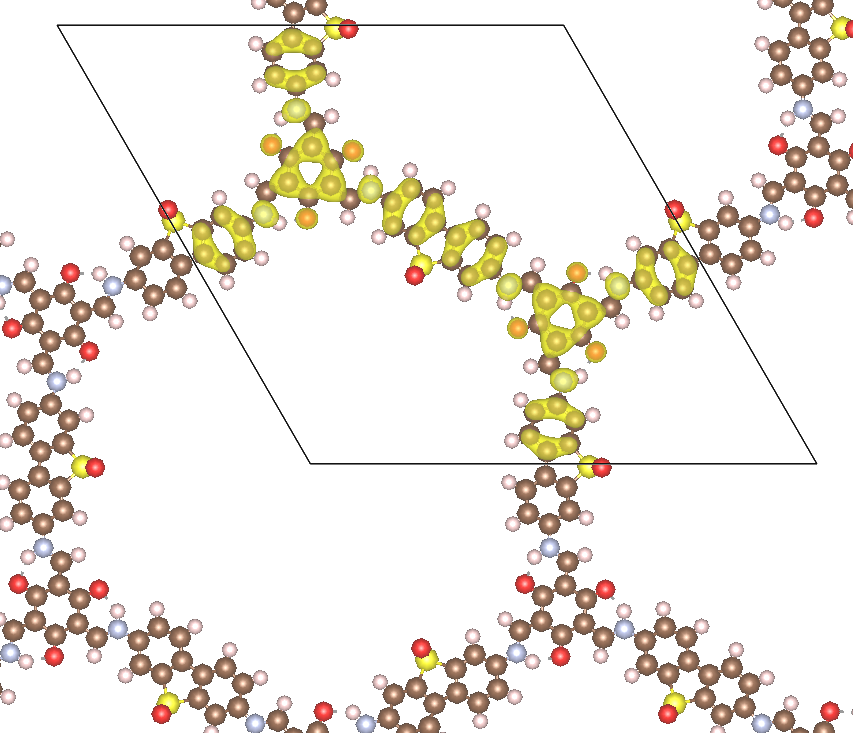


1. Valence band spatial distribution in S-COF.
2. Site-specific adsorption free energy of OH^-^ on S-COF.

| Sites | 1 | 2 | 3 | 4 | 5 |
| --- | --- | --- | --- | --- | --- |
| G ads (eV) | 2.506 | 2.559 | 1.949 | 3.319 | 2.056 |
|  |  |  |  |  |  |
| Sites | 6 | 7 | 8 | 9 | 10 |
| G ads (eV) | 2.147 | 2.053 | 2.400 | 2.318 | 2.449 |

1. Calculated OH^-^ adsorption free energy barriers (ΔG) at specific sites on S-COF for the OER and H_2_O_2_ evolution reactions.


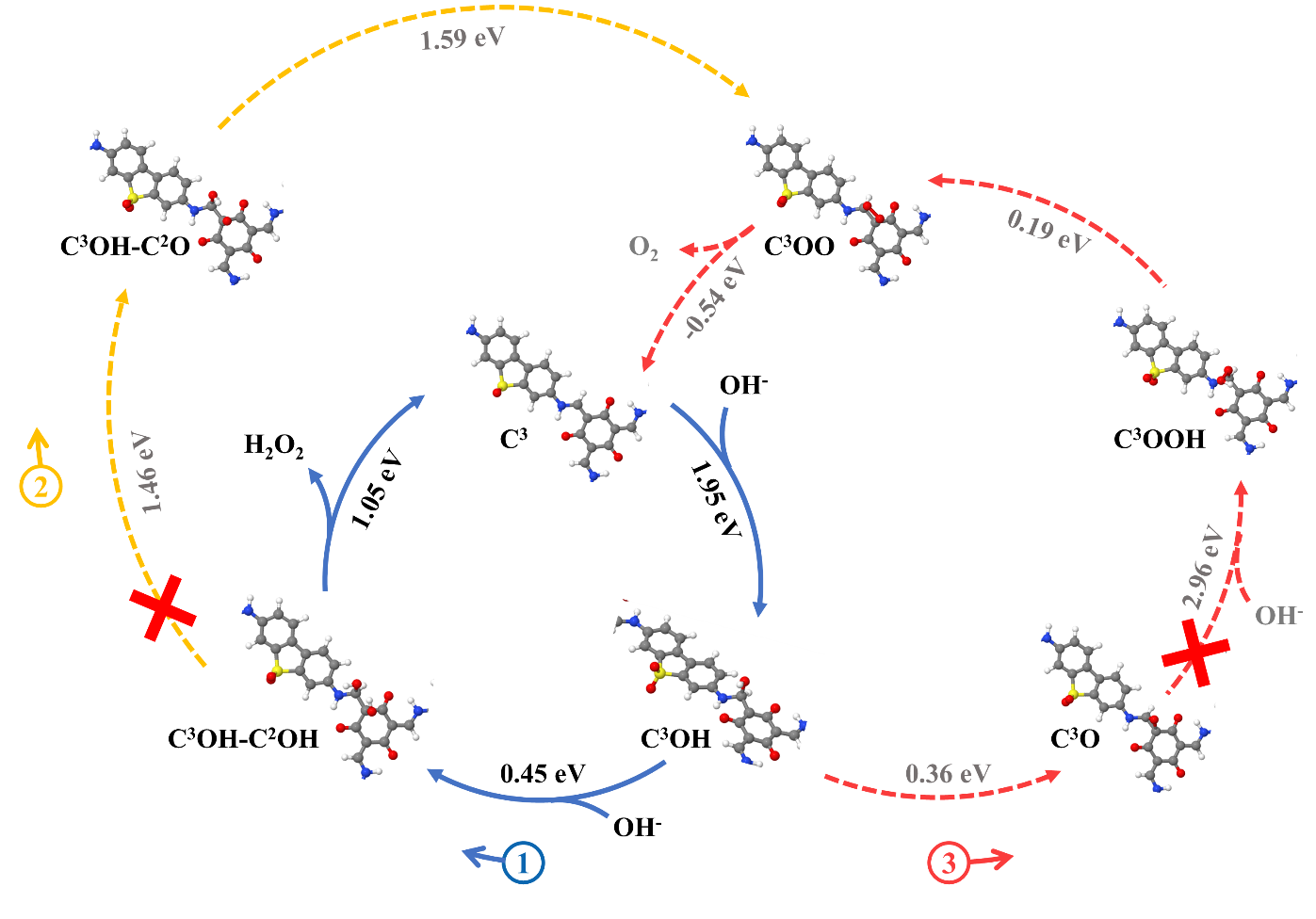


1. Proposed reaction pathways for H_2_O_2_ formation and O_2_ evolution on S-COF.


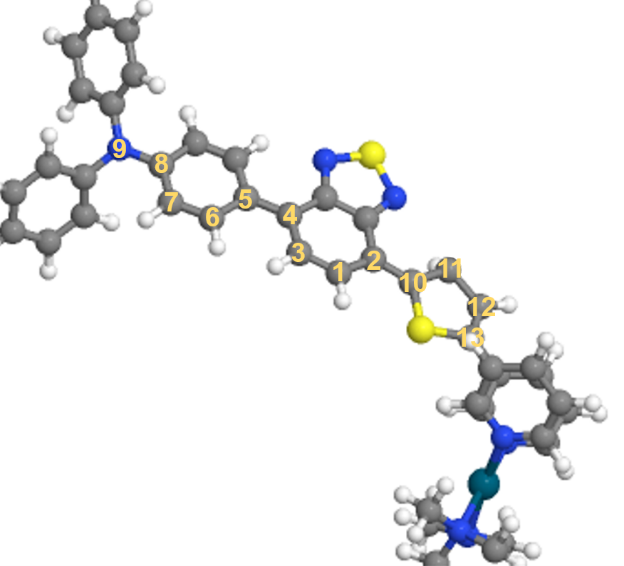


1. Structure of MOC-FA3 and identified adsorption sites.
2. Site-specific adsorption free energy of OH^-^ on MOC-FA3.

| Sites | 1 | 2 | 3 | 4 | 5 |
| --- | --- | --- | --- | --- | --- |
| G ads (eV) | 1.704 | 1.689 | 1.541 | 1.966 | 1.796 |
|  |  |  |  |  |  |
| Sites | 6 | 7 | 8 | 9 | 10 |
| G ads (eV) | 1.948 | 1.734 | 2.176 | 2.825 | 1.879 |
|  |  |  |  |  |  |
| Sites | 11 | 12 | 13 |  |  |
| G ads (eV) | 1.478 | 1.764 | 1.510 |  |  |

1. Calculated OH^-^ adsorption free energy barriers (ΔG) at specific sites on MOC-FA3 for the OER and H_2_O_2_ evolution reactions.

1. (A) Gibbs free energy diagrams for the H_2_O_2_ evolution reaction at different C-C reaction sites on MOC-FA3; (B) Gibbs free energy diagrams for both the OER and H_2_O_2_ evolution reaction at the C^11^-C^12^ site on MOC-FA3.


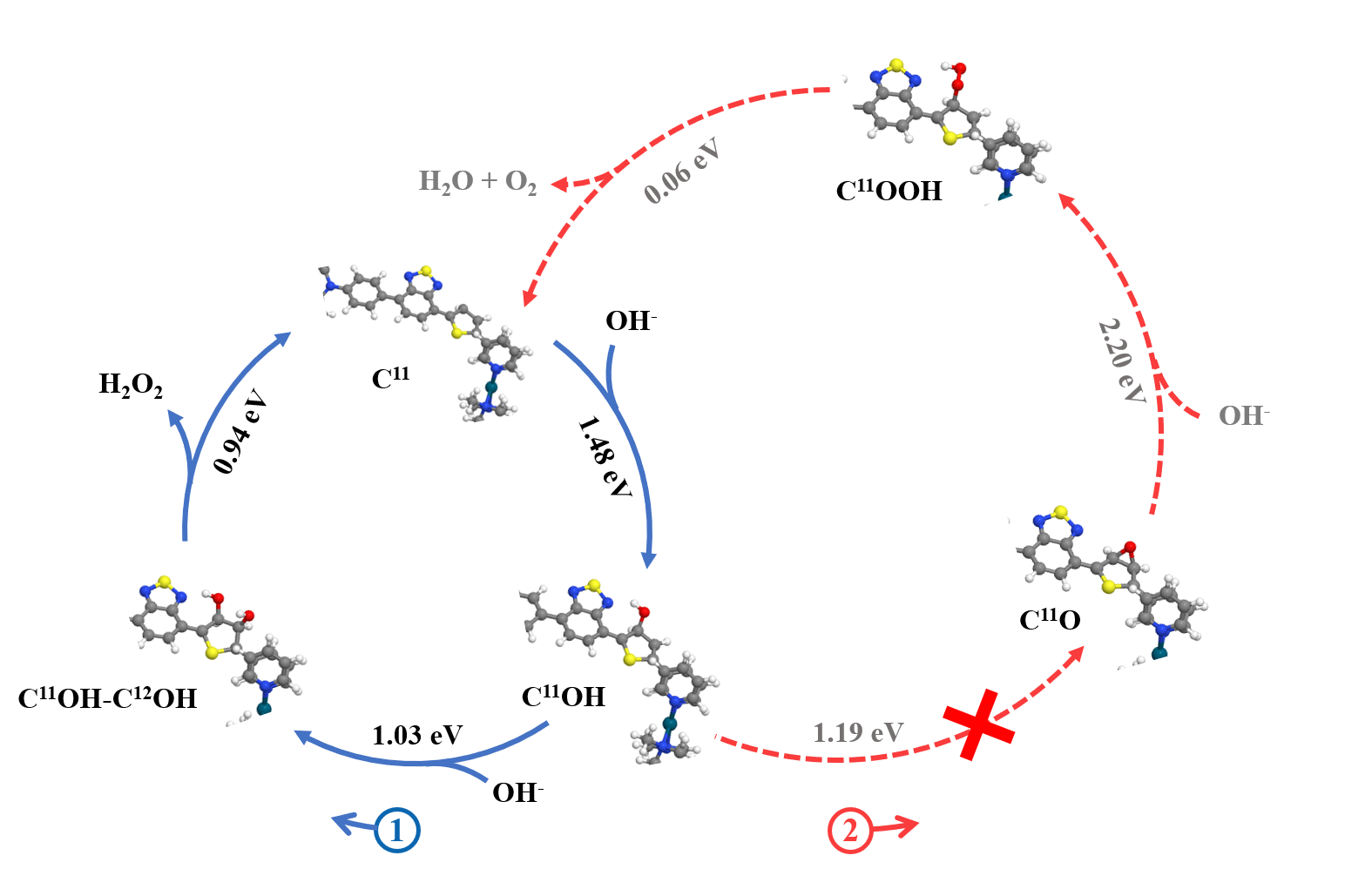


1. Proposed reaction pathways on MOC-FA3: one for H_2_O_2_ formation and one for O_2_ evolution on the catalyst.


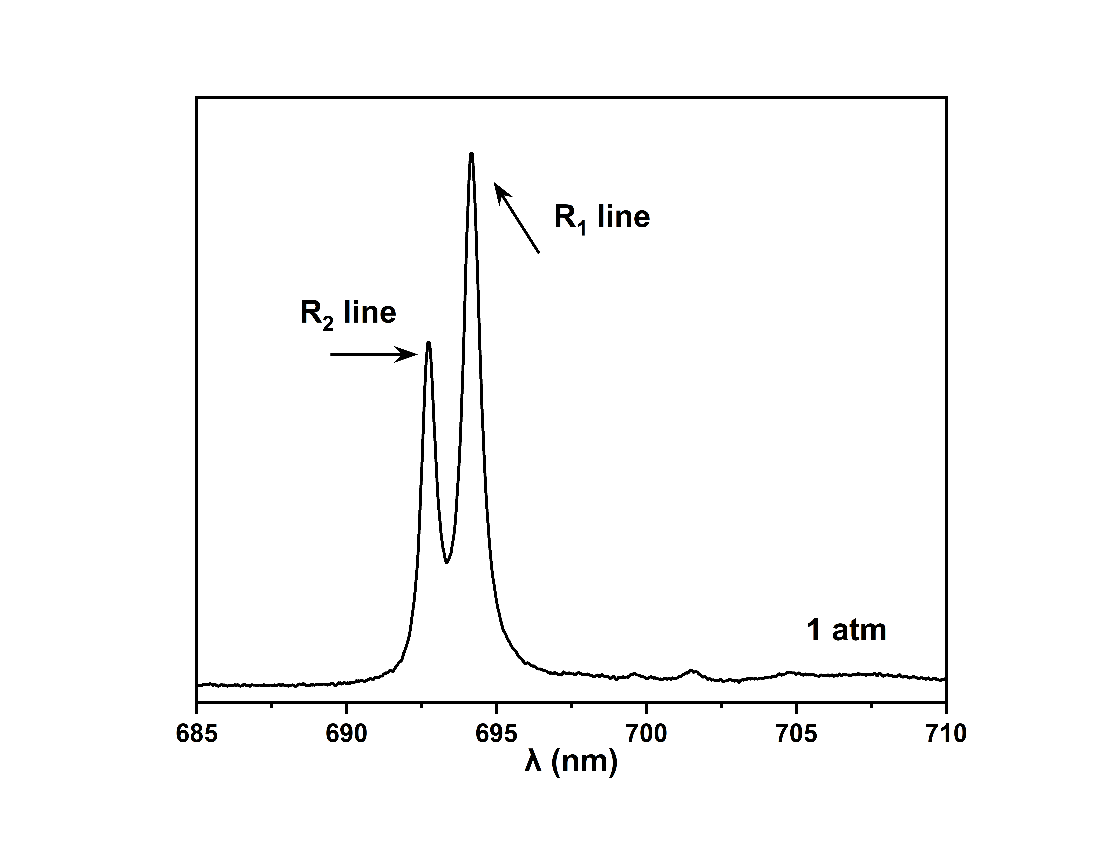


1. The fluorescence spectrum of the ruby.

References

1. Kresse, and Furthmuller, "Efficient Iterative Schemes for Ab Initio Total-Energy Calculations Using a Plane-Wave Basis Set," Physical review. B, Condensed matter 54 (1996): 11169-11186. https://doi.org/10.1103/PhysRevB.54.11169.

2. G. Kresse, and J. Furthmuller, "Efficiency of Ab-Initio Total Energy Calculations for Metals and Semiconductors Using a Plane-Wave Basis Set," Computational materials science 6 (1996): 15-50. https://doi.org/10.1016/0927-0256(96)00008-0.

3. J. P. Perdew, K. Burke, and M. Ernzerhof, "Generalized Gradient Approximation Made Simple (Vol 77, Pg 3865, 1996)," Physical review letters 78 (1997): 1396–1396.

4. J. P. Perdew, K. Burke, and M. Ernzerhof, "Generalized Gradient Approximation Made Simple," Physical review letters 77 (1996): 3865-3868. https://doi.org/10.1103/PhysRevLett.77.3865.

5. S. Grimme, J. Antony, S. Ehrlich, and H. Krieg, "A Consistent and Accurate Ab Initio Parametrization of Density Functional Dispersion Correction (DFT-D) for the 94 Elements H-Pu," Journal of Chemical Physics 132 (2010): 19. https://doi.org/10.1063/1.3382344.

6. K. Momma, and F. Izumi, "Vesta 3 for Three-Dimensional Visualization of Crystal, Volumetric and Morphology Data," Journal of applied crystallography 44 (2011): 1272-1276. https://doi.org/10.1107/s0021889811038970.

7. R. M. Hanson, J. Prilusky, Z. Renjian, T. Nakane, and J. L. Sussman, "Jsmol and the Next-Generation Web-Based Representation of 3D Molecular Structure as Applied to Proteopedia," Israel Journal of Chemistry 53 (2013): 207-216. https://doi.org/10.1002/ijch.201300024.

8. A. Ghosh, S. Menon, S. Biswas, et al., "Ferrielectric Dipolar Ordering in a Donor-Acceptor Based Covalent-Organic Framework for Piezocatalytic Water Splitting," Advanced Functional Materials (2025): 2502787. https://doi.org/10.1002/adfm.202502787.

9. Z. Li, Z. Dong, Z. Zhang, et al., "Covalent Organic Frameworks for Boosting H_2_O_2_ Photosynthesis via the Synergy of Multiple Charge Transfer Channels and Polarized Field," Angewandte Chemie International Edition 64 (2025): e202420218. https://doi.org/10.1002/anie.202420218.

10. Z.-Z. Liang, X.-A. Li, Q.-Z. Chen, et al., "A Direct Z-Scheme Single-Atom MOC/COF Piezo-Photocatalytic System for Overall Water Splitting," Acs Catalysis 14 (2024): 10447-10461. https://doi.org/10.1021/acscatal.4c02243.

11. X. Sun, K. Lv, F. Liu, et al., "Insight into Interfacial Engineering for Enhancing the Synergistic Effect of Piezo-Photocatalytic Hydrogen Peroxide Production," Chemical Engineering Journal 497 (2024): 154504. https://doi.org/10.1016/j.cej.2024.154504.

12. Z. Guo, C. Zhao, L. Meng, et al., "Unraveling Synergistic Effect of In-O-Hf Bond and Piezo-Photocatalysis in Simultaneous H_2_O_2_ Production and Diclofenac Sodium Degradation," Applied Catalysis B: Environment and Energy 377 (2025): 125507. https://doi.org/10.1016/j.apcatb.2025.125507.

13. X. Yan, J. Zhu, Y. Liu, et al., "Enhanced Piezo-Phototronic Effect in Carbon Nitride Nanosheets via Oxidative Exfoliation for High-Efficiency Piezo-Photocatalysis," Journal of Materials Chemistry A 13 (2025): 4948-4959. https://doi.org/10.1039/D4TA07713J.

14. Y. Wen, H. Che, C. Tang, B. Liu, and Y. Ao, "A Schottky Heterojunction with Spatially Separated Active Sites for Piezo-Photocatalytic Dual-Channel Hydrogen Peroxide Generation," Nano Energy 128 (2024): 109837. https://doi.org/10.1016/j.nanoen.2024.109837.

15. Z. Zhou, Y. Yang, P. Cao, S. Zhang, and Q. Ye, "Spontaneous Polarization Halide Perovskite Piezo-Photocatalysis for Highly Efficient Photocatalytic Hydrogen Evolution," Journal of Colloid and Interface Science 685 (2025): 1018-1026. https://doi.org/10.1016/j.jcis.2025.01.211.

16. J. Ma, C. Peng, X. Peng, et al., "H_2_O_2_ Photosynthesis from H_2_O and O_2_ under Weak Light by Carbon Nitrides with the Piezoelectric Effect," Journal of the American Chemical Society 146 (2024): 21147-21159. https://doi.org/10.1021/jacs.4c07170.

17. C. Fu, M. Zhao, X. Chen, et al., "Unraveling the Dual Defect Effects in C_3_N_5_ for Piezo-Photocatalytic Degradation and H_2_O_2_ Generation," Applied Catalysis B: Environmental 332 (2023): 122752. https://doi.org/10.1016/j.apcatb.2023.122752.

18. M.-L. Xu, M. Lu, G.-Y. Qin, et al., "Piezo-Photocatalytic Synergy in BiFeO_3_@COF Z-Scheme Heterostructures for High-Efficiency Overall Water Splitting," Angewandte Chemie International Edition 61 (2022): e202210700. https://doi.org/10.1002/anie.202210700.

19. Y. Du, T. Lu, X. Li, et al., "High-Efficient Piezocatalytic Hydrogen Evolution by Centrosymmetric Bi_2_Fe_4_O_9_ Nanoplates," Nano Energy 104 (2022): 107919. https://doi.org/10.1016/j.nanoen.2022.107919.

20. W. Feng, J. Yuan, F. Gao, et al., "Piezopotential-Driven Simulated Electrocatalytic Nanosystem of Ultrasmall MoC Quantum Dots Encapsulated in Ultrathin N-Doped Graphene Vesicles for Superhigh H_2_ Production from Pure Water," Nano Energy 75 (2020): 104990. https://doi.org/10.1016/j.nanoen.2020.104990.

21. W. Tian, J. Han, L. Wan, et al., "Enhanced Piezocatalytic Activity in Ion-Doped SnS_2_ via Lattice Distortion Engineering for BPA Degradation and Hydrogen Production," Nano Energy 107 (2023): 108165. https://doi.org/10.1016/j.nanoen.2023.108165.

22. K. Zhang, X. Sun, H. Wang, et al., "Interfacial Engineering of Bi_2_MoO_6_-BaTiO_3_ Type-I Heterojunction Promotes Cocatalyst-Free Piezocatalytic H_2_ Production," Nano Energy 121 (2024): 109206. https://doi.org/10.1016/j.nanoen.2023.109206.

23. K. Zhang, X. Sun, H. Hu, et al., "Defect Engineered Microcrystalline Cellulose for Enhanced Cocatalyst-Free Piezo-Catalytic H_2_ Production," Small 19 (2023): 2304674. https://doi.org/10.1002/smll.202304674.

24. H.-R. Li, Y.-M. Chen, C.-C. Jin, et al., "High-Performance Piezocatalytic Hydrogen Evolution over Bismuth Oxyhalides with Halogen-Dependent Piezoelectricity and Surface Activity," Rare Metals 44 (2025): 5475-5485. https://doi.org/10.1007/s12598-025-03297-9.

25. X. Mo, M. Wang, H. Song, et al., "Upcycling of Pb from Lead Smelting Residues to Lead Halide Perovskite for Piezocatalytic Hydrogen Production," Chemical Engineering Journal 498 (2024): 155105. https://doi.org/10.1016/j.cej.2024.155105.

26. S.-L. Guo, S.-N. Lai, and J. M. Wu, "Strain-Induced Ferroelectric Heterostructure Catalysts of Hydrogen Production through Piezophototronic and Piezoelectrocatalytic System," ACS Nano 15 (2021): 16106-16117. https://doi.org/10.1021/acsnano.1c04774.
